# In Situ Assembly of Platinum(II)-Metallopeptide Nanostructures Disrupts Energy Homeostasis and Cellular Metabolism

**DOI:** 10.1021/jacs.2c03215

**Published:** 2022-06-22

**Authors:** Zhixuan Zhou, Konrad Maxeiner, Pierpaolo Moscariello, Siyuan Xiang, Yingke Wu, Yong Ren, Colette J. Whitfield, Lujuan Xu, Anke Kaltbeitzel, Shen Han, David Mücke, Haoyuan Qi, Manfred Wagner, Ute Kaiser, Katharina Landfester, Ingo Lieberwirth, David Y.W. Ng, Tanja Weil

**Affiliations:** † Max Planck Institute for Polymer Research, 55128 Mainz, Germany; ‡ Central Facility of Materials Science Electron Microscopy, Universität Ulm, 89081 Ulm, Germany; § Faculty of Chemistry and Food Chemistry & Center for Advancing Electronics Dresden (cfaed), Technische Universität Dresden, 01062 Dresden, Germany

## Abstract

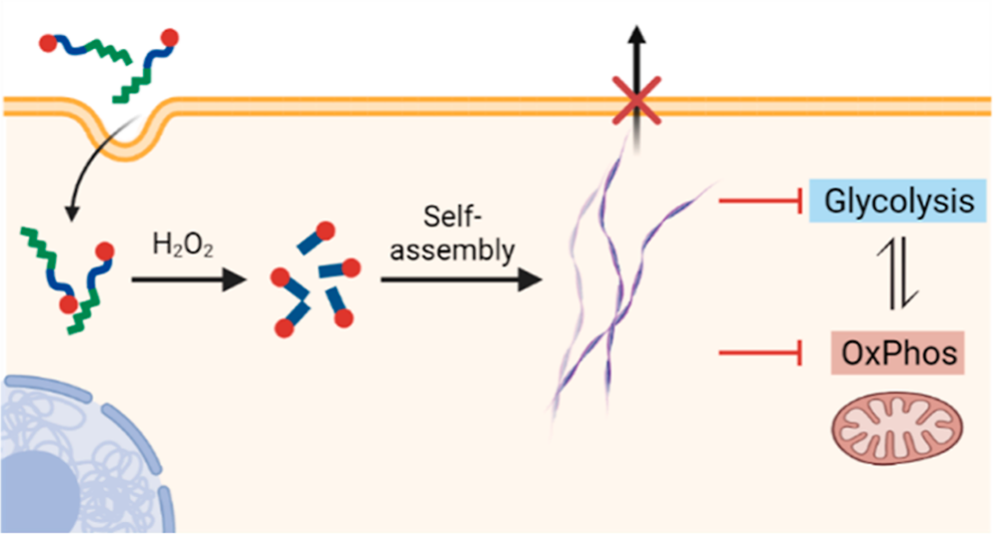

Nanostructure-based functions are omnipresent in nature and essential for the diversity of life. Unlike small molecules, which are often inhibitors of enzymes or biomimetics with established methods of elucidation, we show that functions of nanoscale structures in cells are complex and can implicate system-level effects such as the regulation of energy and redox homeostasis. Herein, we design a platinum(II)-containing tripeptide that assembles into intracellular fibrillar nanostructures upon molecular rearrangement in the presence of endogenous H_2_O_2_. The formed nanostructures blocked metabolic functions, including aerobic glycolysis and oxidative phosphorylation, thereby shutting down ATP production. As a consequence, ATP-dependent actin formation and glucose metabolite-dependent histone deacetylase activity are downregulated. We demonstrate that assembly-driven nanomaterials offer a rich avenue to achieve broad-spectrum bioactivities that could provide new opportunities in drug discovery.

## Introduction

In cells, protein nanostructures are abundant, serving as cellular scaffolds, multi-domain catalysts, and transport highways.^1^ Among them, ordered protein assemblies consisting of β-sheet structures, the so-called β-amyloids, play both functional and pathological roles. They serve as biotemplates within melanocytes for melanin biosynthesis^2,3^ or as nucleating centers toward the amyloidosis in Type 2 diabetes.^4,5^ Mechanistically, the monomers of these ordered nanostructures do not possess intrinsic functions of their own but rely on the propagation of long-range hierarchical structures to feature their rich biological activities. Several strategies have since been attempted to re-engineer these nanostructures using simplified peptide-based monomers to elicit biomedical functions.^6−13^ Pharmacologically, self-assembling nanostructures in cells combine features of small molecules such as deep cell/tissue penetration with properties from larger superstructures like enzymatic stability and retardation of cellular efflux.^10,14,15^ Existing studies, with a major focus on native peptide sequences, found that the formation of nanostructures or nanoaggregates stimulated via physiological means (pH, enzymes, and concentration) has commonly led to cell death, also among in vivo models.^6−13^ However, very little is known in terms of their mechanism of action and biochemical profile as the assembly of intracellular structures relies intricately on both supramolecular dynamics and cellular processes.

Nonetheless, from the biological features of β-amyloids, it is implied that the nanostructures plausibly exhibit broad bioactivity on a systemic level that impacts multiple pathways that ultimately leads to cell death. As such, we rationalized that an investigation into process families accompanied by precise chemical tools will enable a greater understanding to the cellular dynamics. Among these processes, metabolism is a critical facet that defines cellular life, and through it, the basic unit of energy in the form of adenosine triphosphate (ATP) is produced.^16^ Hence, metabolic interference that overwhelms the cell’s capability to adapt between glucose fermentation (glycolysis) and oxygen conversion oxidative phosphorylation (OxPhos) leads to the impairment of ATP-dependent pathways.^17,18^ In this regard, various supramolecular strategies have recently been developed and shown to be effective to target the accelerated metabolism of cancer.^19,20^ By coupling an in-depth metabolic study with a switchable self-assembling platform in living cells, we aim to establish a correlation of bioactive functions with superstructure formation.

**Figure 1:**
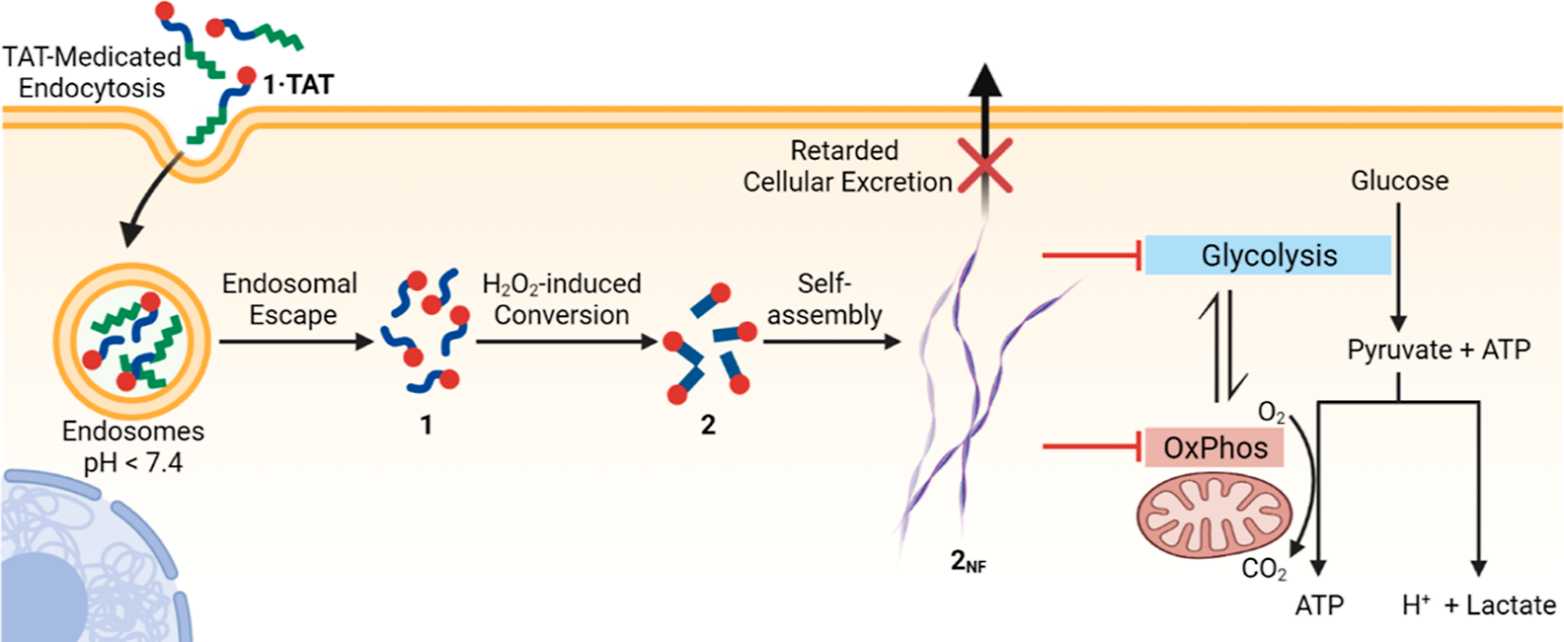
Schematic illustration of the intracellular H_2_O_2_-induced formation of linear platinum(II) complexes that self-assembles into platinum(II)-containing nanofibers, which affects energy homeostasis and disrupts cellular metabolism. Created with BioRender.com.

Herein, we present a platinum metallo-*iso-*tripeptide that undergoes a cascade of molecular and supramolecular transformations under specific intracellular environments to form near-infrared (NIR) emitting nanofibers (Figure 1). The isomerization of the *iso*-tripeptide forms the backbone, aligning the platinum(II) terpyridine (Pt-tpy) complex with its β-amyloid-like sequence, thus fostering the propagation of supramolecular order through π–π and metal–metal interactions. Pt complexes enable rich molecular topologies and diverse self-assembly structures in both the solid state and solutions.^21−25^ The synergy between platinum(II) complexes with other self-assembling motifs are well-known, often producing assemblies with emerging morphological and photoluminescence properties.^12,25−32^ By merging the features of platinum(II) complexes and peptides together with boronic acid-salicylhydroxamate (SHA) chemistry that responds to both physiological pH and reactive oxygen species,^11,33^ the progression from a dormant assembling precursor to the final superstructure can be controlled and imaged within cells. With the robust sequence of chemical transformation in place, the biological activity on aerobic glycolysis (AGlyc) and OxPhos is demonstrated on A549 lung cancer cells and MDA-MB-231 metastatic breast cancer cells. These cancer cell lines are known to be metabolically adaptive,^18,34^ thus enabling a deeper insight into the mechanism of action of the formed nanofiber. Additionally, pathways including histone deacetylase (HDAC) activity^35^ and actin restructuring^36,37^ that are dependent on ATP or metabolites will be assessed to evaluate the extent of the cellular impact.

## Results and Discussion

### Design and Synthesis of the Pro-Assembling Metallo-Isopeptide 1·TAT

**Figure 2:**
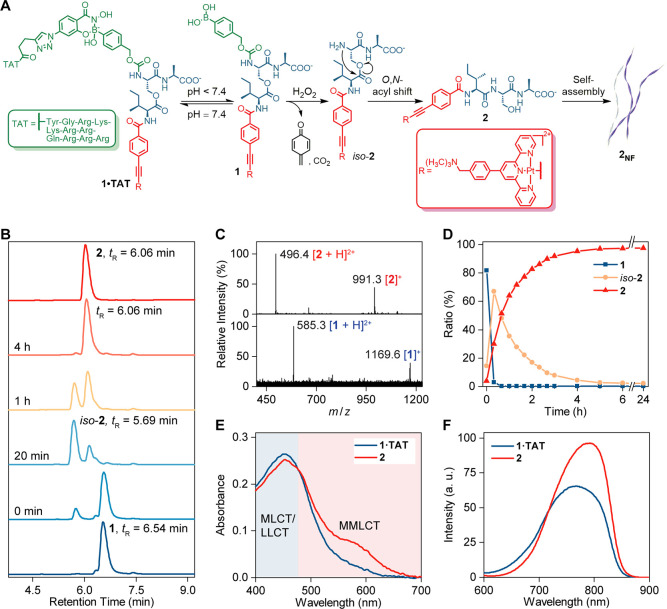
(A) Chemical design and reaction scheme for all chemical transformations of the ISA-platinum(II) complex **1·TAT** into nanofibers **2**_**NF**_. (B) LC–MS kinetic analysis over the H_2_O_2_ (0.5 mM)-induced linearization of **1** (50 μM) in a mixture of NH_4_HCO_3_ buffer (pH 7.4, 20 mM) and CH_3_OH (9/1, vol %). (C) Convoluted MS spectra in the LC–MS analysis for a reaction time of 0 min and *t*_R_ = 6.54–6.69 min (identified as complex **1**) and a reaction time of 4 h and *t*_R_ = 6.06–6.21 min (identified as complex **2**). (D) Molar ratio of **1**, *iso*-**2**, and **2** after addition of H_2_O_2_ based on the peak integration at 254 nm. (E) UV–vis absorption spectra of **1·TAT** and **2** (50 μM) in PB (pH 7.4, 50 mM). (F) Luminescence emission spectra of **1·TAT** and **2** (50 μM) in PB (pH 7.4, 50 mM). Excitation wavelength = 488 nm.

The metallo-*iso*tripeptide **1·TAT** is composed of three functionalities (Figure 2A): (1) a pro-assembling isopeptide (ISA) caged by an immolative boronic acid group,^11^ (2) a transporter peptide, trans-activator of transcription (TAT), that interacts with cellular membranes, enabling cellular uptake and endosomal release.^38^ This peptide carries a SHA group that binds to the boronic acid via a pH responsive dynamic covalent bond.^11,33^ (3) a Pt-tpy complex coordinated to the alkynyl group on the N-terminus of the *iso*-ISA peptide. The minimalistic design allows each peptide segment to perform their specified roles in a controlled, sequential manner. Hence, the expected mechanism of how the complex interacts with cells would first begin with the TAT-mediated endocytosis and intracellular endosomal escape of the complex.^11^ Once released to the near-neutral (pH 7.4) cytosol, endogenous H_2_O_2_ possesses sufficient oxidative strength to immolate the boronic acid cage generating the serine residue with a primary amino group. Next, an *O*,*N*-acyl shift occurs, causing the peptide to rapidly isomerize into the monomeric self-assembling peptide **2**, which assembles into the nanofibers **2**_**NF**_ (Figure 2A).

The synthesis of the Pt-*iso*tripeptide **1** and the **TAT-SHA** is performed separately using a combination of solution and solid-phase peptide syntheses (Figure S1). The complexation of the Pt-tpy unit to the alkynyl *iso*-ISA **S1** is performed in solution catalyzed by CuI, to afford the target Pt-*iso*tripeptide **1** characterized by one-dimensional/two-dimensional nuclear magnetic resonance (NMR) spectroscopy and liquid chromatography (LC)–mass spectrometry (MS) (Figures S8–S13). As a control and reference for subsequent mechanistic studies, the expected product **2** that will be formed at the end of the transformation cascade was also separately synthesized (Figures S14–S25). The dynamic covalent boronic acid-SHA bond between the Pt-*iso*tripeptide **1** and **TAT-SHA** was formed in PBS (pH 7.4) to yield the cell-penetrating **1·TAT**, characterized by matrix-assisted laser desorption ionization–MS (Figure S26)

### Formation of the Assembly Precursor Peptide 2 by Chemical Triggers

The first chemical transformation occurs under mildly acidic pH, where the boronic acid-SHA bond is cleaved to release **1** into the cytosol.^11^ The increase to near-neutral pH within the cytosol empowers the oxidation of the boronic acid by endogenous H_2_O_2_, resulting in its immolation, thus converting **1** to *iso*-**2** and initiating the rearrangement into **2**. The kinetics of the H_2_O_2_-triggered formation of **2** in solution was investigated by LC–MS (Figures 2B–D and S28). In aqueous solution, incubation with H_2_O_2_ for 20 min resulted in the complete disappearance of **1** and the emergence of two new peaks with retention times (*t*_*R*_) of 5.69 and 6.06 min, respectively (Figure 2B), both showing *m*/*z* values in agreement with the chemical formula of **2** (*m*/*z* = 496.4 for [**2** + H]^2+^ and 991.3 for [**2**]^+^) (Figure 2C and Figure S29). In comparison with **2** obtained via direct synthesis (*t*_R_ = 6.06 min), the former was assigned to be the intermediate formed (*iso-***2**) upon the immolation of the phenylboronic acid prior to isomerization. Further incubation with H_2_O_2_ led to an increase of **2**, with a 95% conversion at 4 h (Figure 2D). In contrast, less than 5% conversion was observed after 24 h incubation of **1** without H_2_O_2_ (Figure S30). Similar conversion kinetics is observed when **1** was incubated with H_2_O_2_ at cellular concentrations of 2.1 μM (Figures S31–S32 and S69), indicating that the intracellular oxidative stress is sufficient for initiating the conversion of **1**.

The square-planar platinum(II) center in the complexes endows the complex with optical properties associated with the nature of self-assembly. Complexes **1·TAT** and **2** displayed absorption bands centered at 460 nm in PB (Figure 2E), which can be assigned to a combination of dπ(Pt) → π*(tpy) metal-to-ligand charge-transfer (MLCT) and alkynyl-to-tpy ligand-to-ligand charge-transfer (LLCT) transitions. A lower energy absorption shoulder at ca. 570 nm is observed for both complexes, which can be attributed to a metal MLCT (MMLCT) transition.^25,39^ Complex **2** exhibited weaker MLCT/LLCT transition but markedly stronger MMLCT absorption than **1·TAT**. This indicates a higher extent of intermolecular *d*_z_^2^ interactions between platinum(II) centers in neighboring molecules of **2**, suggesting a proclivity for self-assembly in PB forming **2**_**NF**_.^25,39^ Both solutions showed NIR emission originating from the MMLCT excited states upon excitation at 488 nm (Figure 2F). Complex **1·TAT** exhibited emission centered at 770 nm, whereas complex **2** displayed red-shifted emission (λ_max_ = 792 nm) with a 1.5-fold higher intensity, which can be ascribed to the enhanced MMLCT transition as a result of self-assembly. The difference in the photophysical profile between the solutions of the complexes facilitates the tracking of their H_2_O_2_-induced conversion and subsequent assembly of **2** into **2**_**NF**_ in PB. Treatment of **1·TAT** with H_2_O_2_ (0.5 mM) led to a red shift in the λ_max_ with a concomitant increase in emission intensity (Figure S33). The conversion is completed in 280 min with the final emission profile resembling **2**_**NF**_, in agreement with the LC–MS study.

### Amyloid-Like Nanofibers 2_NF_ Form with High Molecular Order

**Figure 3:**
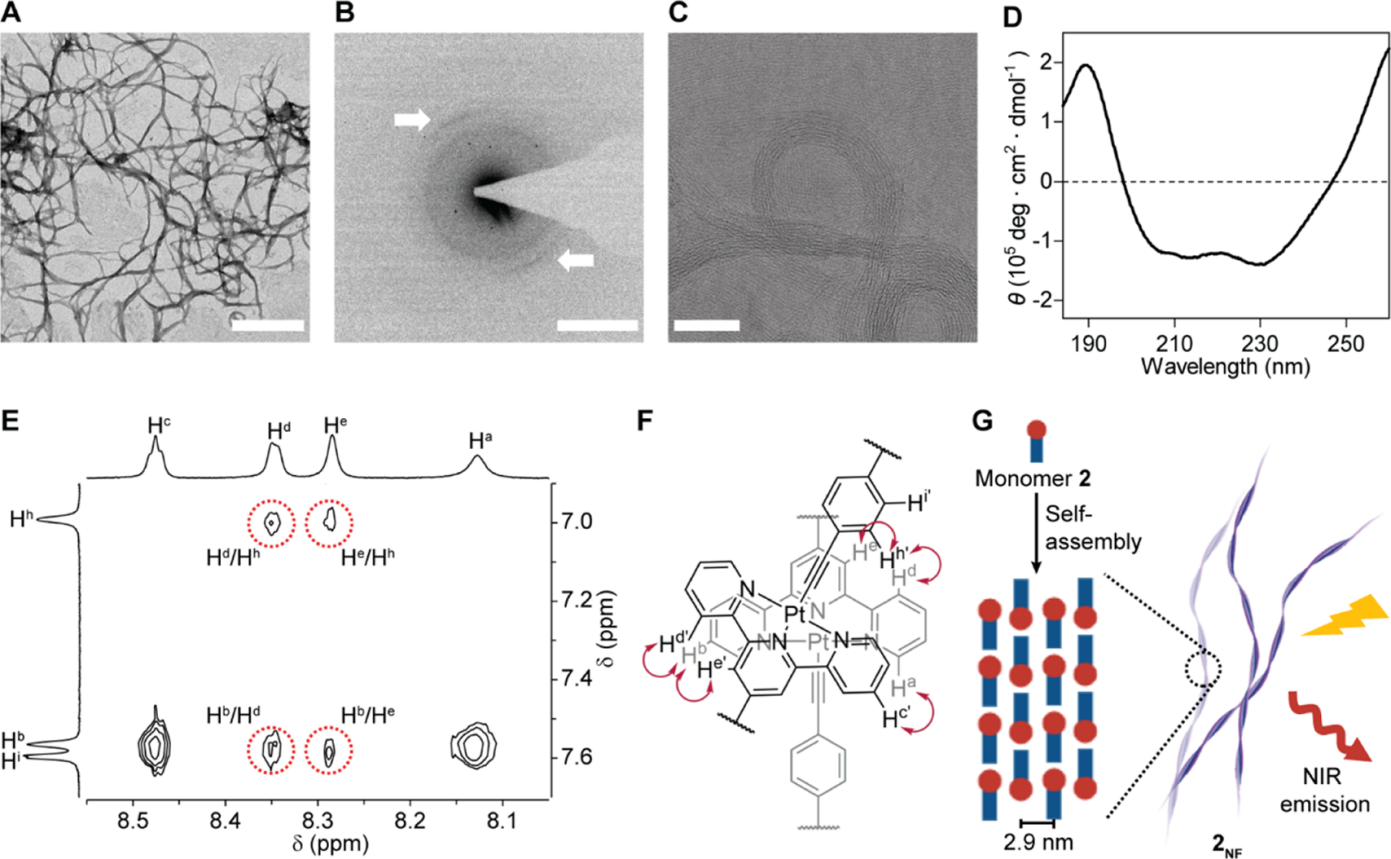
Nanofiber **2**_**NF**_ with a high degree of molecular order formed by self-assembly of **2**. (A) TEM image of the nanofibers **2**_**NF**_ (25 μM) in PB (pH 7.4, 10 mM). Scale bar, 200 nm. (B) SAED pattern of the nanofiber **2**_**NF**_ (100 μM) in H_2_O. The white arrows indicate diffraction arcs at 3.0 nm^–1^, that is, 3.3 Å. Scale bar, 3 nm^–1^. (C) Cryogenic high-resolution TEM image of the nanofiber **2**_**NF**_ (25 μM) in PB (pH 7.4, 50 mM). Scale bar, 50 nm. (D) CD spectra of **2**_**NF**_ in a mixture of PB (pH 7.4, 10 mM) and CH_3_CN (98/2, vol %). (E) Partial ^1^H, ^1^H NOESY NMR spectrum (850 MHz, 343 K) of **2** (1 mg/mL) in PB (pH 7.4, 50 mM). Intermolecular NOE cross-peaks are circled in the NMR spectrum. (F) Proposed molecular stacking mode of the Pt-tpy group in **2**_**NF**_ based on the NOESY NMR study. The red arrows indicate intermolecular NOE interactions between protons. (G) Proposed arrangement of monomer **2** in **2**_**NF**_ based on microscopy and NMR studies. The nanofibers showed a mean interfiber distance of 2.9 nm. Nanofiber formation also promotes NIR emission originated from the Pt-tpy groups in **2**_**NF**_. Intracellular formation of 2_NF_ induces actin restructuring.

The self-assembly of the complexes was visualized using transmission electron microscopy (TEM). Nanofibers were observed for **2**_**NF**_ at concentrations ranging from 5 to 100 μM (Figures 3A and S34), whereas **1** and **1·TAT** showed no defined nanostructures (Figure S35). Upon oxidation of **1·TAT** by H_2_O_2_, the transformation into **2** and subsequent self-assembly produced fibrillar morphology similar to the control (Figure S36). Selected-area electron diffraction (SAED) on **2**_**NF**_ showed diffraction arcs with a 3.3 Å lattice spacing, in agreement with the intermolecular distance of π–π interactions within the nanofibers.^40^ The diffraction arcs are perpendicular to the long axis of the fibers (Figures 3B and S37). Nanofiber formation is further supported by cryogenic high-resolution TEM studies on **2**_**NF**_ in PB, where the growth axis suggested an end-to-end molecular arrangement of the complexes (Figures 3C and S38). Each fiber was observed to be bundled with a mean interfiber distance of 2.9 nm.

Circular dichroism (CD) spectroscopy analysis on **2**_**NF**_ revealed a maximum at 190 nm corresponding to the π → π* of the carbonyl group, depicting H-bond interactions centered on the peptidic backbone. This band is coupled with the red-shifted n → π* transition visible at the 229 nm minimum.^41^ Exciton coupling parallel to the peptide backbone also exists for **2**_**NF**_ at 210 nm (Figure 3D).^41^ Additionally, a strong positive signal beyond 250 nm corresponding to the π → π* transition of the Pt-tpy group^25^ is also observed for **2**_**NF**_, demonstrating the importance of the Pt-tpy group in the self-assembly. In contrast, **1** only exhibited a minimum at 208 nm (Figure S39). The self-assembly behavior of **2**_**NF**_ at the molecular level was further examined using NMR. At 298 K, **2**_**NF**_ showed broad signals in the ^1^H NMR spectrum. Increasing the temperature led to sharpening of the signals accompanied by significant downfield shifts (Figure S41), which indicates self-assembly at 298 K. Nuclear Overhauser effect spectroscopy (NOESY) of **2**_**NF**_ revealed NOE cross-peaks between the protons on the tpy group and the phenyl ring of the alkynyl ligand (*H*^d^/*H*^h^ and *H*^e^/*H*^h^) and between the non-neighboring protons on the tpy moiety (*H*^a^/*H*^c^, *H*^b^/*H*^d^, and *H*^b^/*H*^e^) (Figures 3E and S47), implying that the Pt-tpy group adopts a twisted head-to-tail stacking upon self-assembly (Figure 3F), which is characteristic for Pt-tpy complexes.^39^ The microscopy and spectroscopy experiments suggest that **2**_**NF**_ possesses a high degree of molecular order, which is highly favorable for studies in a biological context because it enhances the proteolytic stability of the nanofibers and promotes NIR emission originated from the Pt-tpy group (Figure 3G).

**Figure 4:**
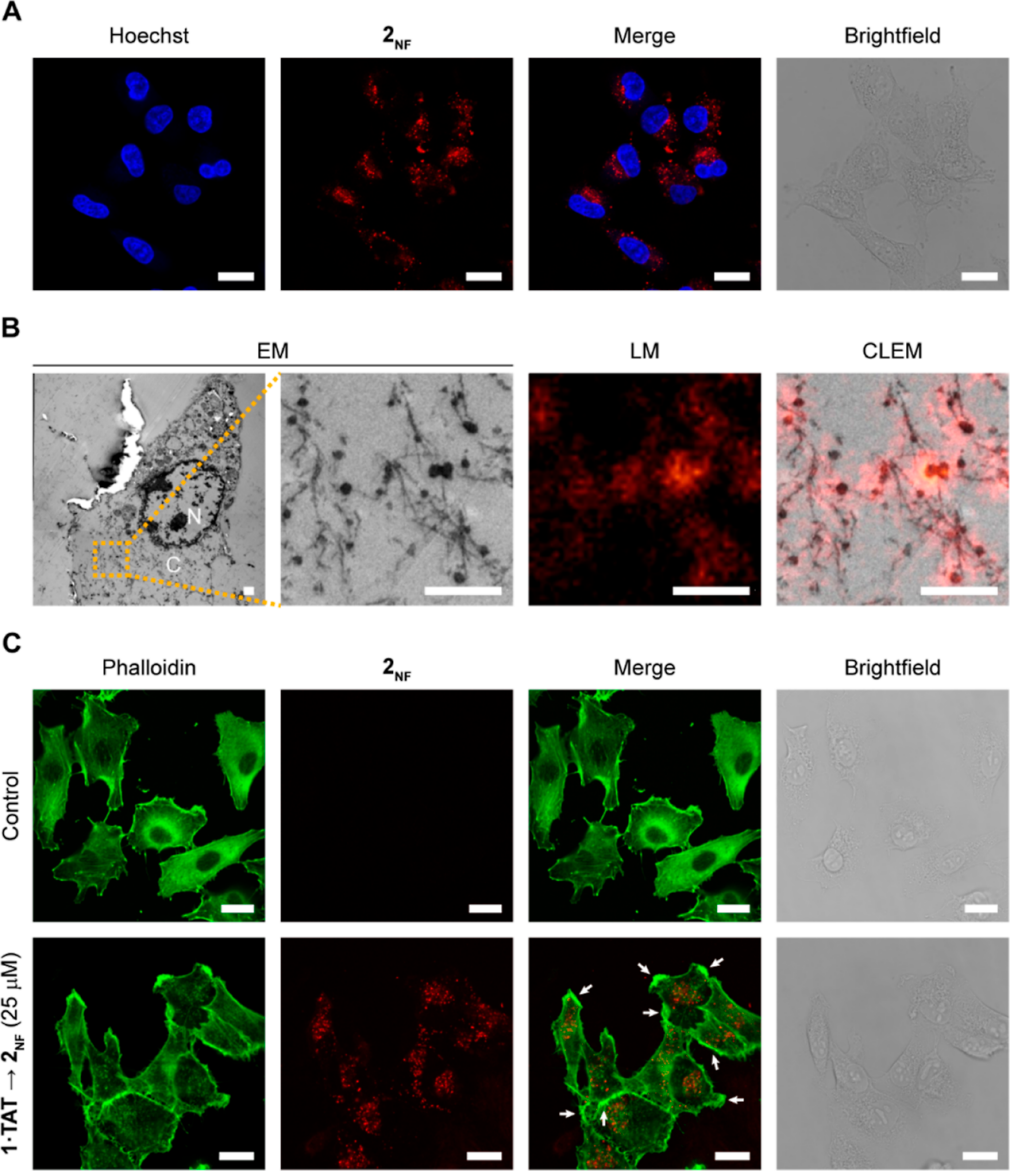
Internalization and assembly into nanofibers **1·TAT** → **2**_**NF**_ in the cytoplasm restructure actin filaments. (A) Confocal laser scanning micrographs of A549 cells treated for 4 h with **1·TAT** (25 μM) and Hoechst 33342 dye. Scale bar, 20 μm. (B) CLEM of A549 cells treated for 6 h with **1·TAT** (50 μM). N, nucleus. C, cytosol. Scale bar, 1 μm. (C) Confocal laser scanning micrographs of A549 cells treated for 4 h with **1·TAT** (25 μM) and Phalloidin-iFluor 405. White arrows indicate the fluorescent loci along the cell membrane. Scale bar, 20 μm. Nanofibers 2_NF_ disrupt AGlyc and OxPhos metabolic pathways.

The growth of the nanofibers **1·TAT**→**2**_**NF**_ was visualized in A549 lung alveolar adenocarcinoma cells and MDA-MB-231 metastatic breast cancer cells using confocal laser scanning microscopy (Figures 4A, S48, and S49). Within 4 h, TAT-dependent internalization was observed (Figure 4A). Assisted by the NIR emission of the platinum(II) center, correlative light-electron microscopy (CLEM) observed that newly formed luminescent nanofibers within the cytoplasm occur after escaping from the endosomal vesicles (Figures 4B, S50, and S51). In line with the pH dependence of endogenous H_2_O_2_ activity on the boronic acid, the TEM micrographs revealed that the entry into the near-neutral (pH 7.4) cytosol from the acidic (pH 6.0) endosomes initiates the nanofiber formation. At the onset of nanofiber growth, the first metabolic consequence of newly formed **2**_**NF**_ can be detected through early changes in the cytoskeleton, whose formation is tightly coupled to glycolytic pathways.^36,37^ An investigation using cytoskeleton stain Phalloidin-iFluor 405 demonstrated a restructuring of actin filaments toward the cellular membrane (Figures 4C and S54). Using both 10 and 25 μM, the appearance of fluorescent loci along the cell membrane was only observed at 25 μM (Figure 4C, lower panel), suggesting that a critical concentration threshold of **2**_**NF**_ has to be reached for actin restructuring. Actin filaments in cells are regulated by Rho-GTPases,^42^ which are accumulated on the lateral membranes in cultured mammalian epithelial cells.^43^ Disruption can occur at the Rho-GTPase level or by the disassembly of F-actin, each leading to different observations through phalloidin stain. F-actin fragmentation generates phalloidin signals aggregating around the remnants of the actin filaments.^44^ In contrast, interference toward Rho-GTPases accumulates phalloidin signals to the membrane because F-actin cannot propagate without a functioning Rho-GTPase or its substrate, GTP.^45^ As the equilibrium of GTP and ATP is typically maintained by nucleoside-diphosphate kinases,^46^ we hypothesize that broader, systemic effects may have been the origin of the observations since the nanofibers are unlikely to inhibit specific enzymes.

**Figure 5:**
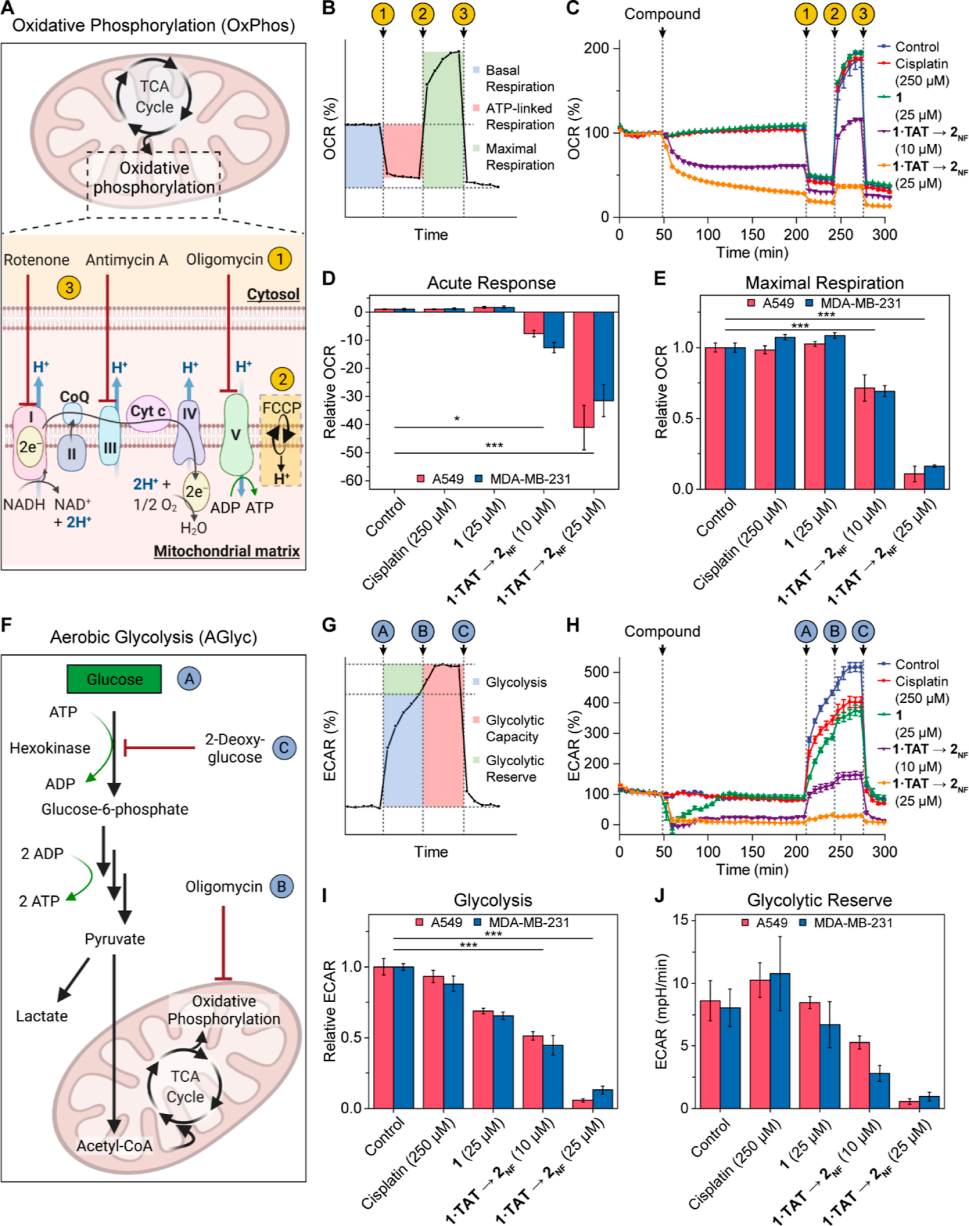
Transformation **1·TAT** → **2**_**NF**_ disrupts both AGlyc and OxPhos metabolism of A549 and MDA-MB-231 cells. (A) Scheme of a mitochondrion with focus on the ETC, the site of OxPhos. The compounds of the Mito stress test modulate the ETC when added in the indicated injection sequence. (B) Profile of the OCR during the Mito stress test. Numbers represent the compounds of the Mito stress test shown in Figure 5A. (C) Effect of **2**_**NF**_ on the OCR of MDA-MB-231. The last measurement before treatment injection is set as 100%. (D,E) Acute response (D) and maximal respiration (E) of A549 and MDA-MB-231 cells. (F) Schematic representation of glycolysis and cell respiration, which are affected by the compounds of the glycolysis stress test. Each target of the modulators of the glycolysis and OxPhos used in the assay is indicated with respect to the injection sequence. (G) Profile of the ECAR during the glycolysis stress test. Characters represent the compounds of the glycolysis stress test shown in Figure 5F. (H) Effect of **2**_**NF**_ on the ECAR of MDA-MB-231. (I,J) Effect of **2**_**NF**_ on the glycolysis (I) and glycolytic reserve (J) of A549 and MDA-MB-231 cells. For (D,E,I), the data are expressed as relative values to untreated control cells. Data are presented as mean ± s.e.m., *n* ≥ 5. Statistical significance was calculated by ANOVA with a Tukey post hoc test. *p < 0.05, **p < 0.01, ***p < 0.001.

We investigate the impact of **2**_**NF**_ on the main mechanisms of cellular metabolism, AGlyc and OxPhos pathways, which are core upstream processes involving at least 10% of the human proteome.^47^ The consumption of oxygen to fuel mainly OxPhos, and the extracellular acidification as a function of AGlyc were analyzed in A549 and MDA-MB-231 cells using a Seahorse extracellular flux analyzer (Figure 5). Treatment of both cell lines with **1·TAT** that transforms into **2**_**NF**_ demonstrated a reduction in the oxygen consumption rate (OCR) within 4 h (Figure 5C), with 25 μM inducing 41-fold and a 32-fold decrease, respectively, in A549 and MDA-MB-231 cells (Figure 5D). The OCR, expressed as pmol/min, is a first measure of OxPhos (Figure 5A–C) activity, which takes place primarily in the mitochondria.

Key parameters of mitochondrial respiration were investigated to unravel the impact of **2**_**NF**_ on OxPhos by the sequential use of modulators of the electron transport chain (ETC): rotenone (Complex I), antimycin A (Complex III), oligomycin (ATP synthase), and FCCP (carbonyl cyanide 4-(trifluoromethoxy)phenylhydrazone) to disrupt the mitochondrial membrane potential.^48,49^ Treated cells exhibit less than 10% of the FCCP-induced maximal respiration compared to untreated cells, highlighting the inability of cells to sustain energy demand even when stimulated to operate at maximum capacity (Figure 5E). Thus, in addition to the impact on OxPhos depicted by the acute response, **2**_**NF**_ induces a massive and persistent effect on mitochondrial respiration that prevents cells from adapting to stress. This phenomenon is further confirmed by a reduction of most of the OxPhos-related parameters, such as ATP production and spare respiratory capacity, which is a potential indicator of mitochondrial damage (Figures S58–60). Based on the burden of **1·TAT** → **2**_**NF**_ on mitochondrial respiration, we subsequently evaluated parameters related to AGlyc where cancer cell types are known for being able to rewire their cell metabolism between OxPhos and AGlyc, upregulating accordingly one of the two metabolic pathways to satisfy their energetic needs.^18,34^

Therefore, the AGlyc of A549 and MDA-MB-231 cells was measured using their extracellular acidification rate (ECAR_glyc_), expressed as mpH/min (Figure 5F–H). Extracellular acidification occurs when glucose is converted to pyruvate and lactate, accompanied by the extrusion of protons into extracellular space^50^ (Figure 5F). The cells first underwent glucose starvation to account for the basal non-glycolytic acidification, followed by the addition of saturating amounts of glucose to trigger glycolysis. The formation of the nanofibers at 25 μM shuts down glycolysis of A549 and MDA-MB-231 to 6 ± 1 and 13 ± 3%, respectively (Figure 5I). The capability of cells rewiring their metabolism^51^ was demonstrated by adding oligomycin, the ATP synthase inhibitor,^48^ to prevent OxPhos and thereby inducing the switch to glycolysis. In the untreated control cells, the incubation with oligomycin raised the glycolytic activity by +8.6 ± 1.6 mpH/min (A549) as well as +8.0 ± 1.5 mpH/min (MDA-MB-231) (Figure 5J). This adaptation is termed the glycolytic reserve, which is activated upon mitochondrial stress/dysfunction. On the contrary, inducing the metabolic switch in cells where **2**_**NF**_ I is formed only increases the glycolytic acidification by only 0.6 ± 0.2 mpH/min for A549 cells and 0.9 ± 0.4 mpH/min for MDA-MB-231 cells (Figure 5J). Hence, even under severe stress conditions, aerobic glycolysis remained low, suggesting that the capabilities of the cells to compensate for this disruption were limited. To further ascertain that the extracellular acidification is the consequence of glucose metabolism, 2-deoxy-glucose, a competitive inhibitor toward glucose hexokinase,^48^ was added. By inhibiting the first enzyme of glycolysis and thus the suppression of ECAR, we demonstrated that the observed extracellular acidification is an accurate measure of their glycolytic activities. In summary, the transformation of **1·TAT** → **2**_**NF**_ has shown the ability to impair cancer cell fitness by interfering with both OxPhos and AGlyc and preventing metabolic adaptation, one of the most prominent features of cancer cells to react and address stress stimuli.^52^

### Nanofibers 2_NF_ Induce Early Apoptosis

**Figure 6:**
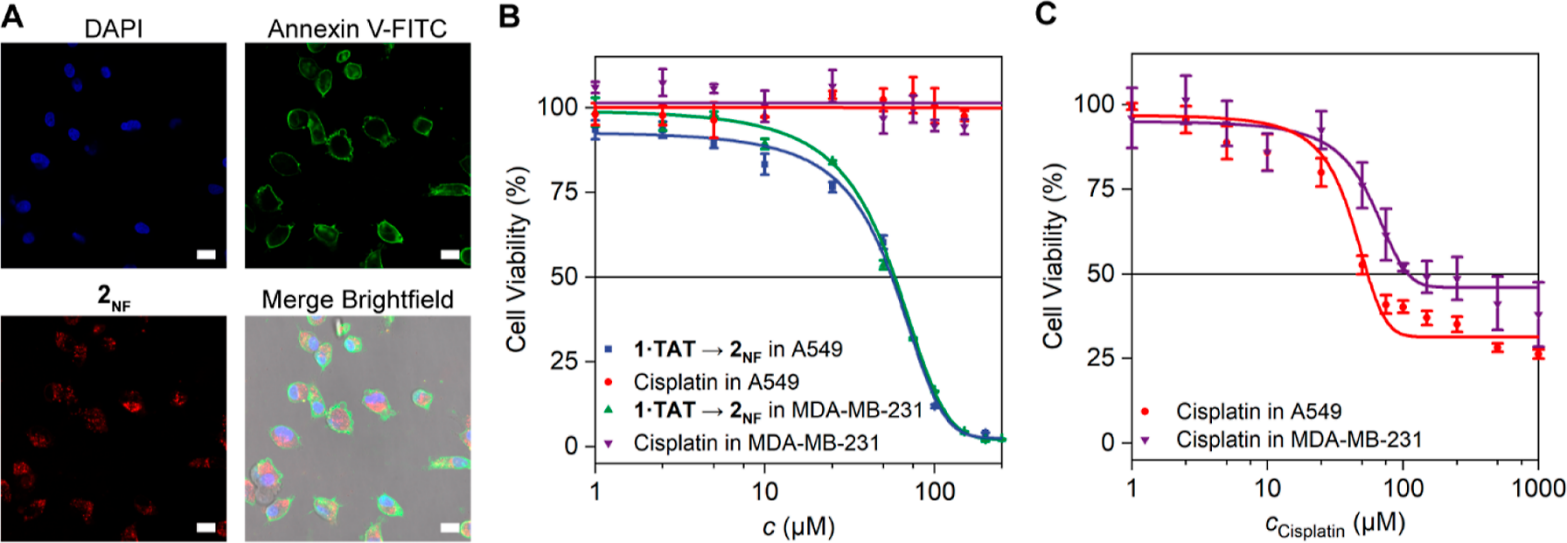
Toxicity of **1·TAT** → **2**_**NF**_ toward A549 and MDA-MB-231 cells. (A) Apoptosis was investigated for A549 cells using Annexin V assay. Cells were incubated 4 h with **1·TAT** (red) and treated with Annexin V (green) afterward. Nuclei were stained using DAPI (blue). Annexin V binds to the cell membrane upon an inversion of phosphatidylserine motifs toward the extracellular space, indicating apoptosis. Scale bar, 50 μm. (B) Cell viability assay of A549 and MDA-MB-231 cells treated with **1·TAT** and cisplatin for 4 h, revealing viable cells after cisplatin treatment but significantly reduced cell viability after transformation into **2**_**NF**_. (C) Cell viability assay of cisplatin at 24 h. The cell viability was determined using the CellTiter-Glo Luminescence Cell Viability Assay Kit.

With the formation of **2**_**NF**_ disrupting both AGlyc and OxPhos processes, the production of metabolites such as glucose-6-phosphate (G6P) is investigated. Hence, G6P-related pathways such as the downstream production of NADPH by G6P dehydrogenase will be impeded, leading to the downregulation of HDAC activity.^35^ Indeed, treated A549 and MDA-MB-231 cells showed concentration dependent reduction in HDAC activity at 4 h, with 25 μM showing a 25 and 10% decrease, respectively (Figure S66). With the impairment of metabolic pathways and HDAC activity, apoptosis of the cells was evaluated using Annexin V-FITC and cell viability assays. At 25 μM, **2**_**NF**_ induced the presence of phosphatidylserine on the external leaflet of the cell membrane, which was detected by the binding of Annexin V-FITC. This characteristic observation of early apoptosis suggests that the population of cells were undergoing the hallmark changes associated with programmed cell death (Figures 6A and S67).^53^ Importantly, the formation of **2**_**NF**_ is effective at 4 h in both A549 cells and MDA-MB-231 cells, whereas cisplatin is ineffective at this timeframe. Even with 24 h treatment of cisplatin at 1 mM, >25% of the cells survived, demonstrating, at first glance, the mechanistical difference between small molecules and nanostructures in terms of cellular impact (Figure 6B,C).^54^ The disparity in time-dependent efficacies of cisplatin and **2**_**NF**_ is likewise apparent in the metabolic studies, where a 4 h cisplatin treatment did not produce observable effects on AGlyc and OxPhos even at a 10-fold higher (250 μM) concentration (Figure 5). It is interesting to note that even though the formation of nanofibers demonstrates a more significant impact on oxygen-dependent ATP production on A549 cells compared to MDA-MB-231 cells, the IC_50_ values of **1·TAT** → **2**_**NF**_ on both cell lines (60.6 μM/A549; 58.5 μM/MDA-MB-231) are not significantly different. We hypothesize that upon the critical assembly concentration of the nanofibers, the damage threshold toward cellular metabolism has reached a point of no return. At this stage, any further damage on cells that are already undergoing apoptosis would not provide additional statistical weight on the overall cell viability. In comparison, **1·TAT** → **2**_**NF**_ showed similar cytotoxicity toward non-cancerous CHO and HEK cell lines (IC_50_ = 49.8 and 72.3 μM against CHO and HEK cells, respectively, Figure S68) compared to cancerous cells lines because a similar H_2_O_2_ concentration was detected (Figure S69). With these results, we highlight the broad system-level impact that is cell line independent as these structures affect the fundamental pathways of cell vitality.

## Conclusions

In conclusion, we have designed a pro-assembling Pt(II) metallo-peptide that undergoes a step-wise transformation into the NIR emitting nanofibers within A549 and MDA-MB-231 cells. The Pt-tpy complex directs the supramolecular order and directionality of the packing within the fiber axis, while providing NIR photoluminescence. The formation of the nanofibers rapidly damages energy homeostasis and essential metabolic pathways AGlyc and OxPhos, preventing the cells from mounting adaptive strategies that are known, particularly in cancer, to resist specific small molecule inhibitors, increasing metastasis. Pathways that are intrinsically linked to ATP production (cytoskeleton) and glucose metabolites (HDAC) are impaired, confirming the mechanistic origin of the formed **2**_**NF**_ nanofiber. Rapid apoptosis is induced within 4 h compared to cisplatin and found to leverage a similar potency on both cell types. Collectively, assembly-driven strategies to design metabolically active materials within cancer cells can be exploited to induce systemic level effects and compensate limitations of small molecules and biologics. By demonstrating that complex cellular functions can be addressed purely by nanostructure formation through controlled cascade reactions and self-assembly, we showcase a new avenue to address grand challenges in drug discovery and synthetic biology.

## Supporting Information Available

Details of instruments, synthesis and characterization of the complexes, and experimental procedures for cellular assays (PDF)
