# Supplementary material for: In Situ Assembly of Platinum(II)-Metallopeptide Nanostructures Disrupts Energy Homeostasis and Cellular Metabolism
Source: J Am Chem Soc. 2022 Jun 22;144(27):12219–28. doi: 10.1021/jacs.2c03215 (PMC9284552; doi:10.1021/jacs.2c03215)
Supplement: Supplementary file 1 — ja2c03215_si_001.pdf [file ja2c03215_si_001.pdf]

SUPPLEMENTARY INFORMATION

In Situ Assembly of Platinum (II)-Metallopeptide Nanostructures Disrupts Energy Homeostasis and Cellular Metabolism

Zhixuan Zhou,[1] Konrad Maxeiner,[1] Pierpaolo Moscariello,[1] Siyuan Xiang,[1] Yingke Wu,[1] Yong Ren,[1] Colette J. Whitfield,[1] Lujuan Xu,[1] Anke Kaltbeitzel,[1] Shen Han,[1] David Mücke,[2] Haoyuan Qi,[2,3] Manfred Wagner,[1] Ute Kaiser,[2] Katharina Landfester,[1] Ingo Lieberwirth,[1] David Y.W. Ng,[1,*] Tanja Weil[1,*]

[1] Max Planck Institute for Polymer Research, 55128 Mainz, Germany.

[2] Central Facility of Materials Science Electron Microscopy, Universität Ulm, 89081 Ulm, Germany.

[3] Faculty of Chemistry and Food Chemistry & Center for Advancing Electronics Dresden (cfaed), Technische Universität Dresden, 01062 Dresden, Germany.

* Corresponding Author: David Y. W. Ng, david.ng@mpip-mainz.mpg.de

* Corresponding Author: Tanja Weil, weil@mpip-mainz.mpg.de

Table of Contents

1 Supplementary methods 3
1.1 Materials 3
1.2 Synthesis of the complexes 3
1.3 Self-assembly formation of $\mathbf{2}_{\mathbf{NF}}$ 3
1.4 Spectroscopy analysis 3
1.5 HPLC-MS analysis of H_2O_2-induced conversion 4
1.6 TEM analysis 4
1.7 SAED Analysis 4
1.8 Cryo-TEM analysis 4
1.9 ^1H, ^1H NOESY NMR experiments 5
1.10 Cell culture 5
1.11 Cellular uptake studies 5
1.12 CLEM studies 5
1.13 Cell metabolism assay 6
1.14 Histone deacetylase (HDAC) activity assay 7
1.15 Annexin V staining assay 7
1.16 Cell viability assay 7
1.17 Determination of intracellular H_2O_2 concentration 8
2. Synthesis and characterization details 8
2.1 Instruments 8
2.2 Reaction scheme for the synthesis of the compounds 10
2.3 Synthesis of compound **S1** 11
2.4 Synthesis of compound **1** 15
2.5 Synthesis of compound **S2** 18
2.6 Synthesis of compound **2** 22
3 H_2O_2 cascade transformation 25
4 Self-assembly profiles of $\mathbf{2}_{\mathbf{NF}}$. 29
5 NMR analysis on the self-assembly profile of $\mathbf{2}_{\mathbf{NF}}$. 32
6 Cellular uptake and intracellular self-assembly 37
7 Metabolic inhibitions by the **1·TAT**→$\mathbf{2}_{\mathbf{NF}}$ 41
8 Cytotoxicity of **1·TAT**→$\mathbf{2}_{\mathbf{NF}}$ 47
9 References 49

1 Supplementary methods

1.1 Materials

Reagents and solvents were purchased from commercial sources and were used without further purification. Peptide Synthesis grade reagents were used for synthesizing the peptides. HPLC was performed using CH_3CN in HPLC grade and H_2O for HPLC and reactions was obtained from a Millipore purification system.

1.2 Synthesis of the complexes

Solid-phase peptide synthesis was conducted to prepare the alkynyl-functionalized peptidic precursors. Dehydrohalogenation reaction between the alkynyl group and a chloroplatinum (II) complex under an inert atmosphere led to the coordination of the alkynyl ligand to the Pt-tpy moiety to afford **1**, the precursor for **1·TAT** with a free boronic acid group. Linear conjugate **2** was synthesized, as a control, using a similar strategy. SHA-functionalized TAT peptide motif (**SHA-TAT**) was synthesized using a reported method.[2] Complexation of **1** and **SHA-TAT** via SHA-boronic acid dynamic covalent bond was achieved by mixing them in a 1:1 molar ratio in a mixture of DMSO and phosphate buffer (PB, pH 7.4, 50 mM) (DMSO/PB, 2/98, vol%), leading to the formation of **1·TAT**. The compounds were identified by NMR spectroscopy, high-performance liquid chromatography, and mass spectrometry. A detailed procedure for the synthesis and characterization of the complexes can be found in Section S2.

1.3 Self-assembly formation of $\mathbf{2_{NF}}$

Monomeric complex **2** was dissolved in DMSO at a high concentration (10 mM) before diluted to different concentrations using PB (pH 7.4, 50 mM). Self-assembly was induced by agitating the solution in an Eppendorf Thermomixer for 2 h at 37 °C at 500 r.p.m.

1.4 Spectroscopy analysis

The UV/Vis absorption spectra for the solutions of the compounds were recorded on a Thermo Scientific™ NanoDrop 2000/2000c spectrophotometer in a Hellma high precision quartz cell, 10 × 10 mm light path. The luminescence emission spectra for the solutions of the compounds were recorded on a Cary Eclipse fluorescence/luminescence spectrophotometer in a Hellma high precision quartz cell, 10 × 10 mm light path. For tracking of H_2O_2-induced conversion from **1** to $\mathbf{2_{NF}}$ by luminescence, Compound **1** was dissolved in DMSO at a high concentration (10 mM) before diluted to desired concentrations using PB (pH 7.4, 50 mM) containing H_2O_2 (0.5 mM). The luminesce emission profile of the mixture was recorded over 360 min with 10 min intervals. CD spectra were recorded on a JASCO J-1500 spectrometer in a Hellma high precision cell with 1 mm light path.

1.5 HPLC-MS analysis of H_2O_2-induced conversion

Complex **1** was dissolved in a mixture of NH_4HCO_3 buffer (pH 7.4, 20 mM) and CH_3OH (90/10, vol%) at a concentration of 100 μM. Equal volume of H_2O_2 (1 mM) solution in a mixture of NH_4HCO_3 buffer (pH 7.4, 20 mM) and CH_3OH (90/10, vol%) was added to initiate the conversion. HPLC-MS analysis of the mixture was performed on a Shimadzu LC-MS 2020 equipped with a Kinetex EVO C18 column (50 × 2.1 mm, 2.6 μm, 100 Å), an SPD-20A UV-Vis detector, and an electrospray ionization source. MilliQ water, acidified with 0.1% formic acid, and CH_3CN were used as solvents for all measurements. The solvent gradient started with 5% CH_3CN and 95% water. This solvent ratio was kept constant for 2 min, and then the CH_3CN content was linearly increased to 95% over 14 min. The molar ratio of the compounds was calculated using peak areas at 254 nm. For analysis of lower concentration of complex **1** (final concentration of 2.0 μM) and H_2O_2 (final concentration of 2.1 μM), the solution mixture was prepared using the same protocol and the conversion was investigated using a ZORBAX Eclipse XDB-C18 HPLC column (9.4 × 250 mm, 5 μm).

1.6 TEM analysis

TEM samples were prepared by dropping a solution of $\mathbf{2_{NF}}$ onto a carbon-coated copper grid. After 5 min, the excess solution was removed by a filter paper. The sample was then stained inverting the TEM grid (sample side down) on a droplet (10 μL) of 2% uranyl acetate solution for 30 seconds. After removing the staining solution with a filter paper, the sample was washed with Milli-Q water for three times (shaken in water for 6 seconds for each time). After drying in air, the measurement was conducted on a JEOL JEM-1400 TEM operating at an accelerating voltage of 120 kV.

1.7 SAED Analysis

Sample for SAED analysis was prepared by drop-casting a solution of $\mathbf{2_{NF}}$ in H_2O on an ultrathin carbon grid. SAED analysis was performed on an image-side aberration-corrected FEI Titan 80-300 operated at 300 kV. The microscope is equipped with a CEOS hexapole aberration-corrector which corrects the geometrical axial aberrations up to the 3rd-order. Diffraction data acquisition was conducted on a Gatan UltraScan CCD camera. For SAED acquisition, we used a selected-area aperture with a physical diameter of 50 μm, corresponding to a diameter of 700 nm in the image plane. In order to avoid substantial electron radiation damage on the specimen, a low-dose technique has been applied. The total electron dose for SAED acquisition was 0.8 e-/$Å^2$ (dose rate: 0.04 e-/$Å^2$s, acquisition time: 20 s).

1.8 Cryo-TEM analysis

For cryo-TEM examinations the sample was prepared using a Mark V Vitrobot ® (Thermo Fisher Scientific) cryo plunge device. Samples were plunged from a climate chamber operated at room temperature with 100 % relative humidity. The sample dispersion was applied either to a Quantifoil ® or lacey holey carbon grid and plunged into liquid ethane. Subsequently, the specimen grid was transferred to a Titan Krios G4 TEM (Thermo Fisher Scientific). Images were acquired using a Gatan K3 direct detection camera.

1.9 ^{1}H, ^{1}H NOESY NMR experiments

The NOESY NMR measurements were performed with a 5 mm QXI ^{1}H/^{13}C/^{15}N/^{31}P probe equipped with a z-gradient on the 850 MHz Bruker AVANCE III system. The spectroscopic widths of the ^{1}H, ^{1}H NOESY experiments were 13500 Hz (850 MHz; 16 ppm) in both dimension (f1 and f2) and the relaxation delay is 2 s. The mixing time was kept at 300ms. A detailed analysis for the NOESY NMR experiments can be found in Section S5.

1.10 Cell culture

A549 and MDA-MB-231 cells were cultured at 37 °C and 5% CO_2 in Dulbecco's Modified Eagle's Medium (DMEM, high glucose), supplemented with 10% FBS. Both cell lines were cultured in T75 culture flask and subcultivated two to three times per week.

1.11 Cellular uptake studies

A549 and MDA-MB-231 cells were seeded at a density of 25,000 cells/well in an 8-well confocal plate. After adhering for 24 h, cells were treated with the sample for 4 h at 37 °C. Before adding to the cells, samples were pre-incubated to form the **1·TAT** by dissolving **1** in DMSO and mixing with an equimolar amount of the TAT peptide, which was dissolved in Dulbecco's PBS. The sample was further diluted with DPBS to a final volume of 40 µL (DMSO/DPBS, 10/90, vol%). Sample solutions were further diluted 1:4 with DMEM and added to the cells (total DMSO content = 2%), after removing the existing medium from the wells. After the incubation time was over, the cell nucleus was stained with Hoechst 33342 dye for 20 min at 37 °C. A459 cells were additionally treated with Phalloidin-iFluor^{TM} 405 instead of the Hoechst 33342 dye to stain the cytoskeleton. The staining solution was removed, and fresh FluoroBrite DMEM Medium was added to the cells before they were imaged by confocal laser scanning microscopy. Confocal images of cells were taken on a Leica TCS SP5 and Visitron Spinning Disc microscope. To monitor the Hoechst, DAPI and Phalloidin dyes, a 405 nm excitation diode laser was used with an emission filter from 415-500 nm. For Annexin-FITC, an Argon laser was used for excitation at 488 nm with an emission filter from 500-600 nm. To study the uptake of the compound **1·TAT** and subsequent formation of $\mathbf{2}_{\mathbf{NF}}$, a DPSS laser was used at 561 nm excitation and an emission range from 600-800 nm.

1.12 CLEM studies

One day before treatment, cells were seeded and grown on carbon pre-coated sapphire disks (3 mm; M. Wohlwend GmbH), which were sterilized and oxygen plasma cleaned before use (30 s, 0.2 mbar, 20% power; Femto, diener electronic). The disks were placed in a 24-well plate with 50,000 cells/well, which was incubated at 37°C, 5% CO_2 overnight. After incubation with **1·TAT** (50 µM, 6h), each sapphire disk was collected and slightly immersed into 1-hexadecene before placing them between two aluminum plates (3 mm, Plano). The aluminum plates with the sample were placed into a specimen holder for high pressure freezing in a Wohlwend HPF Compact 01 high-pressure freezer with a pressure of 2100 bar for 2–3 s. The specimen holder was withdrawn from the freezer and immersed into liquid nitrogen to release the sample. The frozen sample was then stored in a container filled with liquid nitrogen. Subsequently, freeze substitution of the sample was carried out in a 0.5 mL Eppendorf tube using an AFS2 freeze substitution unit (Leica). Each tube contained freeze

substitution solution, consisting of 0.2wt/vol% osmium tetroxide, 0.1wt/vol% uranyl acetate, and 5% distilled water in acetone. The tubes were firstly kept at –90 °C and automatically warmed up to 0 °C in 24 h. After keeping at room temperature for 1 h, the substitution solution was removed, and the samples were washed three times with acetone. Each sample was infiltrated in an ascending epoxy resin series (30%, 50%, and 75% in acetone) for 1 h before final infiltration in 100% epoxy resin overnight. Subsequently, each sample was transferred into a new Eppendorf tube containing freshly prepared pure epoxy resin for polymerization at 60 °C for 72 h. After polymerization, sample blocks were kept at room temperature until their sectioning. Sample blocks for each time point were trimmed and sectioned into 100 nm sections by a 35º diamond knife (Diatome) in EM UC6 ultramicrotome (Leica). Sections were then carefully placed onto H6 copper finder grids (Plano) and imaged on a confocal light microscope (Leica SP5) using 561 nm excitation and a 610/60 ET bandpass. For later registration to TEM images, transmitted light and reflected light images were simultaneously recorded. Electron microscopy images were collected on a Jeol 1400 120kV using a 2K bottom mount CCD camera and a Tecnai F20 200 kV TEM by FEI using a K2 Gatan camera. The TEM images were stitched to show the entire cell. Registration of light and electron microscopy images was done by manual landmark registration on entire cells using ImageJ. Landmark selection was based on features identified in the reflected light and transmitted light images.

1.13 Cell metabolism assay

The effect of the compounds on the metabolism of A549 and MDA-MB-231 cells was investigated using the Seahorse XFe96 Analyzer (Agilent Technologies). Seahorse XF Glycolysis Stress Test Kit and Seahorse XF Cell Mito Stress Test Kit were used following the manufacture's protocol to determine the glycolytic and respiratory activity of the cells. Cells were seeded one day before the assay and incubated overnight at 37°C, 5% CO_2 in DMEM, high glucose supplemented with 10% FBS. The cartridge for sample loading was loaded with calibration solution (200 µL per well) and incubated at 37°C in a non-CO_2 incubator. On the day of the assay, Seahorse XF DMEM, pH 7.4 was supplemented with glutamine, glucose and pyruvate regarding to the protocols for the specific assay kits. For the Mito Stress Assay the XF DMEM contained pyruvate (1 mM), glutamine (2 mM), and glucose (10 mM). For the Glycolysis Stress Assay the XF DMEM contained 2 mM glutamine. Cells were washed once with XF DMEM (200 µL) and the medium was substituted with fresh XF DMEM (180 µL) and placed in a 37°C non-CO_2 incubator for 45 to 60 minutes. The cartridge was prepared by loading the samples (20 µL) in Port A of each well and the components of the performed assay in Ports B-D. The cartridge and 96-well plate with the cells were loaded in the instrument and the content of the ports was injected sequentially according to the specific assay. The pH, to monitor the extracellular acidification rate (ECAR) and the oxygen concentration, to monitor the oxygen consumption rate (OCR) were constantly measured throughout the entire time of the assay. Both, Glycolysis Stress Assay and Mito Stress Assay were performed in two independent experiments with n=4. Bar graphs are shown for the mean ± standard error of the mean (s.e.m.) for raw and processed data. For the processed data, all values were set to 100% OCR/ECAR for the last measurement prior to sample addition before calculating the results. Additionally, the values were normalized to the control for each independent experiment, before calculating the mean value and SEM. Statistical significance was calculated by ANOVA with a Tukey post hoc test and was defined as $*p < 0.05$, $**p < 0.01$, $***p < 0.001$.

For the Mito Stress Assay, Ports B-D were loaded with Oligomycin (22 µL), carbonyl cyanide-4 (trifluoromethoxy) phenylhydrazone (FCCP) (25 µL) and a mixture of Rotenone and Antimycin A(Rot/AA) (27 µL). After loading the cell plate eight measurement cycles were performed as baseline control. After addition of the compound, 25 measurement cycles were performed before the components of the Mito Stress Assay were added. For each component five measurement cycles were performed. These cycles resulted in an overall incubation time of the cells with the compounds of 4 h.

For the Glycolysis Stress Assay, Ports B-D were loaded with glucose (22 µL), oligomycin (25 µL) and 2-Deoxyglucose (27 µL). After loading the cell plate eight measurement cycles were performed as baseline control. After addition of the compound 25 measurement cycles were performed before the components of the Glycolysis Stress Assay were added. For each component five measurement cycles were performed. These cycles resulted in an overall incubation time of the cells with the compounds of 4 h.

1.14 Histone deacetylase (HDAC) activity assay

Cells were seeded at a density 5000 cells/well (DMEM) in a 96-well half-area white plate (Greiner Bio-One) and left to adhere overnight at 37°C, 5% CO_2. The DMEM was aspirated and **1·TAT** was introduced into each well in quadruplicates. The treated cells were incubated for 4 h at 37°C, 5% CO_2. The HDAC activity assay was conducted using Trichostatin A as inhibitor control, Jurkat Cell Lysate as positive control and referenced against calibration standards according to the *In-Situ* HDAC Activity Fluorometric Assay Kit (Sigma Aldrich). The fluorescence emission was measured using TECAN microplate reader 20 M (λ_{ex} = 368 nm, λ_{em} = 442 nm).

1.15 Annexin V staining assay

The apoptotic nature of cell death was investigated using Annexin V staining after incubation of A549 cells with **1·TAT** at 25 µM. Cells were seeded the day before the assay in an 8-well Ibidi plate at a density of 25,000 cells/well and were incubated overnight at 37 °C to allow them to adhere on the surface. After treatment with **1·TAT** for 4 h, cells were incubated with Annexin-V FITC from the Apoptosis Detection Kit FITC by Invitrogen. After washing the cells once with binding buffer (200 µL), cells in each well were incubated with Annexin V (5 µL) in binding buffer (200 µL) for 15 minutes in the dark. Cells were washed with binding buffer and additionally the cell nuclei were stained using 4',6-diamidino-2-phenylindole (DAPI) in binding buffer, incubating the cells for 5 minutes in the dark. The staining solution was removed and the cells were washed twice with binding buffer. The washing solution was replaced with fresh binding buffer (200 µL) before imaging.

1.16 Cell viability assay

A549, MDA-MB-231, CHO, and HEK cells were both seeded at a density of 2,500 cells/well in a white half area 96-well plate and incubated at 37°C overnight to allow them to adhere. On the next day, different concentrations of the **1·TAT** or cisplatin were prepared in PBS at a 10x concentration of the final concentration, and this solution (20 µL) was added to a fresh medium (180 µL). The old medium of the cells was removed and substituted with a fresh medium (50 µL) containing different

concentrations of sample, prepared as described before. For each condition, triplicates were performed. Cells were incubated with the sample for 4 h or 24 h. After the treatment, CellTiter-Glo® Assay solution (50 µL) was added to each well, and the plate was placed on an orbital shaker for 2 minutes and subsequently incubated 10 minutes at room temperature. Luminescence was measured using a Promega GloMax®-Multi Detection System using manufacturer's protocol.

1.17 Determination of intracellular H_2O_2 concentration

The intracellular H_2O_2 concentration in A549, MDA-MB-231, CHO, and HEK cell lines were determined using the Intracellular Hydrogen Peroxide Assay Kit by Sigma-Aldrich. Cells were seeded in a black cell culture 96 well plate with clear bottom at a density of 10000 cells per well and were left to adhere overnight. Three wells of cells were prepared for each sample (PMA treated and non-treated control cells). To enhance the intracellular hydrogen peroxide concentration cells were treated with 100 nM PMA for 4 h. In order to measure the H_2O_2 concentration the fluorescent peroxide sensor was prepared following the protocol of the vendor and added to the cells after washing them with PBS. After incubation in the dark for 30 min, cells were washed with PBS and fluorescence intensity was recorded using a well plate reader at 525 nm after excitation at 490 nm (bandwidth 10 nm each). To determine the concentration of H_2O_2 inside cells, a standard curve was generated by measuring the fluorescence intensity of a dilution series of hydrogen peroxide at the concentrations of 5, 2.5, 1.25, 0.625, 0.3125, 0.15625 and 0 µM (background blank) after incubation with the fluorescent sensor for 30 min in the dark following the vendor's protocol. All measurements for the standard curve and cells were performed in triplicates, and the background blank was subtracted from all values.

2. Synthesis and characterization details

2.1 Instruments

Nuclear Magnetic Resonance Spectroscopy (NMR). The NMR experiments were measured with a 5 mm QXI $^{1}H/^{13}C/^{15}N/^{19}F$ probe equipped with a z-gradient on the 700.4 MHz and a 5 mm QXI $^{1}H/^{13}C/^{15}N/^{31}P$ probe equipped with a z-gradient on the 850 MHz Bruker AVANCE III system.

For a quantitative ^{1}H NMR (850 MHz) measurements at different temperature 64 transients were used with an 10,8 µs long 90° pulse and a 34000 Hz (40 ppm) spectral width together with a recycling delay of 15 s. The temperatures were calibrated with a standard ^{1}H methanol or ethylene glycol NMR samples using the Topspin 3.2 software (Bruker). The control of the temperature was realized with a VTU (BCU II; variable temperature unit) and an accuracy of +/- 0,1K.

For the proton spectra in the 700 MHz system, 64 transients were used with a 14 µs long 90° pulse and a 12600 Hz (18 ppm) spectral width together with a recycling delay of 8 s, comparable to the 850 MHz system (17000 Hz; 20 ppm, relaxation delay 15 s). The spectroscopic widths of the homonuclear ^{1}H, ^{1}H COSY and ^{1}H, ^{1}H NOESY experiments were typically 10000 Hz (700 MHz; 15 ppm) or 13500 Hz (850 MHz; 16 ppm) in both dimension (f1 and f2) and the relaxation delay is 2 s. The spectroscopic widths of the homo-nuclear ^{1}H, ^{1}H NOESY experiment was typically 13500 Hz (850 MHz; 16 ppm) in both dimension (f1 and f2) and the relaxation delay 2 s. The mixing time used in the ^{1}H, ^{1}H NOESY was kept at 300ms.

Additionally, carbon NMR were measured with a sequence using power gated decoupling (zgpg30) with 12288 number (236 ppm spectral width) of scans and a relaxation delay of 2 s and a 90° pulse of 14 µs on a 700 MHz-magnet system.

For ^{1}H spectra, the chemical shifts are reported in parts per million (ppm) from high to low frequency using the residual solvent peak as the internal reference (D_2O δ = 4.79 ppm and DMSO-d_6 = 2.50 ppm). All ^{1}H resonances are reported to the nearest 0.01 ppm. The multiplicity of ^{1}H signals are indicated as: s = singlet; d = doublet; t = triplet; q = quartet; p = pentet; m = multiplet; br = broad; or combinations of thereof. Coupling constants (*J*) are quoted in Hz and reported to the nearest 0.1 Hz. Where appropriate, averages of the signals from peaks displaying multiplicity were used to calculate the value of the coupling constant. For ^{13}C NMR, the spectra were recorded are reported in parts per million (ppm) from high to low frequency using the residual solvent peak as the internal reference (DMSO-d_6 = 39.5 ppm). All ^{13}C resonances are reported to the nearest 0.1 ppm. Chemical shifts for protons of the compounds were assigned on the basis of COSY and HSQC correlations. The data were processed in MestReNova.

Microwave Peptide Synthesizer. Peptides were synthesized in a Liberty Blue Automated Microwave Peptide Synthesizer by CEM Corporation.

High-Performance Liquid Chromatography (HPLC). The conjugates were purified by preparative HPLC using a setup by Shimadzu. For purification either a ZORBAX Eclipse XDB-C18 HPLC column (9.4 × 250 mm, 5 µm) was used at a flowrate of 4 mL/min or a Phenomenex Gemini 5 µm NX-C18 110 Å 150 × 30 mm was used at a flowrate of 25 mL/min.

High-Performance Liquid Chromatography-Mass Spectrometry (LC-MS). Compounds were analyzed by HPLC-ESI-MS on a LC-MS 2020 by Shimadzu using a Kinetex 2.6 µm EVO C18 100 Å LC 50 × 2.1 mm column. MilliQ water, acidified with 0.1% formic acid and CH_3CN were used as solvents for all measurements. The solvent gradient started with 5% CH_3CN and 95% water. This solvent ratio was kept constant for 2 min, then the CH_3CN content was linearly increased to 95% over 14 min.

Matrix-Assisted Laser Desorption/Ionisation - Time of Flight Mass Spectrometry (MALDI-TOF). All MALDI-TOF spectra were recorded on either a rapifleX MALDI-TOF/TOF from Bruker or MALDI Synapt G2-SI from Waters. Samples were mixed with a saturated solution of the matrix S7 α-cyano-4-hydroxycinnamic acid (CHCA) in H_2O/CH_3CN 1/1 + 0.1% CF_3COOH.

2.2 Reaction scheme for the synthesis of the compounds

a

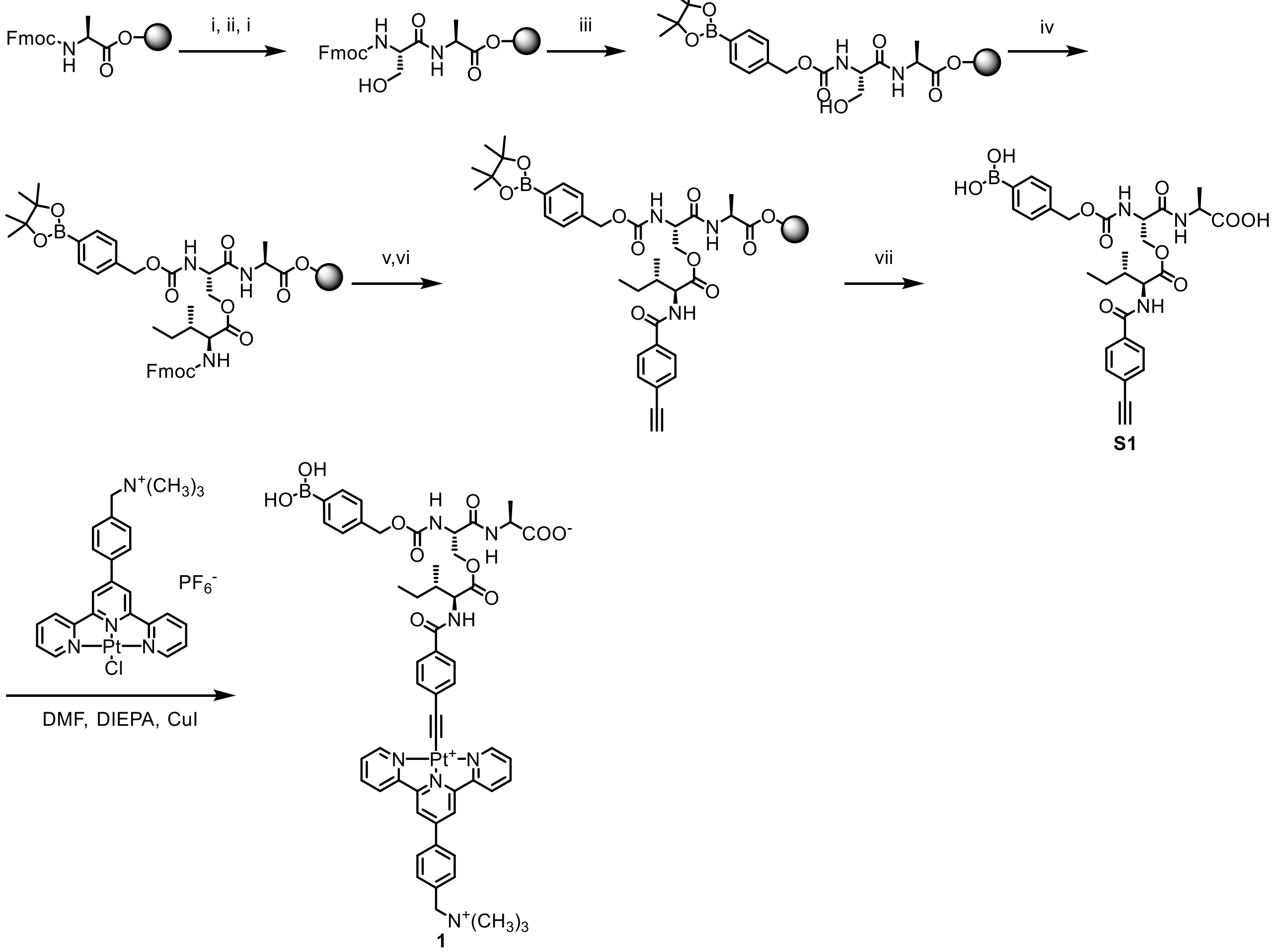

b

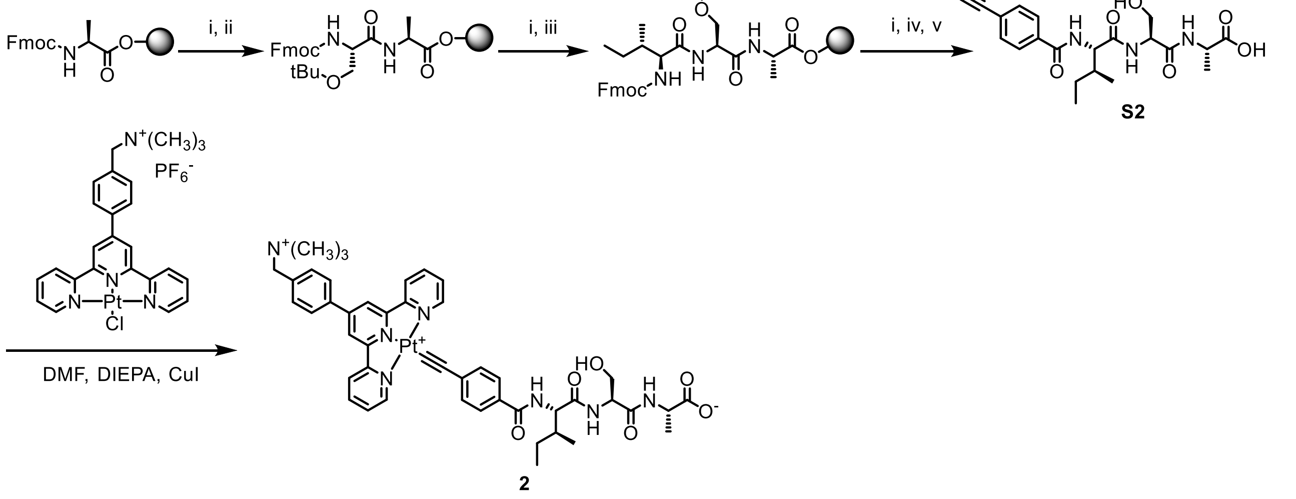

Figure S1. Synthesis of compounds **1** and **2**. **a**, Synthesis of **1**. (i) 20% piperidine in DMF. (ii) Fmoc-serine, PyBOP, DIEPA, DMF, 75 °C, 20 min. (iii) 4-nitrophenyl (4-(4,4,5,5-tetramethyl-1,3,2-dioxaborolan-2- yl)benzyl), DIEPA, DMF, RT. (iv) Fmoc-Ile, DIC, 4-DMAP, RT. (v) 20% piperidine in DMF. (vi) 4-Ethynylbenzoic acid, PyBOP, DIEPA, DMF, 75 °C, 20 min. (vii) 95% TFA, 2.5% TIPS, 2.5% H_2O. **b**, Synthesis of **2**. (i) 20% piperidine in DMF. (ii) Fmoc-Ser(tBu), PyBOP, DIEPA, DMF,

75 °C, 20 min. (iii) Fmoc-Ile, PyBOP, DIEPA, DMF, 75 °C, 20 min. (iv) 4-Ethynylbenzoic acid, PyBOP, DIEPA, DMF, 75 °C, 20 min. (v) 95% TFA, 2.5% TIPS, 2.5% H_2O.

2.3 Synthesis of compound S1

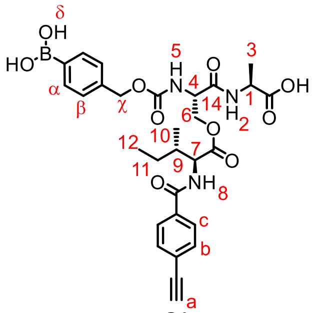

S1

Synthesis: Compound **S1** was synthesized using the Fmoc solid phase peptide synthesis strategy by Merrifield,[2] synthesizing the peptide from *C* to *N*-terminus in a microwave assisted peptide synthesizer. Fmoc-Ala preloaded Wang resin (0.5 mmol) was swollen in DMF for 1 h before use. First, the Fmoc group was removed by two consecutive deprotection steps (2 and 5 min) with 20% piperidine in DMF (10 mL) at 75 °C (i). After deprotection, the resin was washed four times with DMF (7 mL). Fmoc-serine (5 equiv in 10 mL DMF) was coupled to the *N*-terminus by using the activator PyBOP (5 equiv in 4 mL DMF) and activator base DIPEA (10 equiv in 2 mL DMF) at 75 °C for 20 min (ii). After deprotection of the Fmoc protecting group (i), the *N* terminus was modified using 4-nitrophenyl (4-(4,4,5,5-tetramethyl-1,3,2-dioxaborolan-2- yl)benzyl) carbonate (1.25 equiv) in 10 mL DMF and DIPEA (5 equiv) overnight at room temperature (iii). After the resin was washed with DMF and DCM, Fmoc-Ile (10 equiv in 5 mL DMF), DIC (10 equiv) and 4-DMAP (1 equiv) were added to form the ester bond in the serine side chain. After the reaction mixture was stirred for 12 h at room temperature, the resin was washed with DMF and DCM, dried (iv). The Fmoc group was then removed in two deprotection steps using 20% piperidine in DMF (10 min each, 5 mL) at room temperature (v). 4-Ethynylbenzoic acid (1.2 equiv) was coupled onto the *N*-terminus overnight at room temperature, using PyBOP (2 equiv) and DIPEA (4 equiv) as the coupling reagents. The resin was washed with DMF and DCM, dried (vi). The product was cleaved from the resin by using 2.5 mL of a cleavage cocktail (95% TFA, 2.5% TIPS, 2.5% H_2O). This step also removed the pinacol protecting group of the PBA. After 2 hours, the cleavage cocktail was removed in vacuo (vii). The product was purified by HPLC using the Zorbax Eclipse column at a flowrate of 4 mL/min. The gradient started with 20% CH_3CN in H_2O (+0.1% CF_3COOH) and this solvent ratio was kept for 1 min, after which the CH_3CN content was increased to 100% over 15 min. The product **S1** eluted from the column after 11.8 min and was received as a white powder after lyophilization (18.2 mg, 6.1% overall yield).

^{1}H NMR (700 MHz, DMSO-d_6, 298 K): δ 8.63 (H^{8}, d, J = 7.9 Hz, 1H), 8.37 (H^{5}, d, J = 7.3 Hz, 1H), 7.87 (H^{c}, d, J = 8.1 Hz, 2H), 7.78 (H^{α}, d, J = 7.6 Hz, 2H), 7.61 (H^{5}, d, J = 8.6 Hz, 1H), 7.56 (H^{b}, d, J = 8.0 Hz, 2H), 7.31 (H^{β}, d, J = 7.6 Hz, 2H), 5.04 (H^{χ}, s, 2H), 4.32 – 4.35 (H^{4} and H^{7}, m, 2H), 4.37 (H^{a}, s, 1H), 4.32 (H^{6}, dd, J = 11.2, 4.4 Hz, 1H), 4.24 – 4.17 (H^{1}, m, 1H), 4.11 ($H^{6'}$, dd, J = 11.0, 8.0 Hz, 1H), 1.94 (H^{9}, dq, J = 11.5, 6.9 Hz, 1H), 1.48 (H^{11}, dtd, J = 13.7, 7.4, 4.4 Hz, 1H), 1.30 – 1.20 (H^{3} and H^{11}, m, 4H), 0.92 – 0.86 (H^{10}, m, 3H), 0.84 (H^{12}, t, J = 7.5 Hz, 3H).

^{13}C NMR (176 MHz, DMSO-d_6, 298 K): δ 173.7, 171.1, 168.4, 166.2, 155.9, 138.6, 134.2, 134.0, 131.5, 128.0, 128.0, 126.5, 124.6, 82.9, 65.6, 63.9, 57.4, 53.6, 47.7, 40.0, 35.8, 25.1, 17.1, 15.4, 10.9.

LCMS: LC retention time (min): 8.14. MS (ESI$^+$, *m*/*z*): 596.3 [M + H]$^+$, 618.3 [M + Na]$^+$.

HRMS (ESI$^+$, *m*/*z*): Calculated for [M + H]$^+$ ($C_{29}H_{35}BN_3O_{10}^+$): 596.2421, found 596.2460. Calculated for [M + Na]$^+$ ($C_{29}H_{34}BN_3O_{10}Na^+$): 618.2240, found 618.2284.

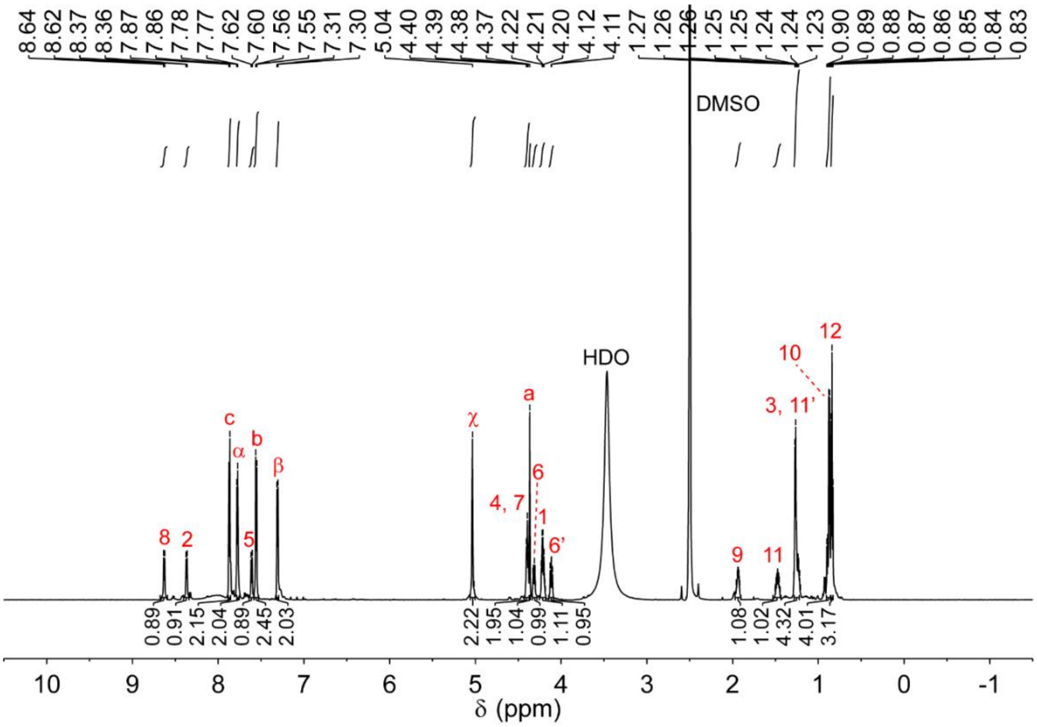

Figure S2. ^{1}H NMR spectrum (700 MHz, DMSO-d_6, 298 K) of compound **S1**.

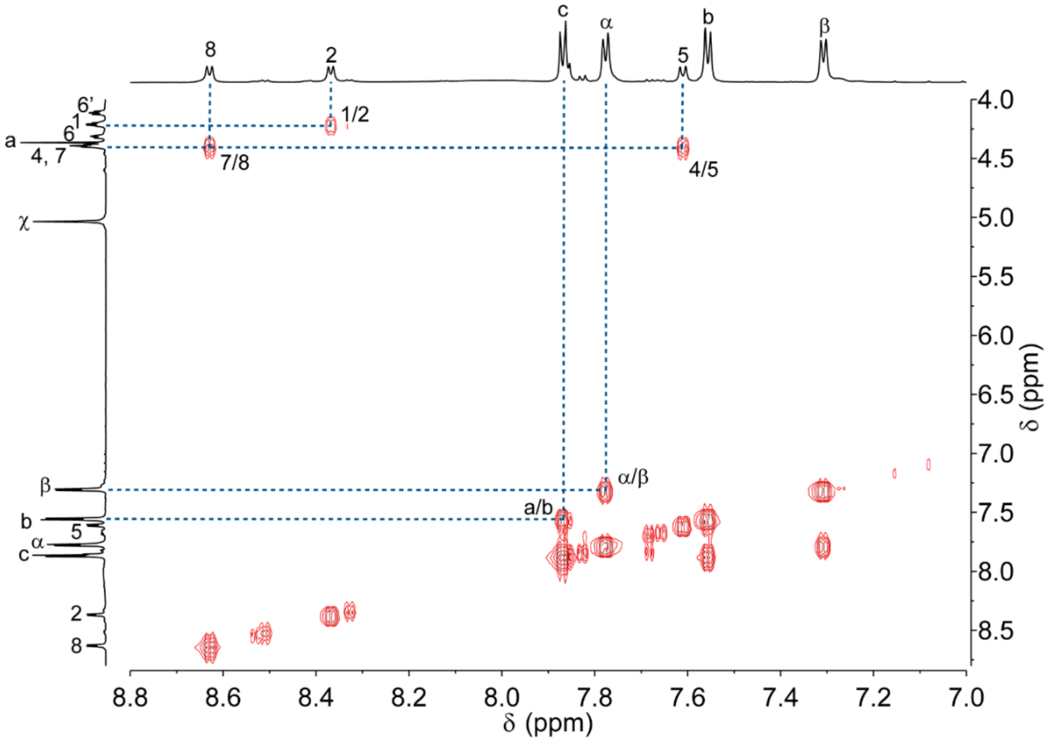

Figure S3. Partial ^{1}H NMR and $^{1}H,^{1}H$ COSY NMR spectra (700 MHz, DMSO-d_6, 298 K) of compound **S1** (aromatic region). The correlations between ^{1}H signals are highlighted with dashed lines in the COSY spectrum.

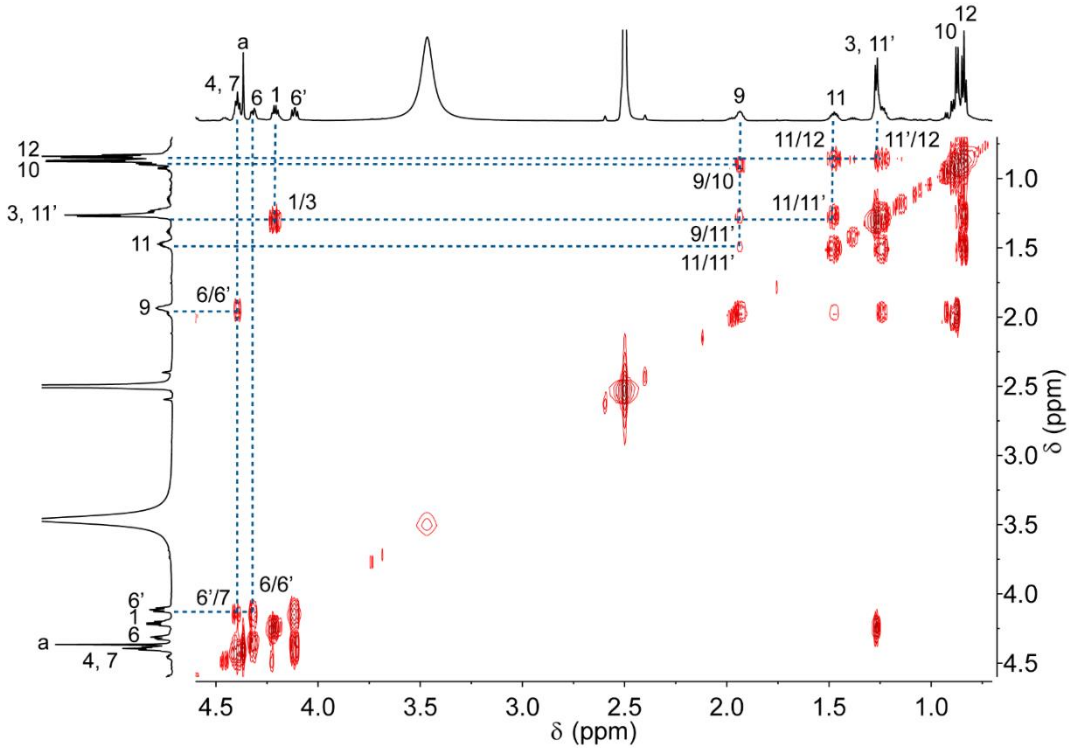

Figure S4. Partial ^{1}H NMR and $^{1}H,^{1}H$ COSY NMR spectra (700 MHz, DMSO-d_6, 298 K) of compound **S1** (aliphatic region). The correlations between ^{1}H signals are highlighted with dashed lines in the COSY spectra.

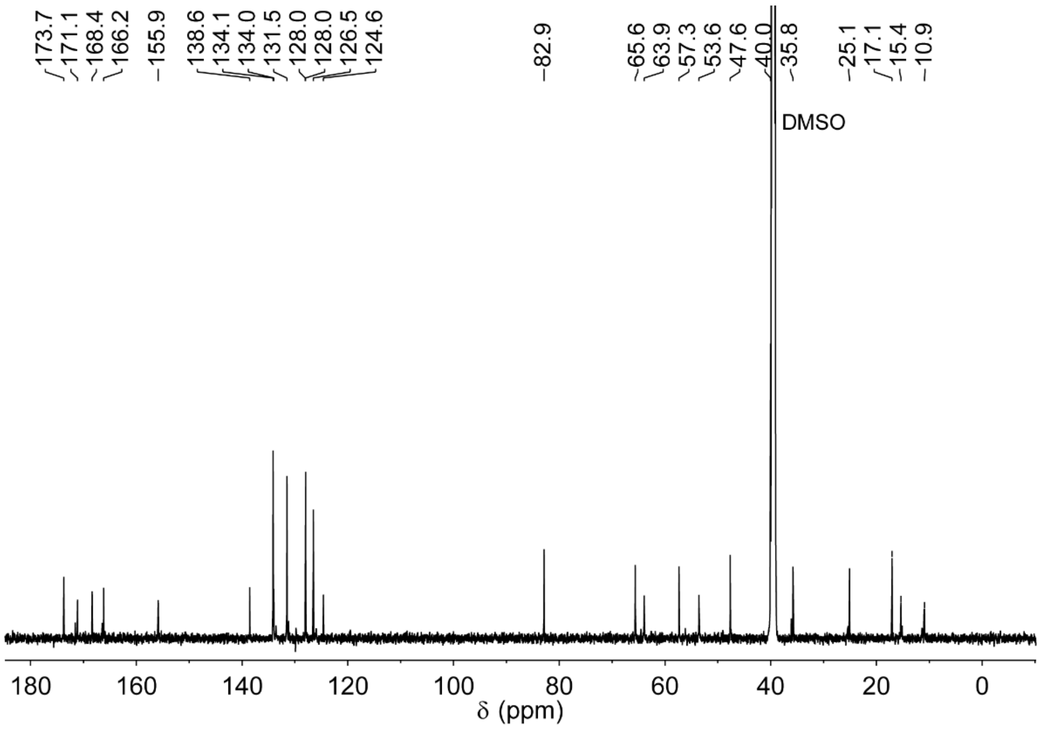

Figure S5. ^{13}C NMR spectrum (175 MHz, DMSO-d_6, 298 K) of compound **S1**.

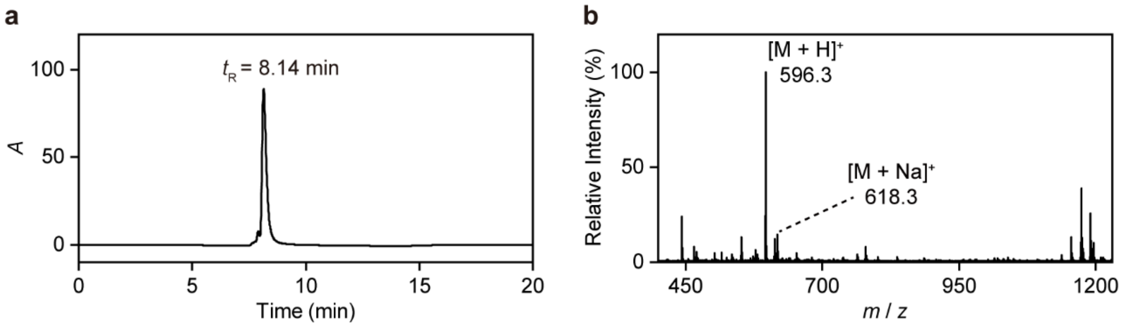

Figure S6. LC-MS study of compound **S1**. **a**, HPLC trace of **S1.** Retention time (t_R) = 8.14 min. **b**, Convoluted MS spectrum for t_R = 8.10-8.30 min, showing peaks of [M + H]$^+$ and [M + Na]$^+$.

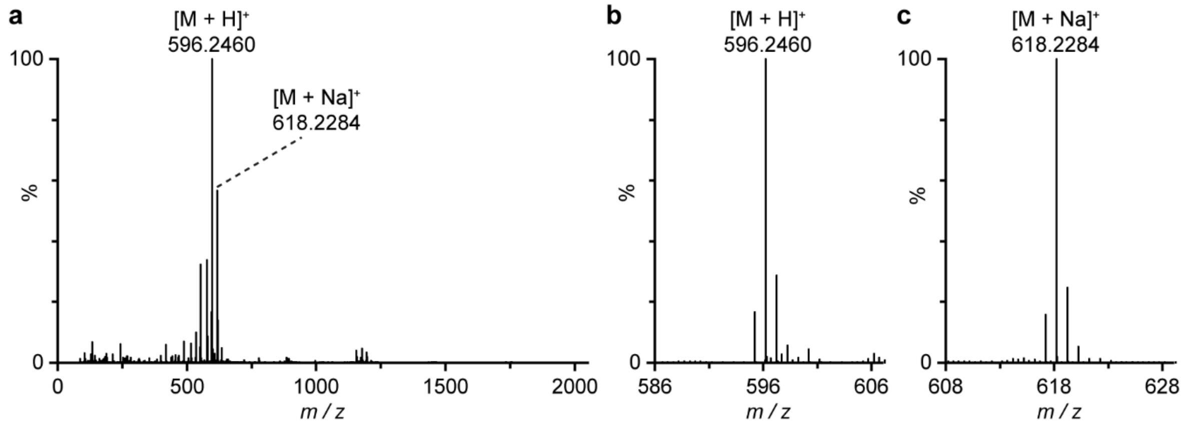

Figure S7. High-resolution MS spectrum of **S1**. **a**, Full MS spectrum. **b**, partial MS spectrum showing [M + H]$^+$ and [M + Na]$^+$.

2.4 Synthesis of compound 1

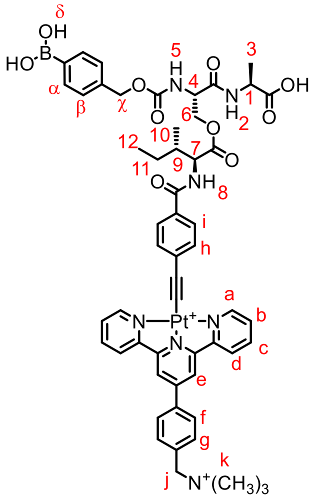

1

Synthesis: Compound **S1** (11.9 mg, 0.02 mmol), the Pt(tpy)Cl complex (15.1 mg, 0.02 mmol), DIEPA (12.9 mg, 0.10 mmol), DMF (5 mL), and a catalytic amount of CuI were placed in a Schlenk flask. After filling the flask with Ar gas using the freeze-pump-thaw method, the mixture was heated to 50 °C for 24 h. The mixture was cooled and Et_2O was added to the mixture. The precipitate was collected, and the product was purified by HPLC using the Zorbax Eclipse column at a flowrate of 4 mL/min. The gradient started with 20% CH_3CN in H_2O (+0.1% CF_3COOH) and this solvent ratio was kept for 1 min, after which the CH_3CN content was increased to 100% over 15 min. The compound **1** (with CF_3COO^- counterion) eluted from the column after 10.2 min and was received as a dark brown powder after lyophilization (8.1 mg, 29.0% yield).

^{1}H NMR δ (700 MHz, DMSO-d_6, 298 K): 9.08 (H^a and H^e, s, 4H), 8.86 (H^d, d, J = 7.5 Hz, 2H), 8.59 – 8.51 (H^c and H^8,m, 3H), 8.39 (H^2, d, J = 6.2 Hz, 1H), 8.29 (H^f, d, J = 7.5 Hz, 2H), 7.95 – 7.89 (H^b, m, 2H), 7.85 (H^i, d, J = 7.8 Hz, 2H), 7.82 (H^g, d, J = 7.6 Hz, 2H), 7.79 (H^α, d, J = 7.2 Hz, 2H), 7.66 (H^5, d, J = 8.4 Hz, 1H), 7.53 (H^h, d, J = 7.7 Hz, 2H), 7.33 (H^β, d, J = 7.0 Hz, 2H), 5.06 (H^χ, s, 2H), 4.66 (H^j, s, 2H), 4.48 – 4.40 (H^4 and H^7, m, 3H), 4.39 – 4.33 (H^6, m, 1H), 4.25 – 4.20 (H^1, m, 1H), 4.17 – 4.11 ($H^{6'}$, m, 1H), 3.12 (H^k, s, 9H), 2.06 – 1.91 (H^9, m, 1H), 1.73 (H^{11}, s, 1H), 1.57 – 1.46 (H^3 and $H^{11'}$, m, 4H), 0.96 – 0.90 (H^{10}, m, 3H), 0.88 (H^{12}, t, J = 9.6 Hz, 3H).

^{13}C NMR (176 MHz, DMSO-d_6, 298 K): δ 173.8, 171.4, 168.4, 166.4, 158.6, 157.8, 157.6, 155.9, 154.3, 154.2, 151.8, 147.6, 142.1, 138.6, 136.9, 134.2, 133.7, 131.4, 131.3, 129.9, 128.3, 127.7, 126.5, 126.1, 121.6, 67.2, 65.7, 57.3, 53.6, 52.1, 47.7, 45.9, 40.0, 35.8, 25.9, 25.2, 17.1, 15.5, 10.9.

LCMS: LC retention time (min): 6.54 MS (ESI$^+$, m/z): 585.3 $[M + H]^{2+}$, 1169.6 $[M]^+$.

HRMS (ESI$^+$, m/z): Calculated for $[M + H]^{2+}$ ($C_{54}H_{58}BN_7O_{10}Pt^+$): 585.7039, found 585.7054. Calculated for $[M]^+$: ($C_{54}H_{57}BN_7O_{10}Pt^+$): 1169.3921, found 1169.3925.

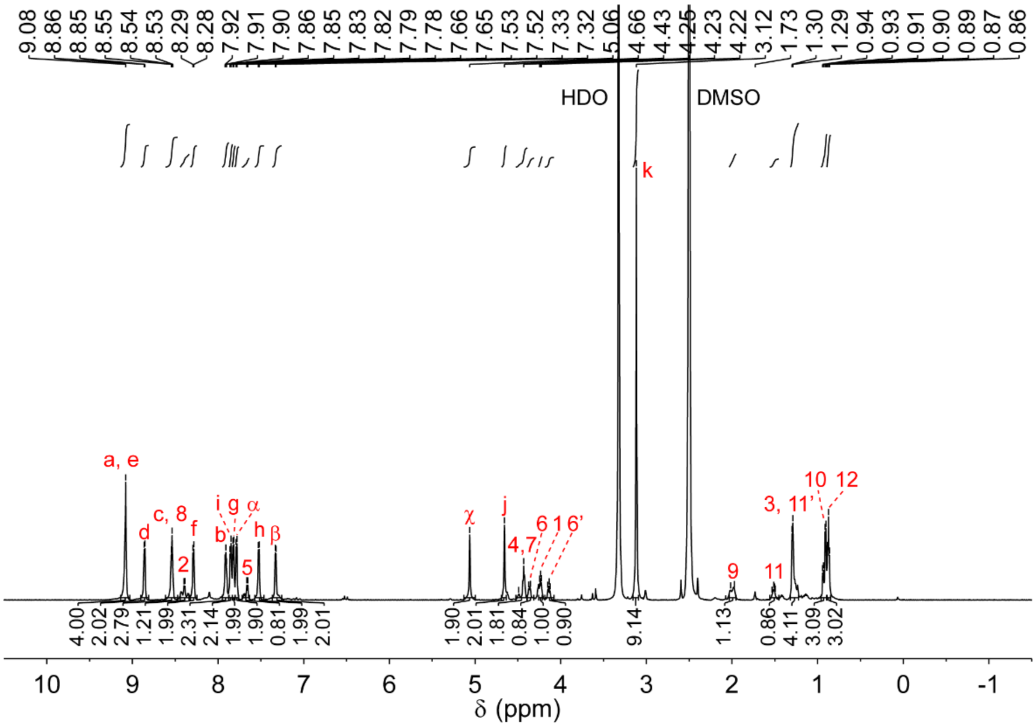

Figure S8. ^{1}H NMR spectrum (700 MHz, DMSO-d_6, 298 K) of compound **1**.

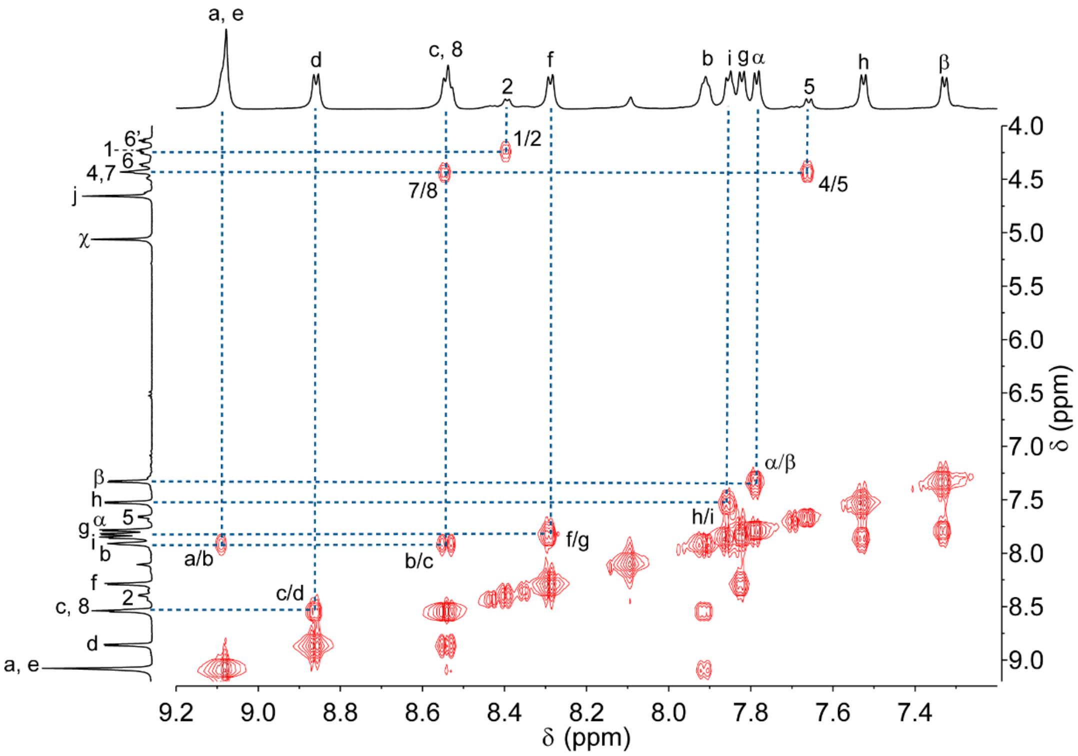

Figure S9. Partial ^{1}H NMR and ^{1}H,^{1}H COSY NMR spectra (700 MHz, DMSO-d_6, 298 K) of compound **1** (aromatic region). The correlations between ^{1}H signals are highlighted with dashed lines in the COSY spectra.

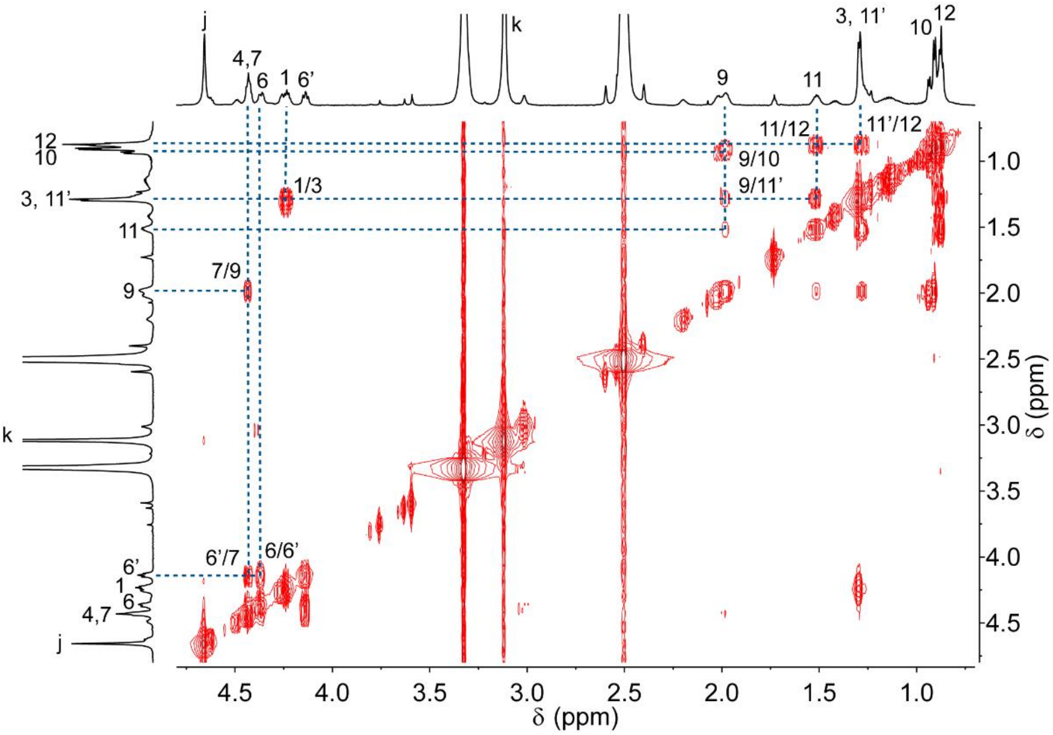

Figure S10. Partial ^{1}H NMR and $^{1}H,^{1}H$ COSY NMR spectra (700 MHz, DMSO-d_6, 298 K) of compound **1** (aliphatic region). The correlations between ^{1}H signals are highlighted with dashed lines in the COSY spectra.

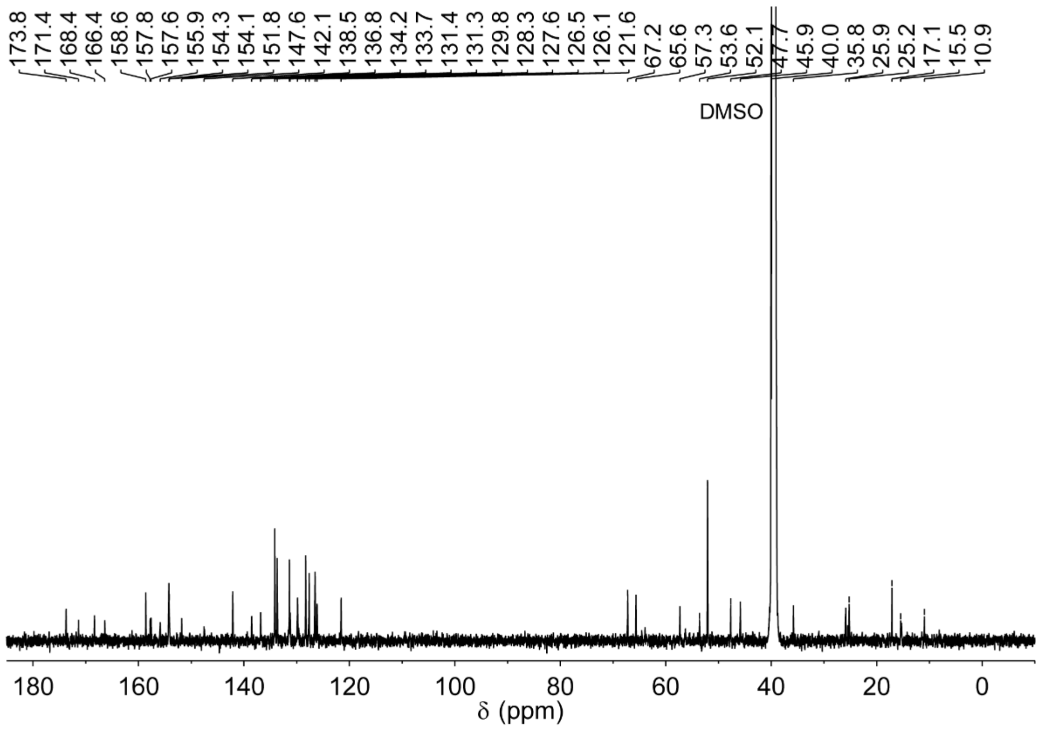

Figure S11. ^{13}C NMR spectrum (175 MHz, DMSO-d_6, 298 K) of compound **1**.

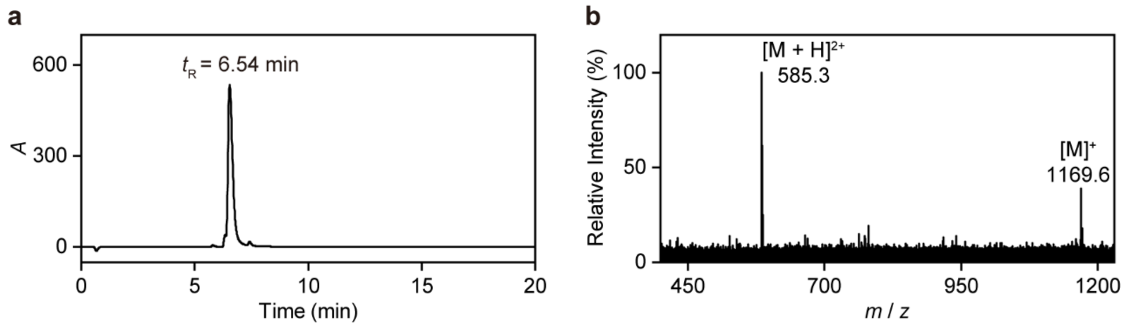

Figure S12. LC-MS study of compound **1**. **a**, HPLC trace of **1.** Retention time (t_R) = 6.54 min. **b**, Convoluted MS spectrum for t_R = 6.52-6.60 min, showing peaks of $[M + H]^{2+}$ and $[M]^{+}$.

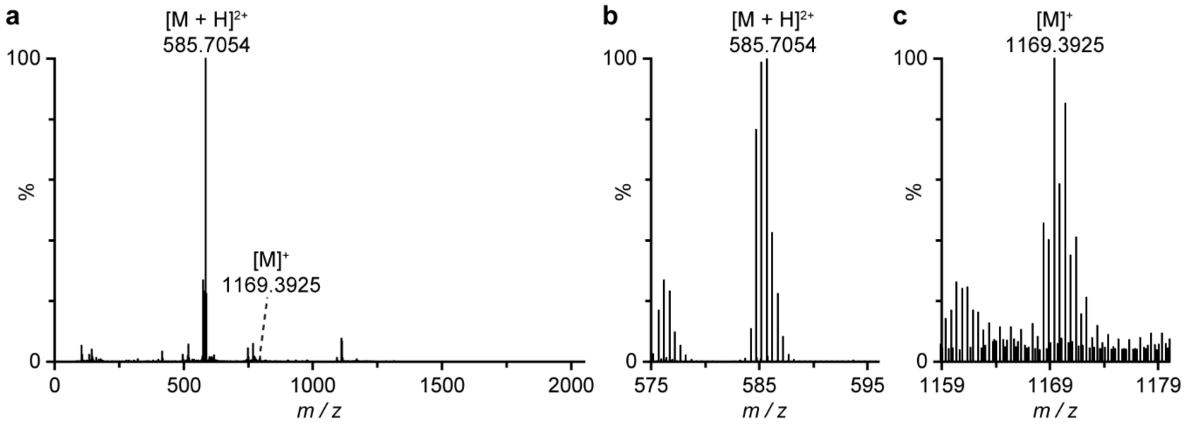

Figure S13. High-resolution MS spectrum of **1**. **a**, Full MS spectrum. **b**, partial MS spectrum showing $[M + H]^{2+}$ and $[M]^{+}$.

2.5 Synthesis of compound S2

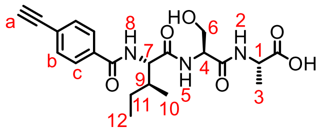

S2

Synthesis: Compound **S2** was synthesized using the Fmoc solid phase peptide synthesis strategy by Merrifield,[2] synthesizing the peptide from *C* to *N*-terminus in a microwave assisted peptide synthesizer at a 0.25 mmol scale. Fmoc-Ala preloaded Wang resin (0.5 mmol) was swollen in DMF for 1 h before use. After deprotection of the Fmoc group (3 mL of 20% piperidine in DMF, 2 and 5 min at 75 °C) (i), Fmoc-Ser(tBu) (ii), Fmoc-Ile (iii), or 4-ethynylbenzoic acid (iv) were double coupled onto the peptide at 75 °C for 20 min. 5 equiv of the compounds were used in 2.5 mL DMF. PyBOP (5 equiv in 1 mL DMF) and DIPEA (10 equiv in 0.5 mL) were used for the coupling reaction. The product was cleaved from the resin by using 2.5 mL of a cleavage cocktail (95% TFA, 2.5% TIPS, 2.5% H_2O). This step also removed the pinacol protecting group of the PBA. After 2 h, the cleavage cocktail was removed in vacuo (v). The product was purified by HPLC using the Zorbax Eclipse column at a flowrate of 4 mL/min. The gradient started with 20% CH_3CN in H_2O (+0.1% CF_3COOH) and this solvent ratio was kept for 1 min, after which the CH_3CN content was increased

to 100% over 15 min. The compound **S2** eluted from the column after 9.1 min and was received as a white powder after lyophilization (68.0 mg, 65.2% overall yield).

^{1}H NMR (700 MHz, DMSO-*d*$_6$, 298 K): δ 8.43 (H^8, d, *J* = 8.5 Hz, 1H), 8.06 (H^5,d, *J* = 7.8 Hz, 1H), 7.99 (H^2,d, *J* = 7.2 Hz, 1H), 7.88 (H^c, d, *J* = 8.0 Hz, 2H), 7.57 (H^b, d, *J* = 8.0 Hz, 2H), 4.41 – 4.36 (H^a and H^7, m, 2H), 4.33 (H^4, q, *J* = 6.2 Hz, 1H), 4.20 (H^1, p, *J* = 7.2 Hz, 1H), 3.64 – 3.53 (H^6, m, 2H), 1.96 – 1.87 (H^9, m, 1H), 1.54 – 1.46 (H^{11}, m, 1H), 1.25 (H^3, d, *J* = 7.2 Hz, 3H), 1.17 ($H^{11'}$, dp, *J* = 14.9, 7.3 Hz, 1H), 0.91 (H^{10}, d, *J* = 6.7 Hz, 3H), 0.83 (H^{12}, t, *J* = 7.4 Hz, 3H).

^{13}C NMR (176 MHz, DMSO-*d*$_6$, 298 K): δ 173.9, 171.0, 169.5, 165.7, 134.3, 131.5, 127.8, 124.5, 82.8, 61.6, 58.0, 54.9, 47.5, 40.0, 36.0, 24.8, 17.3, 15.4, 10.8.

LCMS: LC retention time (min): 6.97. MS (ESI$^+$, *m*/*z*): 418.2 [M + H]$^+$, 440.2 [M + Na]$^+$.

HRMS (ESI$^+$, *m*/*z*): Calculated for [M + H]$^+$ ($C_{21}H_{28}N_3O_6^+$): 418.1978, found 418.2006. Calculated for [M + Na]$^+$ ($C_{21}H_{27}N_3O_6Na^+$): 440.1797, found 440.1817.

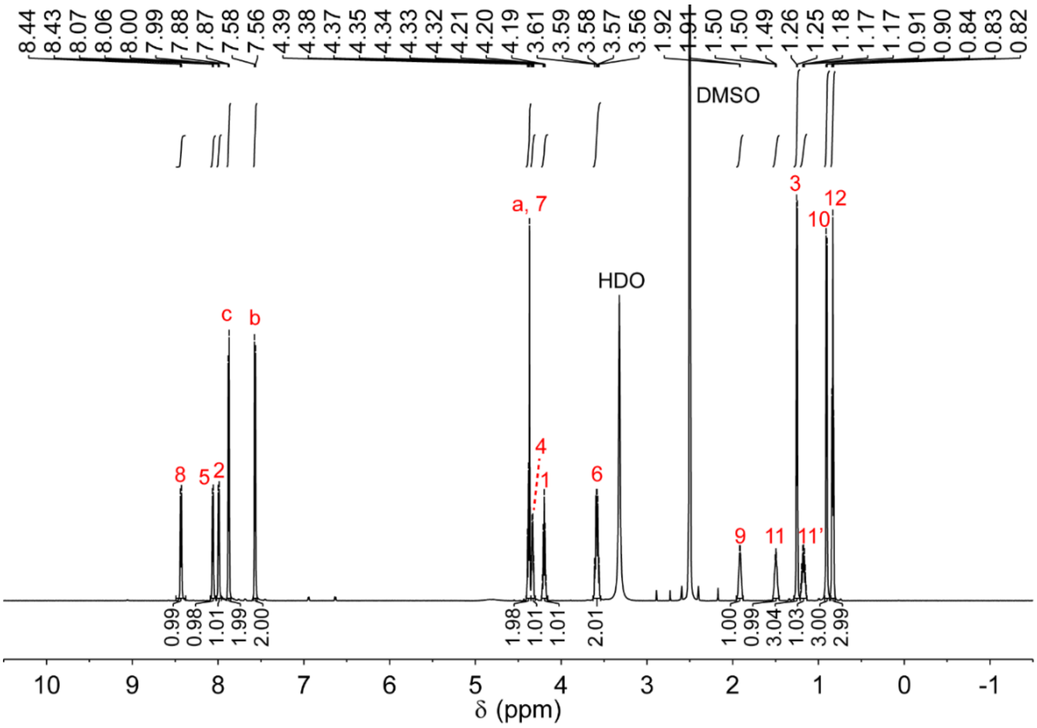

Figure S14. ^{1}H NMR spectrum (700 MHz, DMSO-*d*$_6$, 298 K) of compound **S2**.

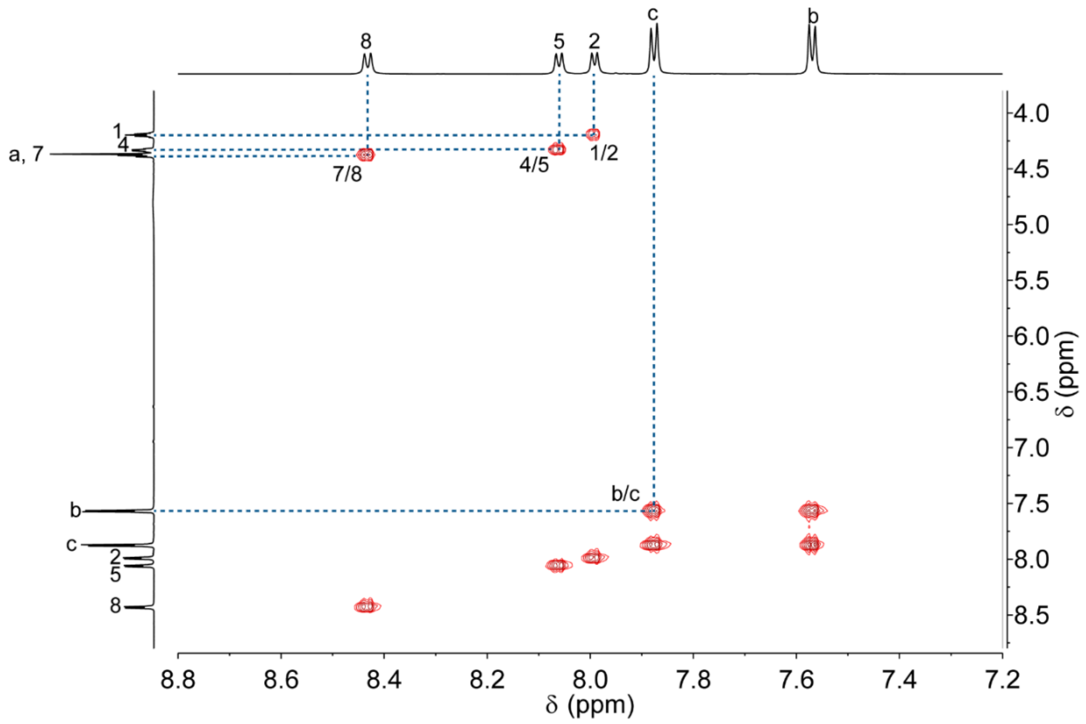

Figure S15. Partial ^{1}H NMR and $^{1}H,^{1}H$ COSY NMR spectra (700 MHz, DMSO-d_6, 298 K) of compound **S2** (aromatic region). The correlations between ^{1}H signals are highlighted with dashed lines in the COSY spectra.

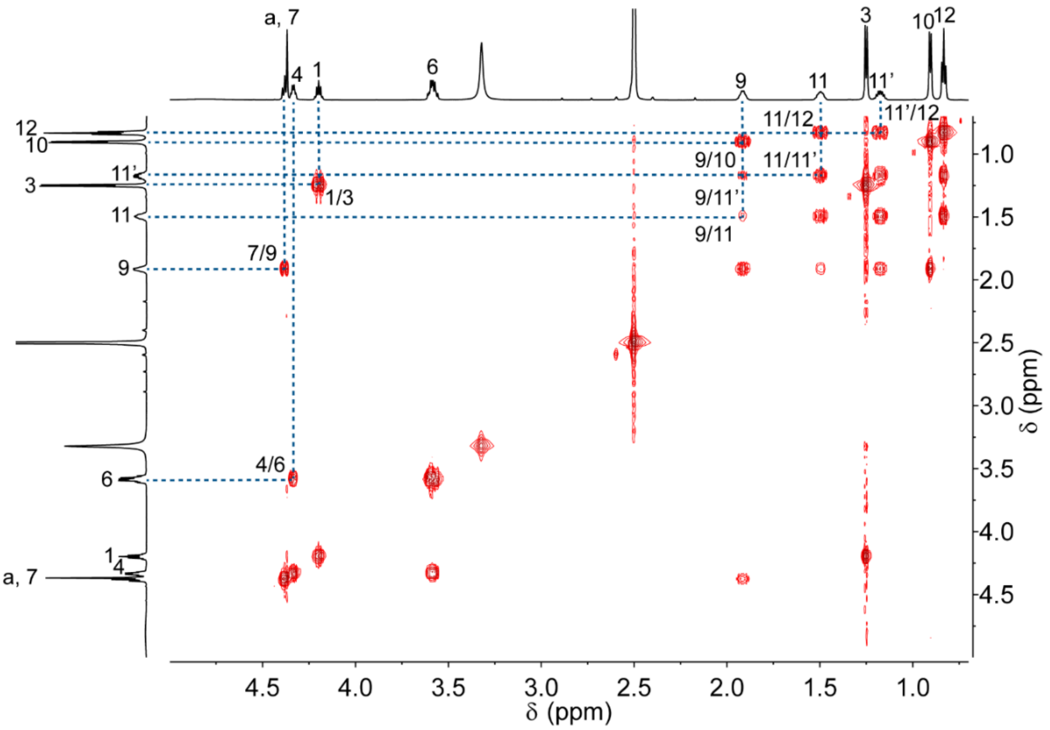

Figure S16. Partial ^{1}H NMR and $^{1}H,^{1}H$ COSY NMR spectra (700 MHz, DMSO-d_6, 298 K) of compound **S2** (aliphatic region). The correlations between ^{1}H signals are highlighted with dashed lines in the COSY spectra.

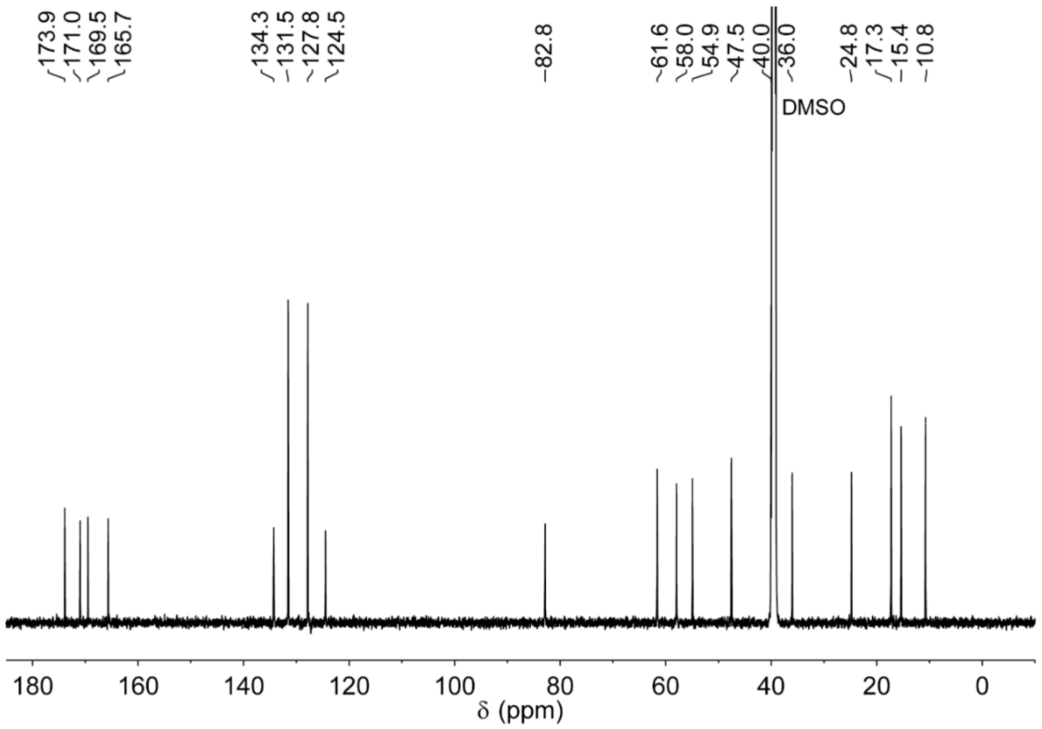

Figure S17. ^{13}C NMR spectrum (175 MHz, DMSO-d_6, 298 K) of compound **S2**.

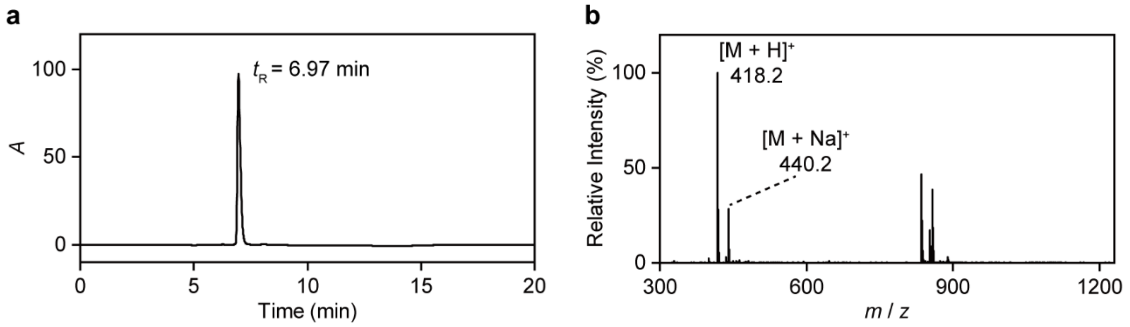

Figure S18. LC-MS study of compound **S2**. **a**, HPLC trace of **S2.** Retention time (t_R) = 6.97 min. **b**, Convoluted MS spectrum for t_R = 6.95-7.10 min, showing peaks of $[M + H]^+$ and $[M + Na]^+$.

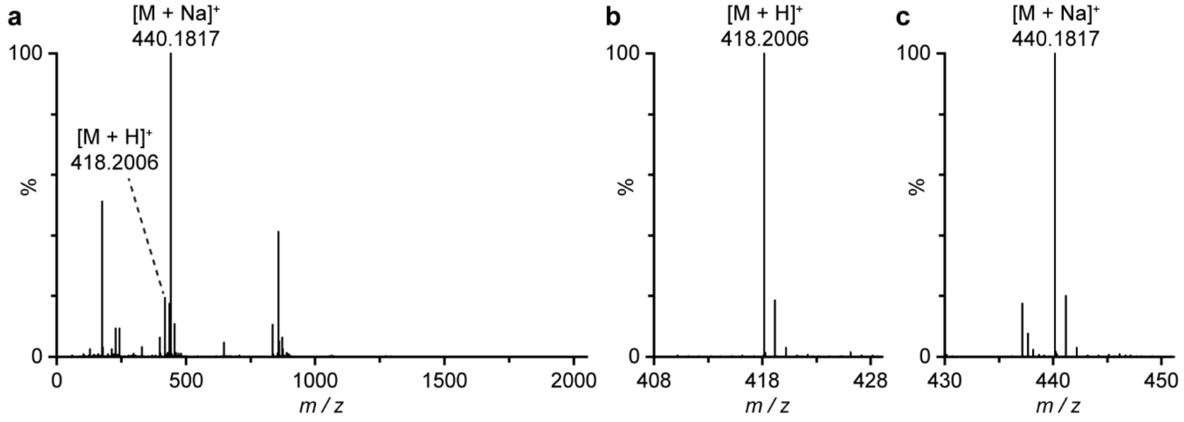

Figure S19. High-resolution MS spectrum of **S2**. **a**, Full MS spectrum. **b**, partial MS spectrum showing $[M + H]^+$ and $[M + Na]^+$.

2.6 Synthesis of compound 2

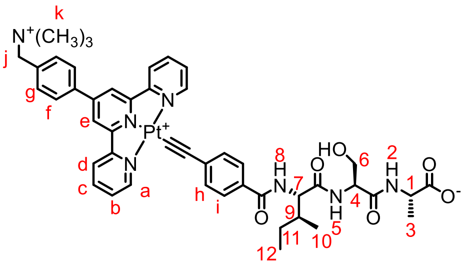

2

Synthesis: Compound **S2** (20.9 mg, 0.05 mmol), the Pt(tpy)Cl complex (37.8 mg, 0.05 mmol), DIEPA (32.3 mg, 0.25 mmol), DMF (10 mL), and a catalytic amount of CuI were placed in a Schlenk flask. After filling the flask with Ar gas using the freeze-pump-thaw method, the mixture was heated to 50 °C for 24 h. The mixture was cooled and Et_2O was added to the mixture. The precipitate was collected, and the product was purified by HPLC using the Zorbax Eclipse column at a flowrate of 4 mL/min. The gradient started with 10% CH_3CN in H_2O (+0.1% TFA) and this solvent ratio was kept for 1 min, after which the CH_3CN content was increased to 100% over 15 min. The compound **2** eluted from the column after 8.5 min and was received as a dark brown powder (27.1 mg, 51.6% yield) after lyophilization in a NH_4HCO_3 buffer solution.

^{1}H NMR (850 MHz, DMSO-d_6, 323 K): δ 9.26 (H^a, d, J = 5.6 Hz, 2H), 9.10 (H^e, s, 2H), 8.89 (H^d, d, J = 7.9 Hz, 2H), 8.59 (H^c, t, J = 8.5 Hz, 2H), 8.34 (H^f, d, J = 8.3 Hz, 2H), 8.20 (H^8, d, J = 8.4 Hz, 1H), 8.02 – 7.98 (H^b, m, 2H), 7.96 (H^5, d, J = 7.9 Hz, 1H), 7.90 – 7.88 (H^i and H^2, m, 3H), 7.86 (H^g, d, J = 8.4 Hz, 2H), 7.60 (H^h, d, J = 8.2 Hz, 2H), 4.67 (H^j, s, 2H), 4.43 (H^7, t, J = 8.4 Hz, 1H), 4.40 – 4.35 (H^4, m, 1H), 4.25 (H^1, p, J = 7.3 Hz, 1H), 3.63 (H^6, d, J = 6.1 Hz, 2H), 3.13 (H^k, s, 9H), 2.03 – 1.93 (H^9, m, 1H), 1.61 – 1.51 (H^{11}, m, 1H), 1.28 (H^3, d, J = 7.1 Hz, 3H), 1.27 – 1.15 ($H^{11'}$, m, 1H), 0.95 (H^{10}, d, J = 6.7 Hz, 3H), 0.88 (H^{12}, t, J = 7.5 Hz, 3H).

^{13}C NMR (175 MHz, DMSO-d_6, 323 K): δ 170.9, 169.2, 165.7, 158.3, 157.3, 153.9, 153.6, 151.6, 141.7, 136.5, 133.3, 131.6, 131.1, 130.9, 129.4, 129.2, 128.0, 127.1, 125.9, 121.4, 67.3, 61.7, 58.1, 54.9, 52.1, 48.5, 45.9, 39.8, 36.0, 24.6, 17.6, 15.3, 10.5.

LCMS: LC retention time (min): 6.06. MS (ESI$^+$, m/z): 496.4 $[M + H]^{2+}$, 991.4 $[M]^+$.

HRMS (ESI$^+$, m/z): Calculated for $[M + H]^{2+}$ ($C_{46}H_{51}N_7O_6Pt^{2+}$): 496.1776, found 496.1817. Calculated for $[M]^+$ ($C_{46}H_{50}N_7O_6Pt^+$): 991.3474, found 991.3532.

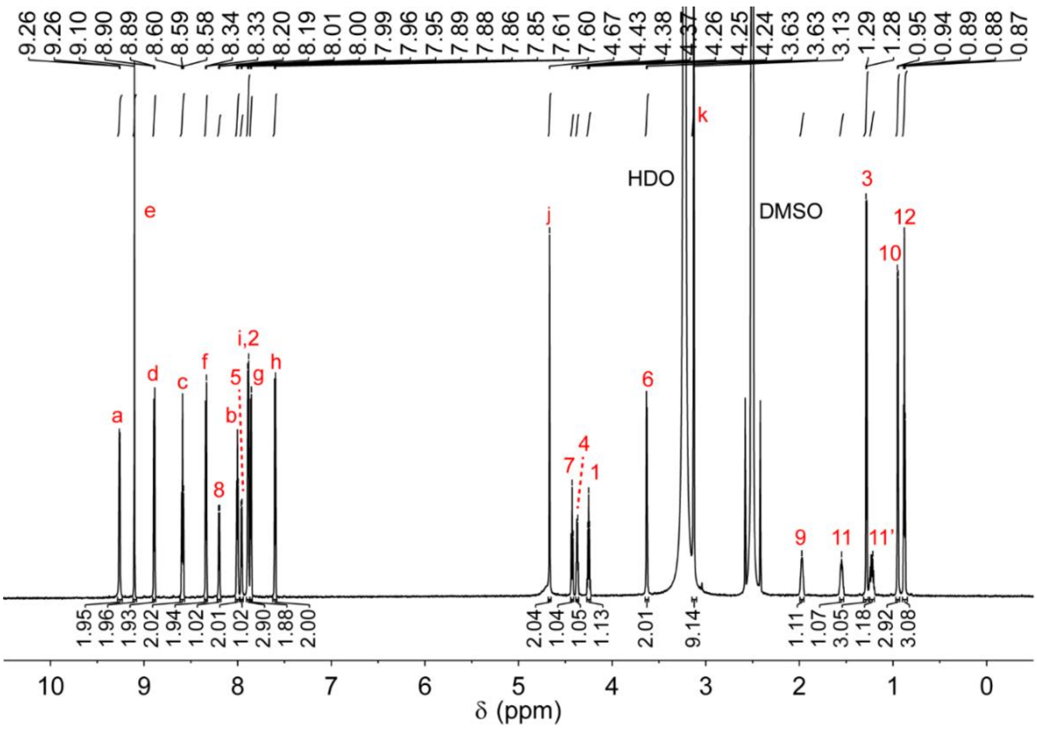

Figure S20. ^{1}H NMR spectrum (850 MHz, DMSO-d_6, 298 K) of compound **2**.

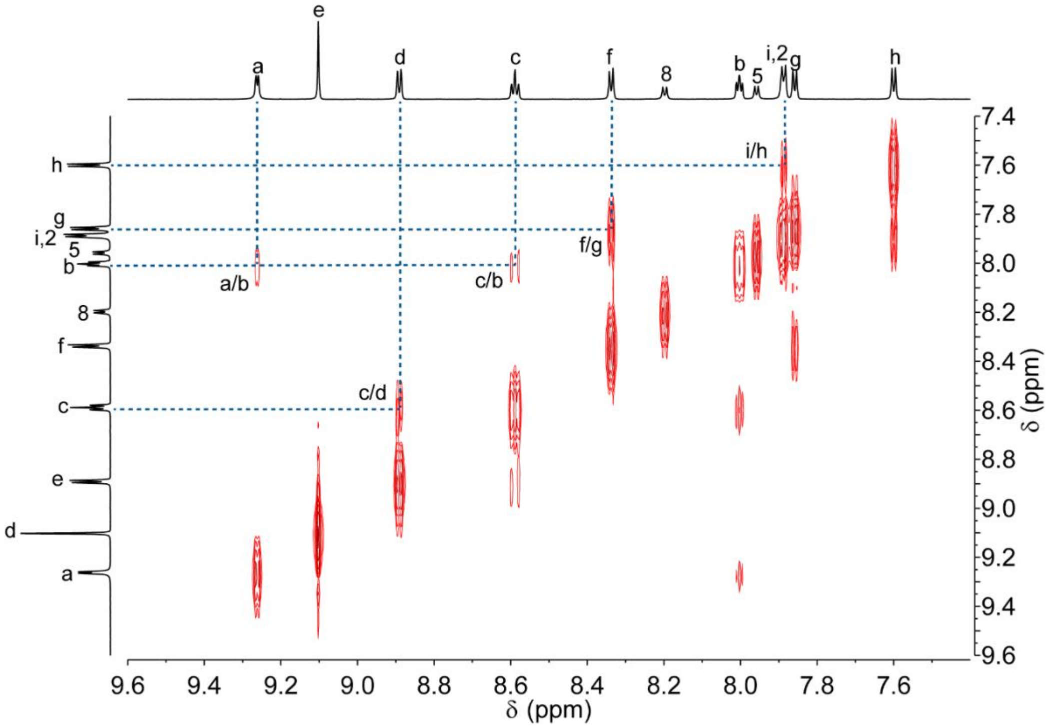

Figure S21. Partial ^{1}H NMR and ^{1}H,^{1}H COSY NMR spectra (850 MHz, DMSO-d_6, 298 K) of compound **2** (aromatic region). The correlations between ^{1}H signals are highlighted with dashed lines in the COSY spectra.

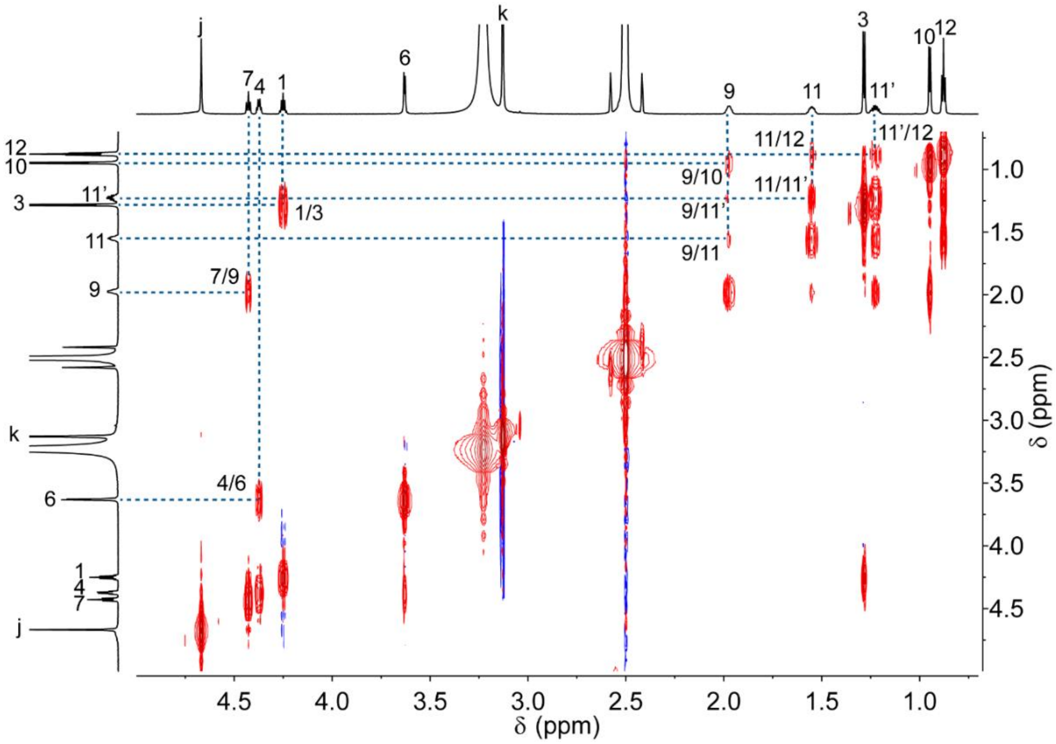

Figure S22. Partial ^{1}H NMR and $^{1}H,^{1}H$ COSY NMR spectra (850 MHz, DMSO-d_6, 298 K) of compound **2** (aliphatic region). The correlations between ^{1}H signals are highlighted with dashed lines in the COSY spectra.

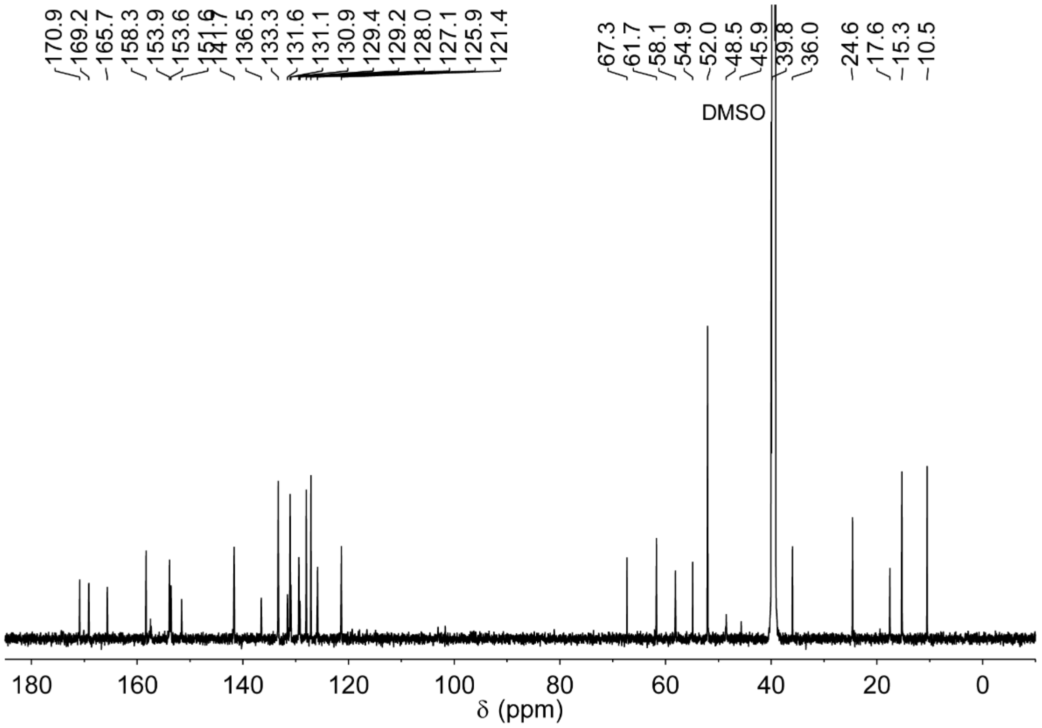

Figure S23. ^{13}C NMR spectrum (213 MHz, DMSO-d_6, 298 K) of compound **2**.

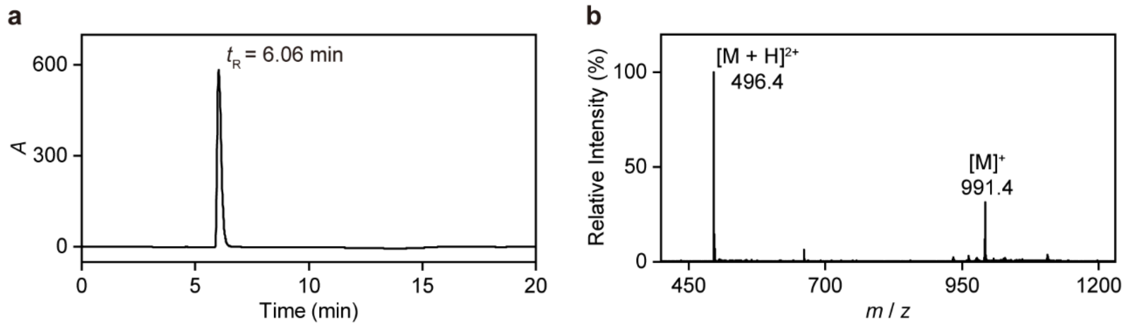

Figure S24. LC-MS study of compound **2**. **a**, HPLC trace of **2.** Retention time (t_R) = 6.97 min. **b**, Convoluted MS spectrum for t_R = 6.95-7.10 min, showing peaks of [M + H]$^{2+}$ and [M]$^+$.

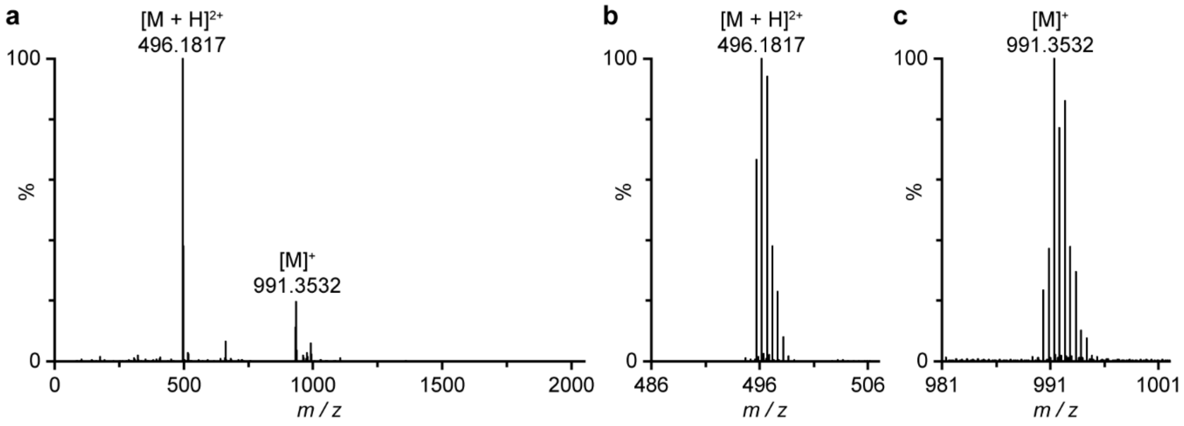

Figure S25. High-resolution MS spectrum of **2**. **a**, Full MS spectrum. **b**, partial MS spectrum showing [M + H]$^{2+}$ and [M]$^+$.

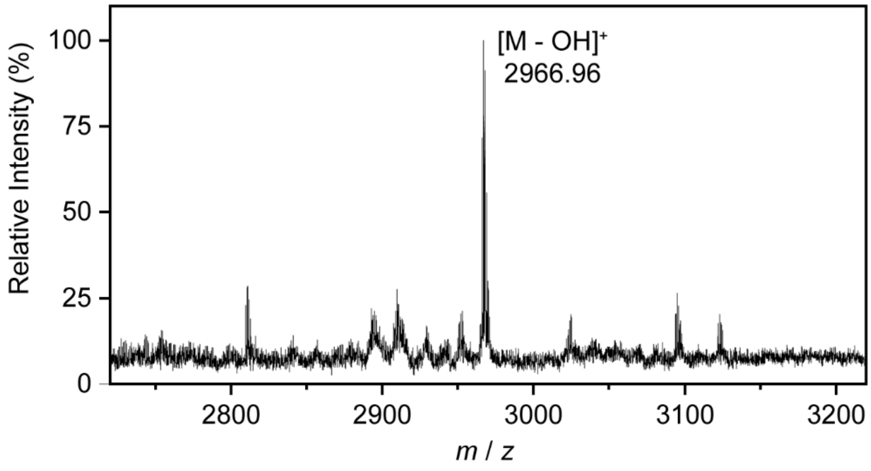

Figure S26. MALDI-TOF MS spectrum for **1·TAT**.

3 H_2O_2 cascade transformation

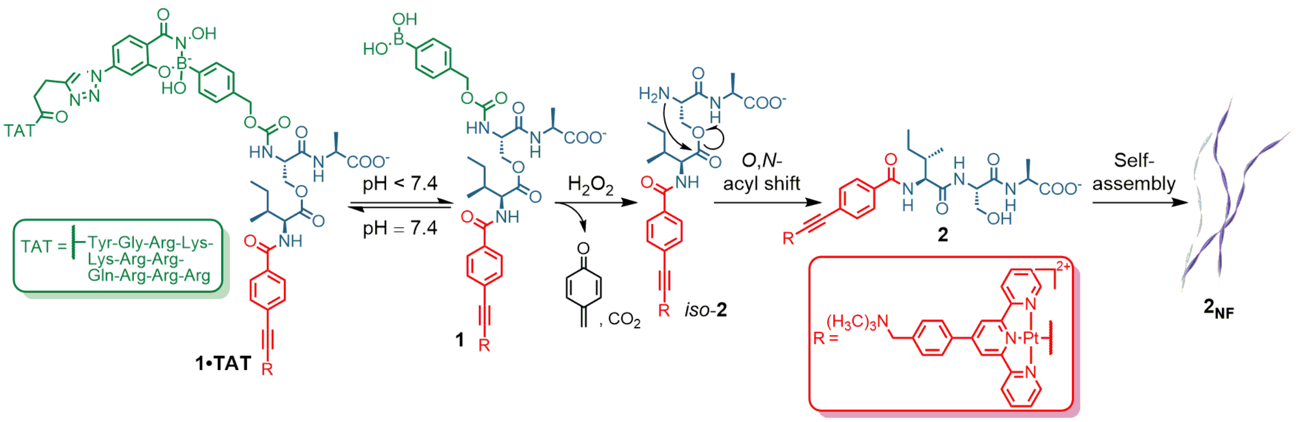

Figure S27. Full reaction scheme for conversion from **1·TAT** to **2**.

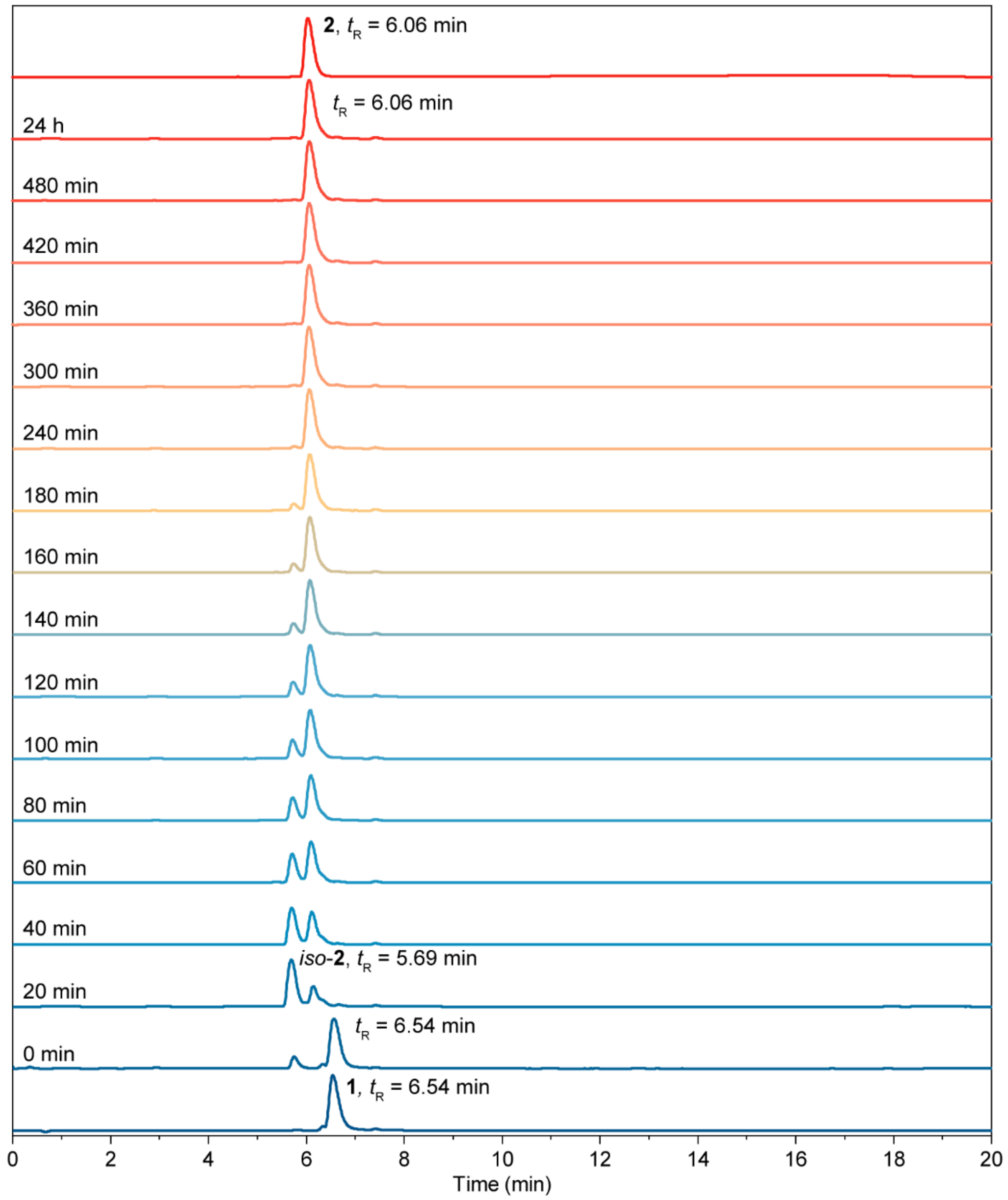

Figure S28. HPLC spectra for H_2O_2 (0.5 mM) induced-conversion of **1** (50 μM) to **2** in a mixture of NH_4HCO_3 buffer (pH 7.4, 20 mM) and methanol (*v*:*v* = 9:1).

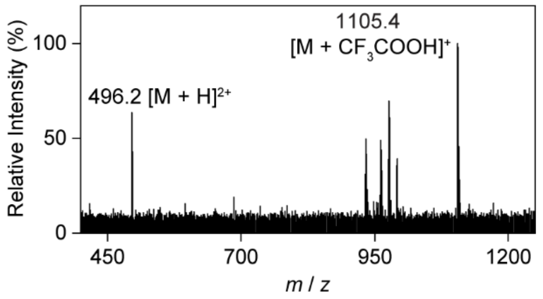

Figure S29. Convoluted MS spectrum for a reaction time of 20 min and t_R = 5.60-5.80 min (identified as *iso*-**2**).

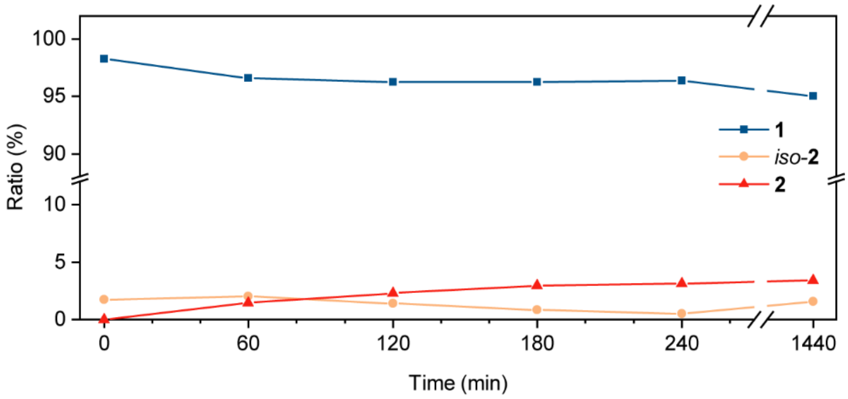

Figure S30. Molar ratio of compounds **1**, *iso*-**2** and **2** in LC-MS kinetic analysis when **1** (50 μM) was incubated in a mixture of NH_4HCO_3 buffer (pH 7.4, 20 mM) and methanol (*v*:*v* = 9:1).

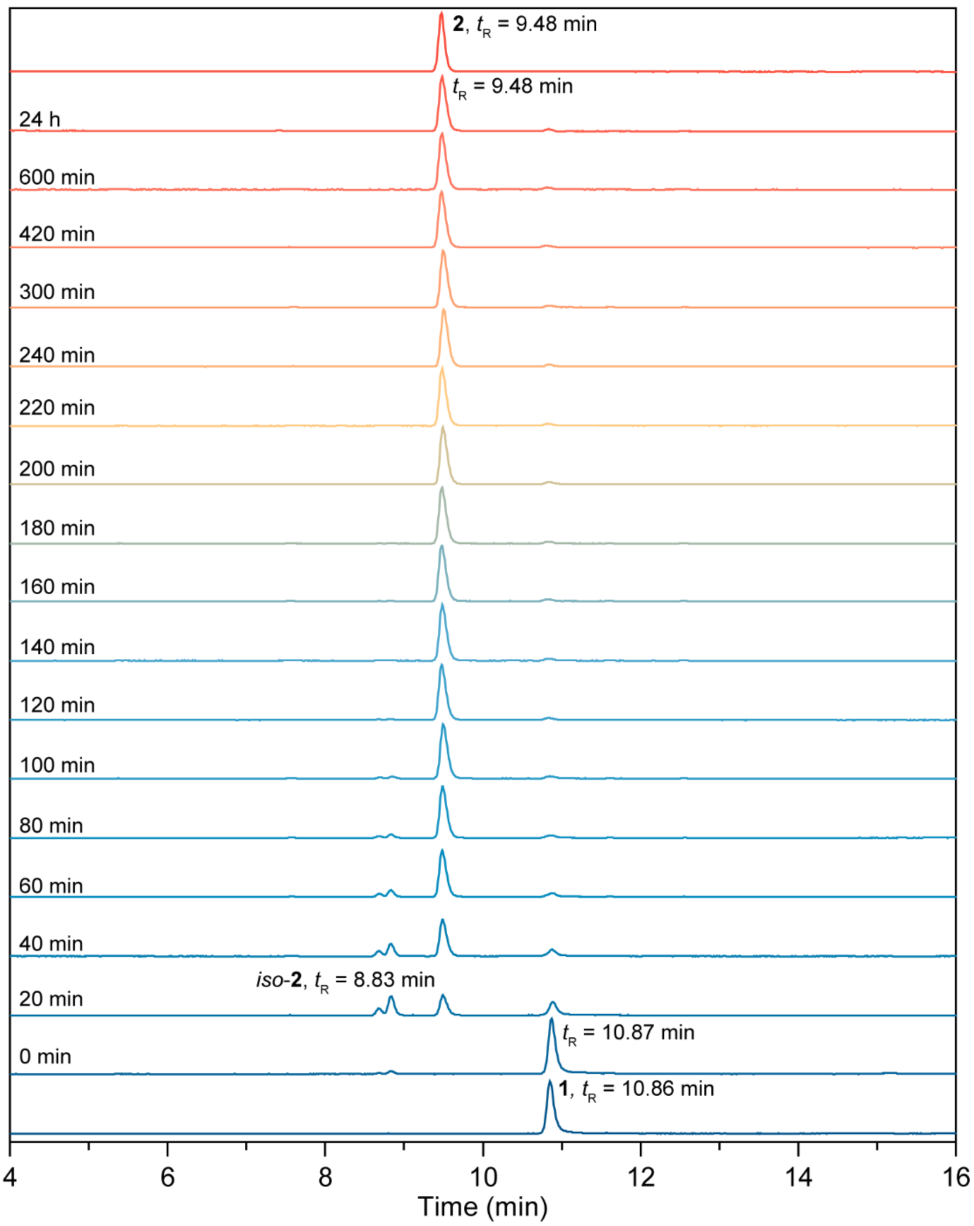

Figure S31. HPLC spectra for H_2O_2 (2.1 μM) induced-conversion of **1** (2.0 μM) to **2** in a mixture of NH_4HCO_3 buffer (pH 7.4, 20 mM) and methanol (*v*:*v* = 9:1). The retention times for the compounds are different from Figure 2B and S28 due the use of a different column.

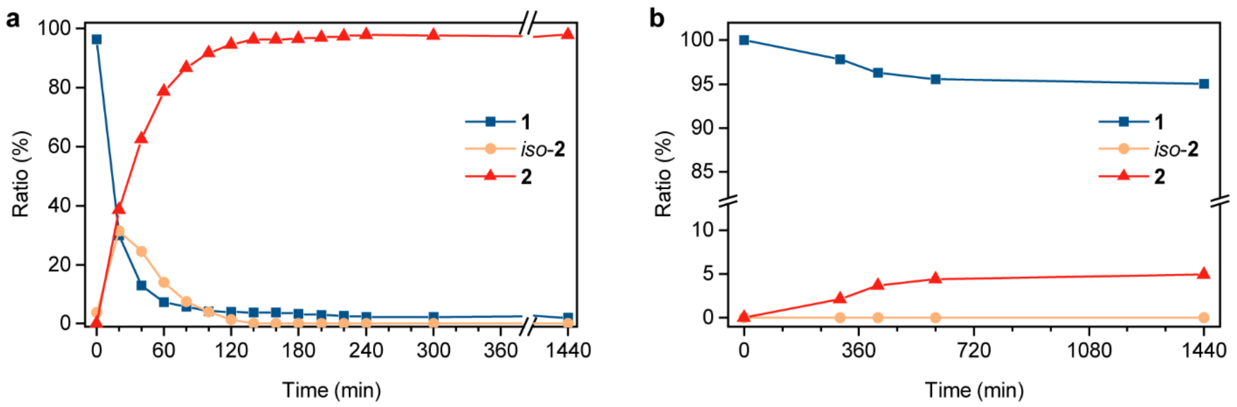

Figure S32. Molar ratio of compounds **1**, *iso*-**2** and **2** in LC-MS kinetic analysis when **1** (2.0 μM) was incubated in a mixture of NH_4HCO_3 buffer (pH 7.4, 20 mM) and methanol (*v*:*v* = 9:1) with (**a**) or without (**b**) H_2O_2 (2.1 μM).

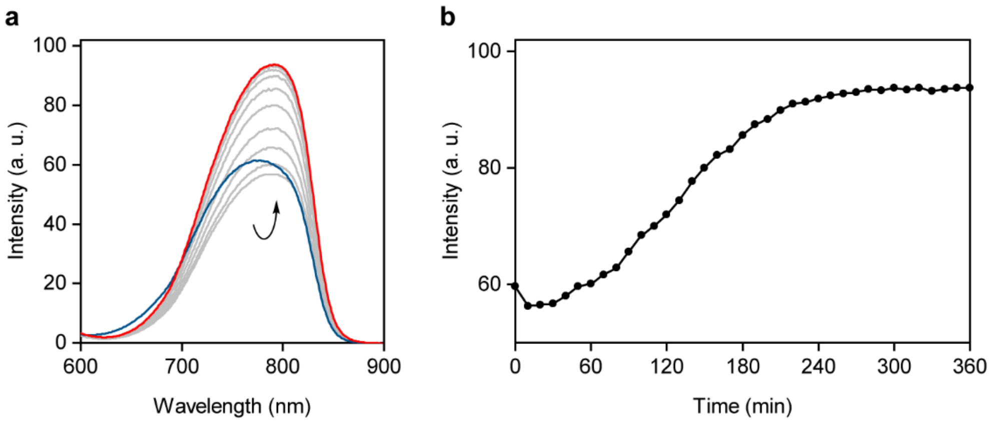

Figure S33. Luminescence analysis of H2O2-induced conversion from **1** to $\mathbf{2_{NF}}$. **a**, Time-dependent luminescence emission spectra of **1** (50 μM) in PB (pH 7.4, 50 mM) with H_2O_2 (0.5 mM). Blue line: incubation time = 0 min. Red line: incubation time = 300 min. Excitation wavelength = 488 nm. **b**, Time-dependent luminescence emission intensity for **1** (50 μM) in PB (pH 7.4, 50 mM) with H_2O_2 (0.5 mM) at 792 nm.

4 Self-assembly profiles of $\mathbf{2_{NF}}$.

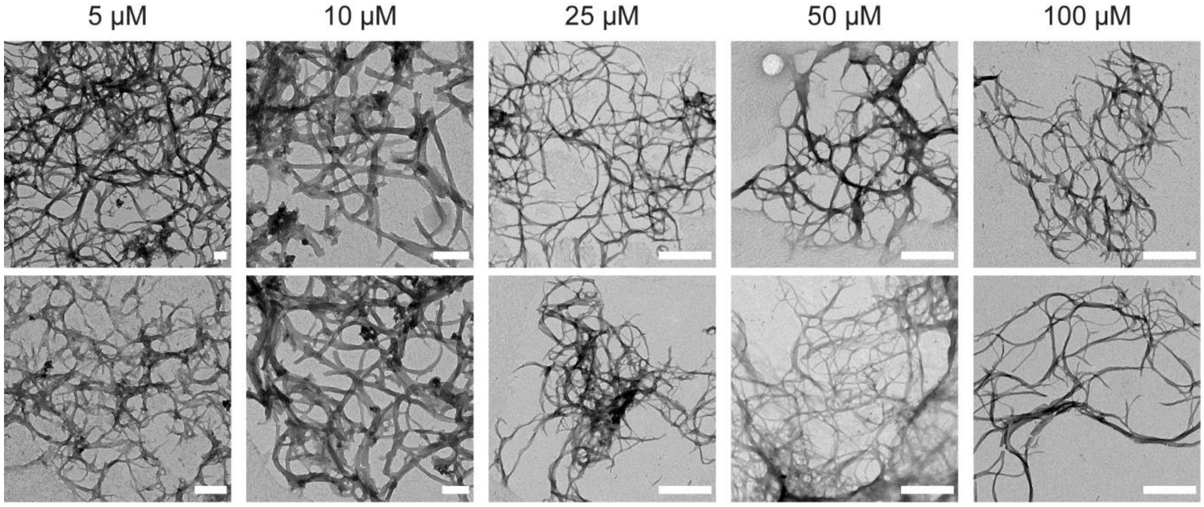

Figure S34. TEM image of the nanofiber $\mathbf{2_{NF}}$ at different concentrations in PB (pH 7.4, 50 mM). Scale bars, 200 nm.

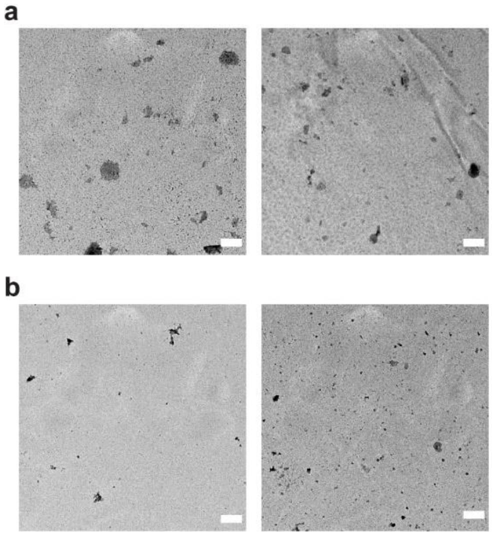

Figure S35. TEM image of **1** (a) and **1·TAT** (b) in PB (pH 7.4, 50 mM). Scale bars, 200 nm.

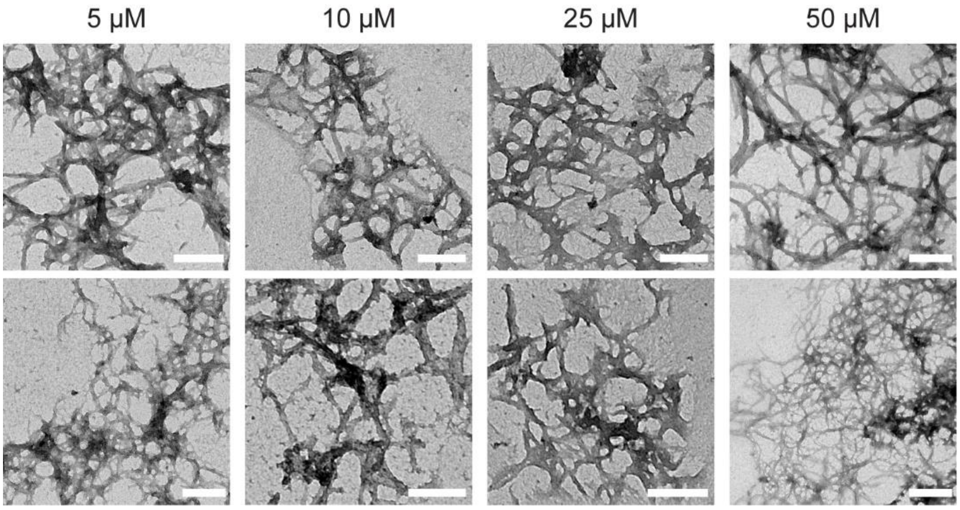

Figure S36. TEM image of the nanofiber form upon H_2O_2-induced conversion of **1·TAT**. Complex **1·TAT** and H_2O_2 (10 equiv) at various concentrations were incubated in PB (50 mM, pH 7.4) for 10 h. The reaction mixture was then drop-casted on a carbon grid and imaged using TEM. Scale bars, 200 nm.

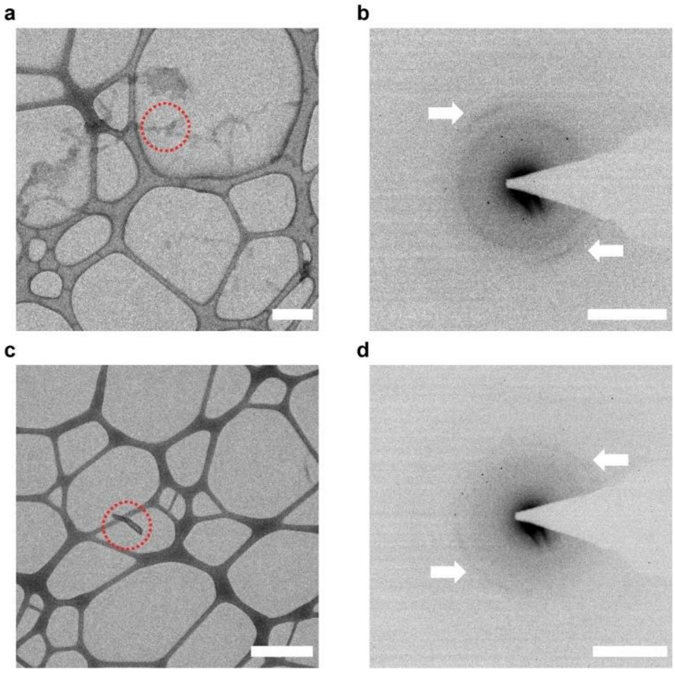

Figure S37. Selected-area electron diffraction analysis of the nanofiber. (**a**,**c**) Bright-field TEM images of the nanofiber. The red circles mark the position of the selected-area aperture. Scale bars, 500 nm. (**b**,**d**) Selected-area electron diffraction patterns obtained from the circular regions in (**a**) and (**c**), respectively. Scale bars, 3 nm^{-1}. White arrows indicate the diffraction arcs.

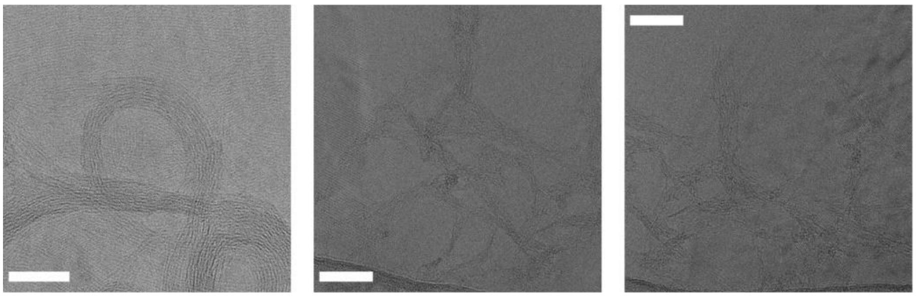

Figure S38. Cryogenic high-resolution TEM image of $\mathbf{2}_{\mathbf{NF}}$ in PB (pH 7.4, 50 mM). Scale bars, 50 nm.

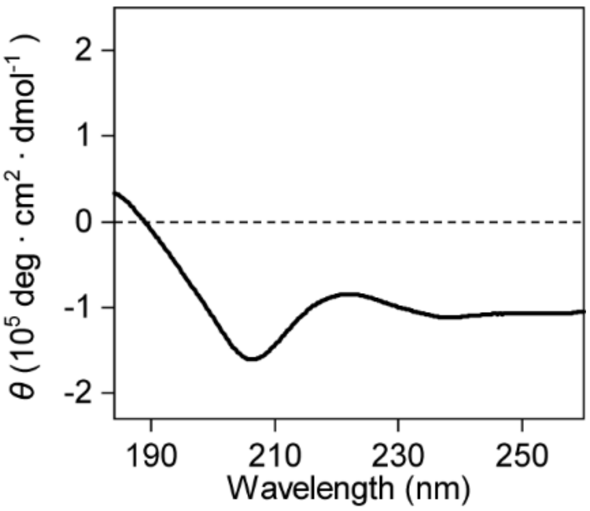

Figure S39. Circular dichroism (CD) spectra of **1** in a mixture of PB (pH 7.4, 10 mM) and CH_3CN (*v*:*v* = 98:2).

5 NMR analysis on the self-assembly profile of $\mathbf{2_{NF}}$.

At room temperature (298K), sharp, well-defined signal were observed for **2** (1 mg/mL) in the ^{1}H NMR spectrum, and increasing the temperature resulted in no significant change in the chemical shifts for the signals, except for the protons of the amide group (Figure S38), likely due to faster exchange with HDO in elevated temperature. These observations suggested that **2** remain as monomers in DMSO. In contrast, a solution of **2** at the same concentration in PB exhibited broad, overlapping signals at 298 K (Figure S39), in both aromatic and aliphatic region, suggesting strong self-assembly tendency of **2** in PB driven by a combination of π–π interactions and Pt-Pt interactions originating from the Pt-tpy group, and the hydrophobic interactions and hydrogen bond interactions originated from the peptide, forming nanofibers $\mathbf{2_{NF}}$. Increasing the temperature of the measurement led to sharpening of the signals, accompanied by downfield shifts, indicative of reduced shielding effect due to disassembly. To further investigate the self-assembly profile of **2** in PB, NMR studies were conducted at 353 K, at which the proton NMR signals are sharp and well-resolved.

^{1}H NMR was first conducted at 353K (Figure S42) and the peaks were assigned based on the ^{1}H NMR of **2** in DMSO, as well as ^{1}H,^{13}C HSQC NMR and ^{1}H, ^{1}H COSY NMR studies. In detail, the ^{1}H,^{13}C HSQC NMR spectrum of **2** in DMSO revealed carbon atoms proximity to the nitrogen atom of the tpy group showing highest chemical shifts in ^{13}C signals (Figure S43, 152.5 ppm for carbon directly bond to H^a and 141.2 ppm for carbon directly bond to H^c), whereas no other carbons that bond to a proton have chemical shifts above 135 ppm. Based on this observation, ^{1}H signals for **2** in PB can be identified using a combination of the ^{1}H,^{13}C HSQC spectrum (Figure S44, 152.3 ppm for carbon directly bond to H^a and 142.8 ppm for carbon directly bond to H^c, no other carbons that bond to a proton have chemical shifts above 135 ppm) and ^{1}H, ^{1}H COSY NMR spectrum (Figures S45 and S46, showing correlations between neighboring protons).

^{1}H,^{1}H NOESY NMR spectrum for **2** in PB was then collected (Figure S47) to probe signals arise from protons that are close to each other in space even if they are not bonded, hence providing information on the self-assembly character. Similar to the COSY study discussed previously, strong off-diagonal

cross peaks can be observed for bonded neighboring protons. In addition, five sets of cross peaks from non-neighboring protons, H^a/H^c, H^d/H^h H^e/H^h, H^b/H^d, and H^b/H^e were identified. These signals suggest that **2** adopt a head-to-tail conformation upon self-assembly in PB, forming **2**$_{\mathbf{NF}}$.

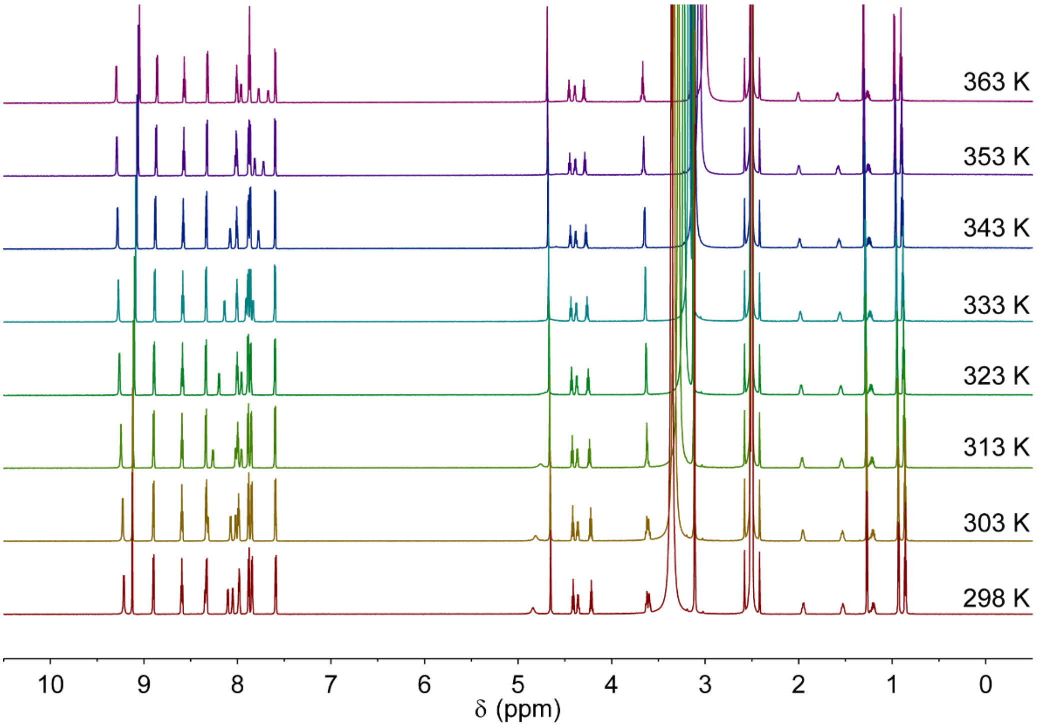

Figure S40. ^{1}H NMR spectra (850 MHz, DMSO-d_6) of compound **2** at various temperature.

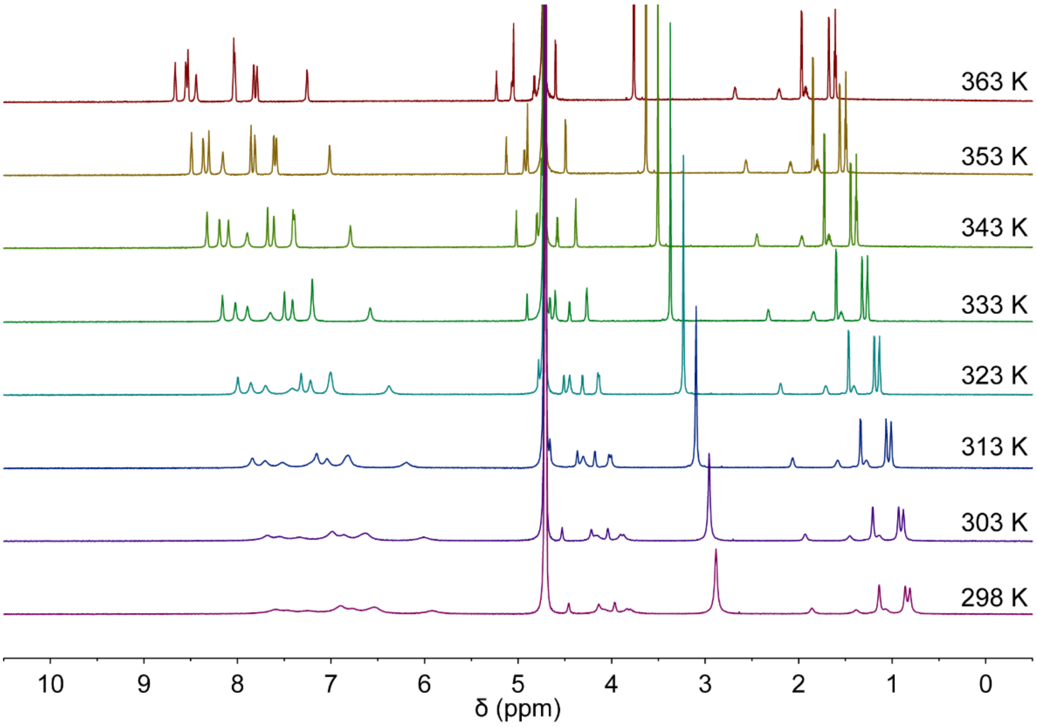

Figure S41. ^{1}H NMR spectra [850 MHz, PB (pH 7.4, 50 mM) in D_2O] of compound **2** at various temperature.

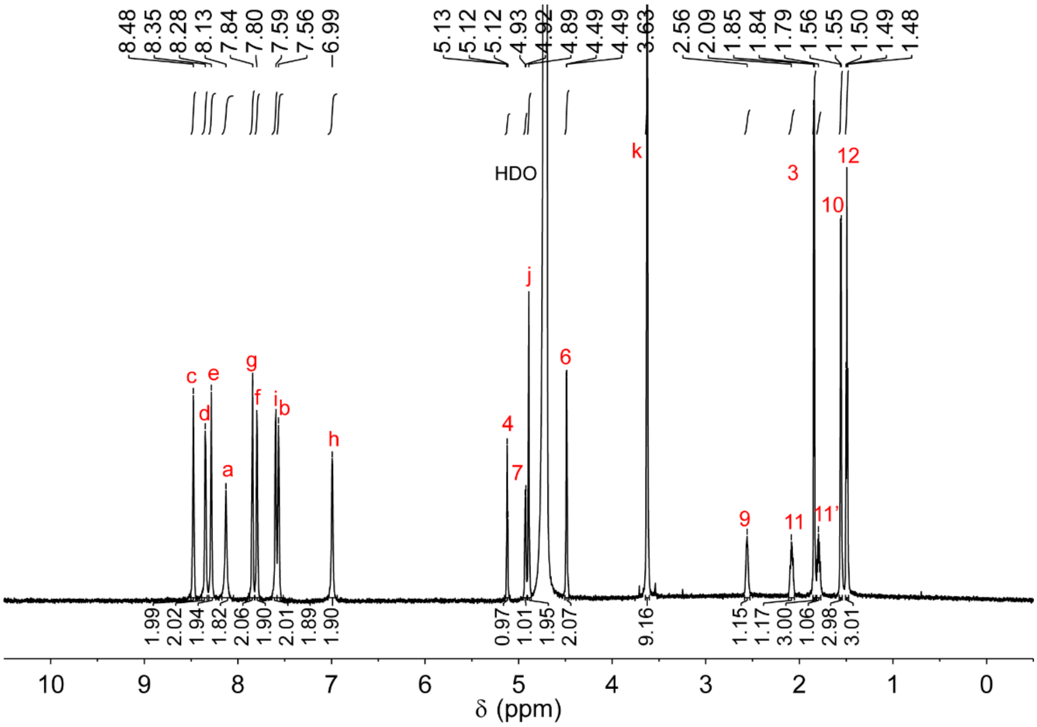

Figure S42. ^{1}H NMR spectrum [850 MHz, PB (pH 7.4, 50 mM) in D_2O, 353K] of compound **2**.

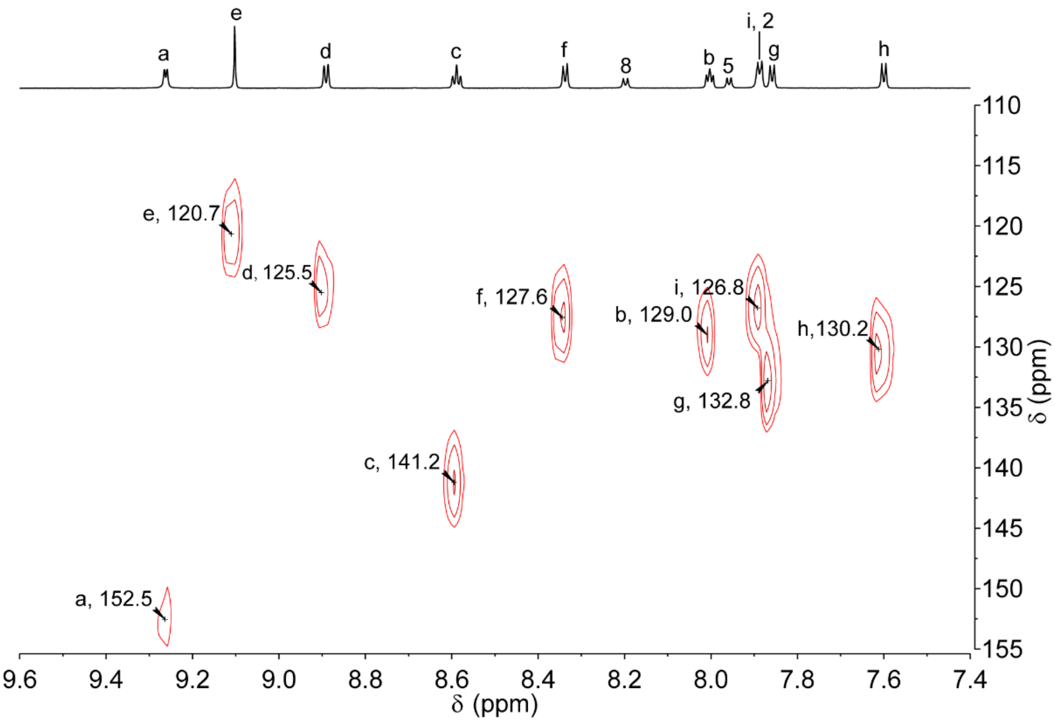

Figure S43. ^{1}H,^{13}C HSQC NMR spectra (850 MHz, DMSO-d_6, 298K) of compound **2**.

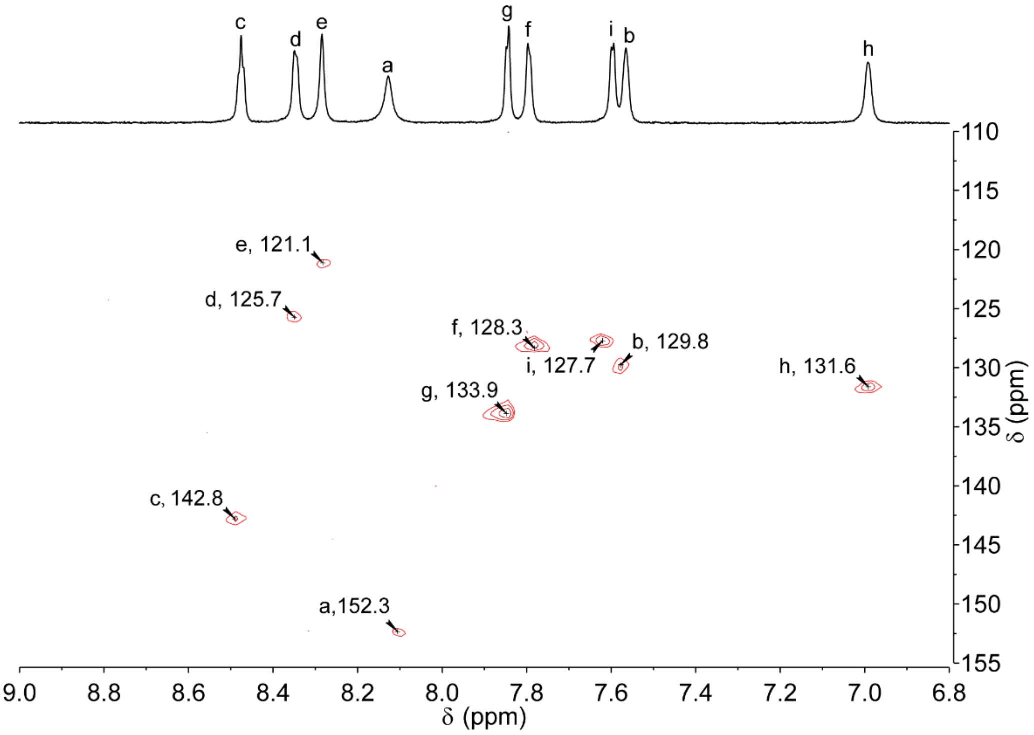

Figure S44. $^{1}H,^{13}C$ HSQC NMR spectra [850 MHz, PB (pH 7.4, 50 mM) in D_2O, 353K] of compound **2**.

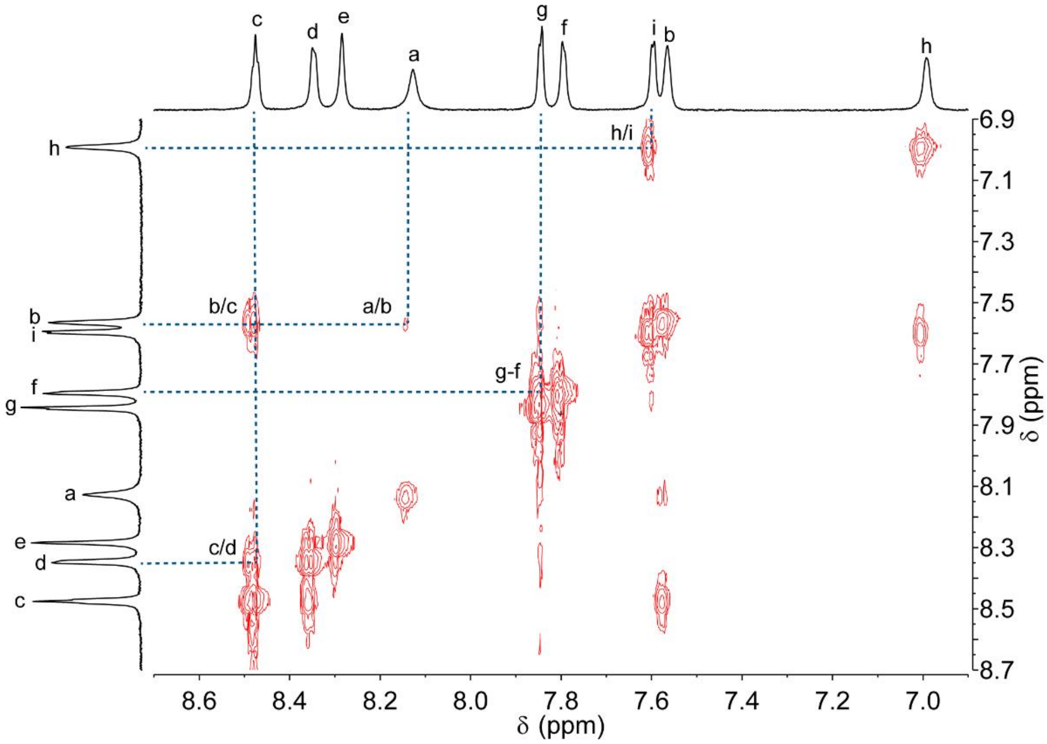

Figure S45. Partial ^{1}H NMR and $^{1}H,^{1}H$ COSY NMR spectra [850 MHz, PB (pH 7.4, 50 mM) in D_2O, 353K] of compound **2** (aromatic region). The correlations between ^{1}H signals are highlighted with dashed lines in the COSY spectrum.

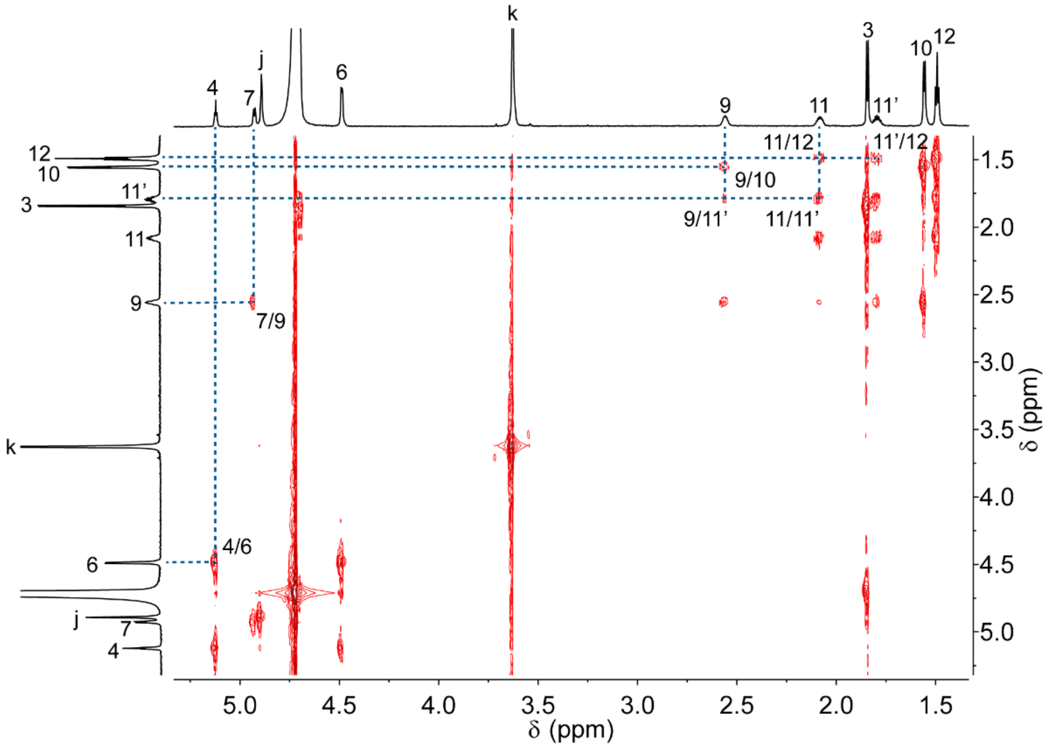

Figure S46. Partial ^{1}H NMR and $^{1}H,^{1}H$ COSY NMR spectra [850 MHz, PB (pH 7.4, 50 mM) in D_2O, 353K] of compound **2** (aliphatic region). The correlations between ^{1}H signals are highlighted with dashed lines in the COSY spectrum.

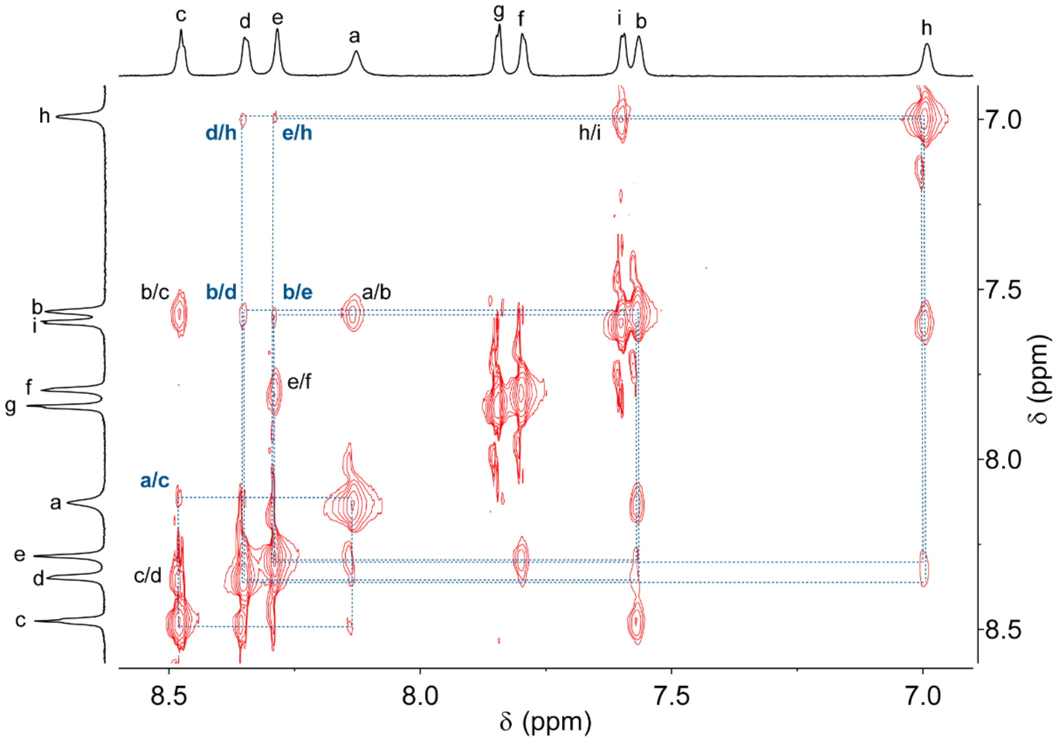

Figure S47. $^{1}H,^{13}H$ NOESY NMR spectrum [850 MHz, PB (pH 7.4, 50 mM) in D_2O, 353K] of compound **2**. Off-diagonal cross peaks that corresponds to intermolecular NOE signals were highlighted in blue.

6 Cellular uptake and intracellular self-assembly

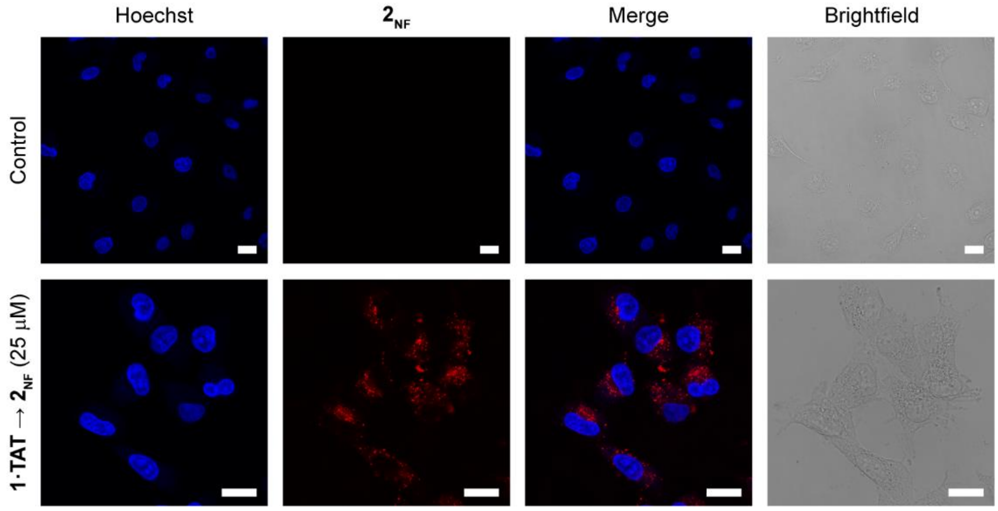

Figure S48. Confocal laser scanning micrographs of A549 cells treated for 4 h with **1·TAT** (25 μM) and Hoechst 33342 dye. Scale bars, 20 μm.

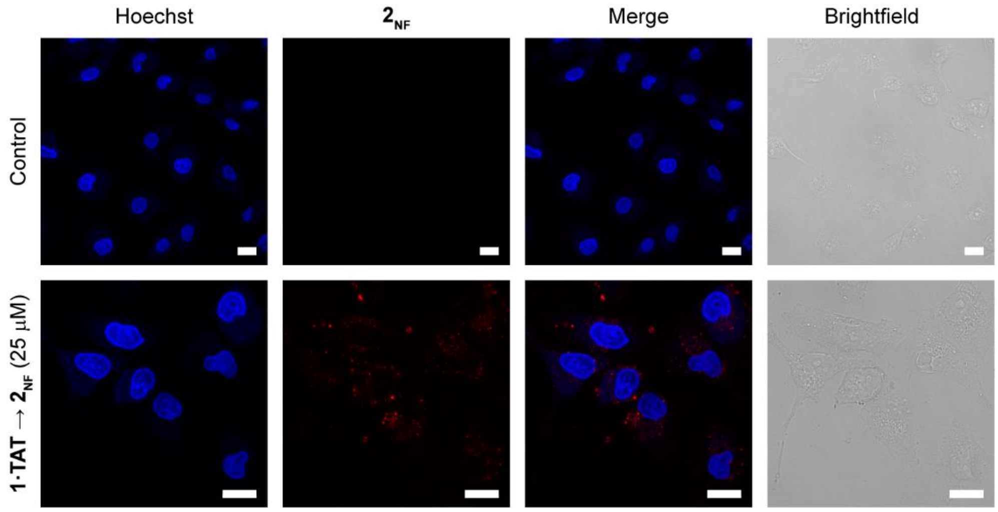

Figure S49. Confocal laser scanning micrographs of MDA-MB-231 cells treated for 4 h with **1·TAT** (25 μM) and Hoechst 33342 dye. Scale bars, 20 μm.

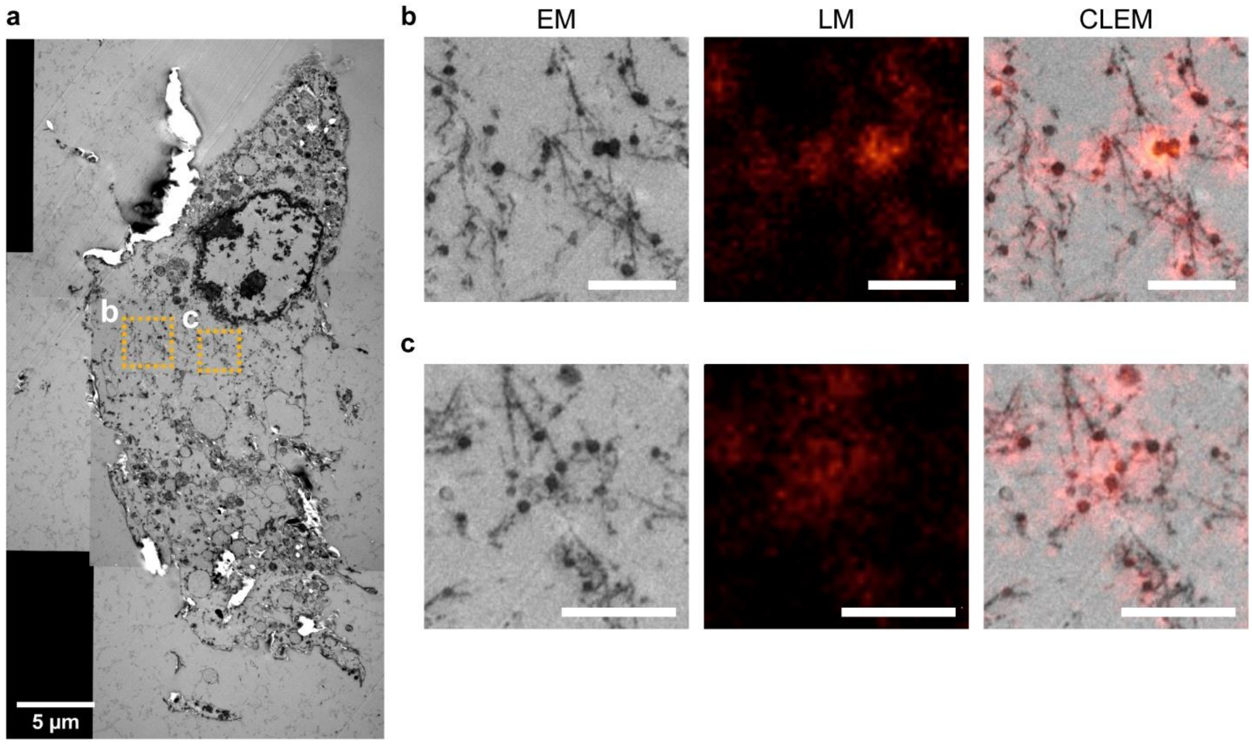

Figure S50. CLEM analysis of A549 cells treated with **1·TAT** (50 μM, 6 h). **a**, Stitched TEM image of an A549 cell showing intracellular peptide fiber formation. Scale bars, 5 μM. **b-c**, TEM, LM, and CLEM image of the box area showing the intracellular formation of luminescent nanofibers. Scale bars, 1 μm.

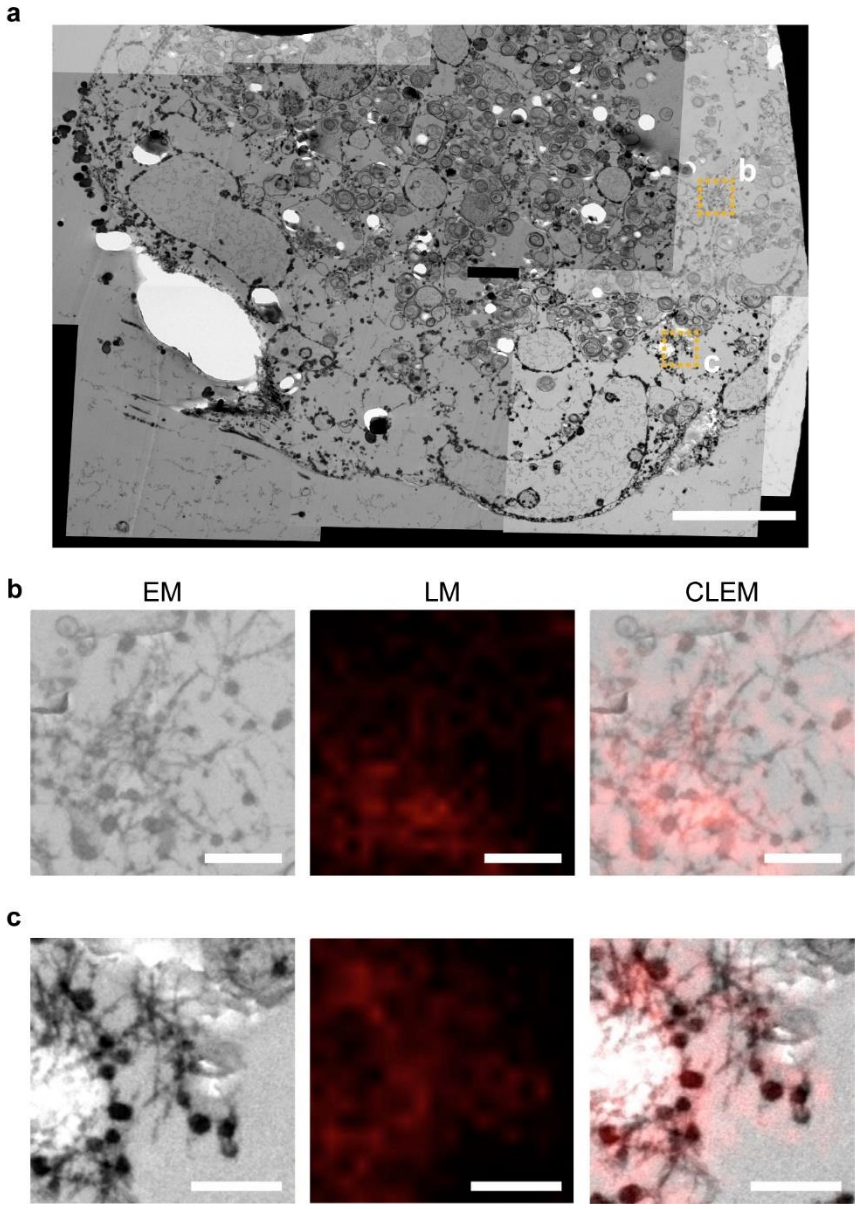

Figure S51. CLEM analysis of A549 cells treated with **1·TAT** (50 μM, 6 h). **a**, Stitched TEM image of an A549 cell showing intracellular peptide fiber formation. Scale bars, 1 μm. **b**-**c**, TEM, LM, and CLEM image of the box area showing intracellular formation of luminescent nanofibers. Scale bars, 500 nm.

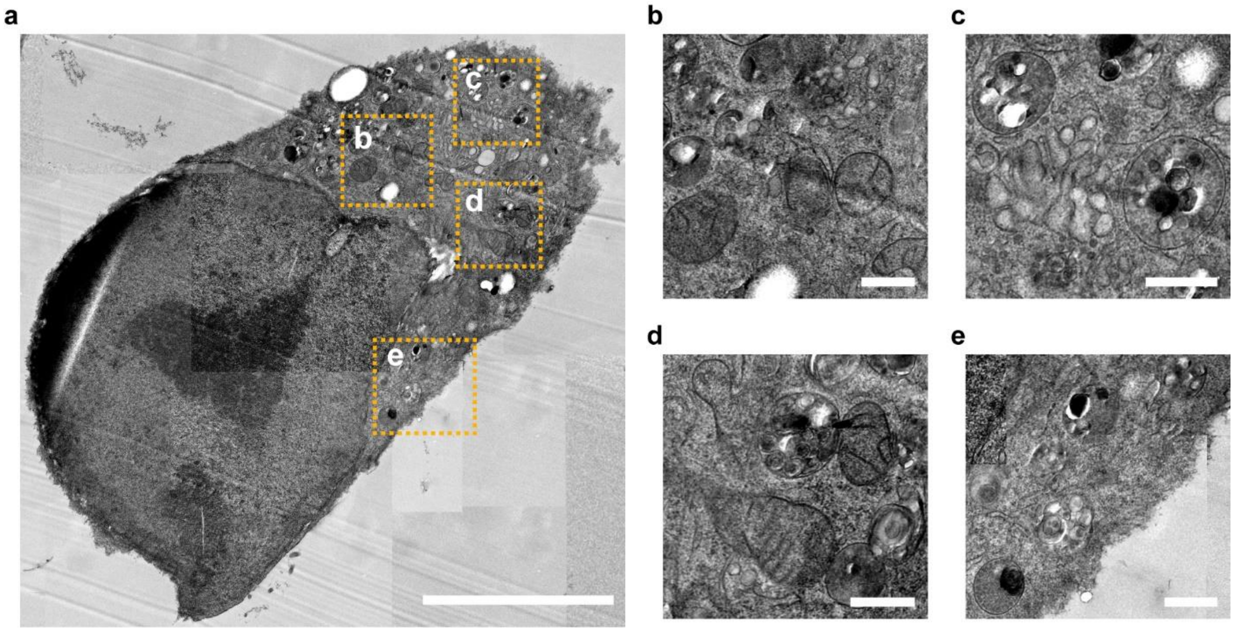

Figure S52. TEM image of a control A549 cell that was not treated with **1·TAT** after stitching TEM micrographs. **a**, Full view of the A549 cell after stitching TEM micrographs. Scale bars, 5 μm. **b**-**e**, Zoom in TEM images of the A594 cells. Scale bars, 500 nm. No nanofibers were observed in the control cells.

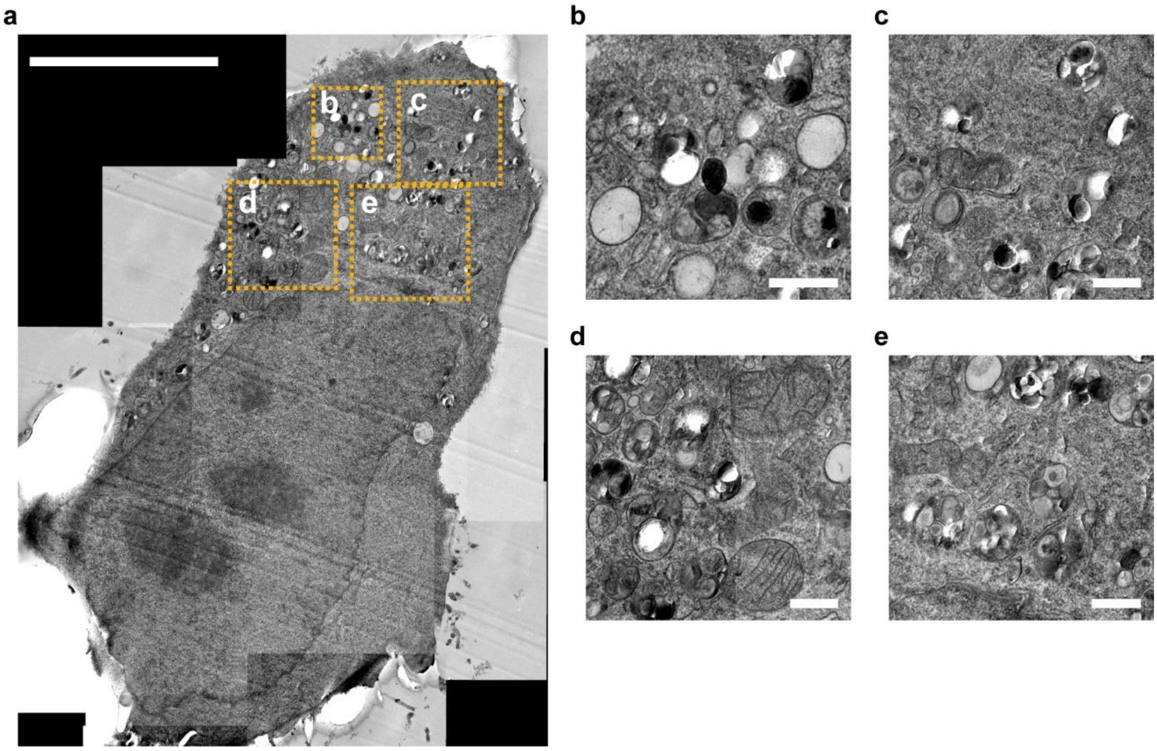

Figure S53. TEM image of a control A549 cell that was not treated with **1·TAT** after stitching TEM micrographs. **a**, Full view of the A549 cell after stitching TEM micrographs. Scale bars, 5 μm. **b**-**e**, Zoom in TEM images of the A594 cells. Scale bars, 500 nm. No nanofibers were observed in the control cells.

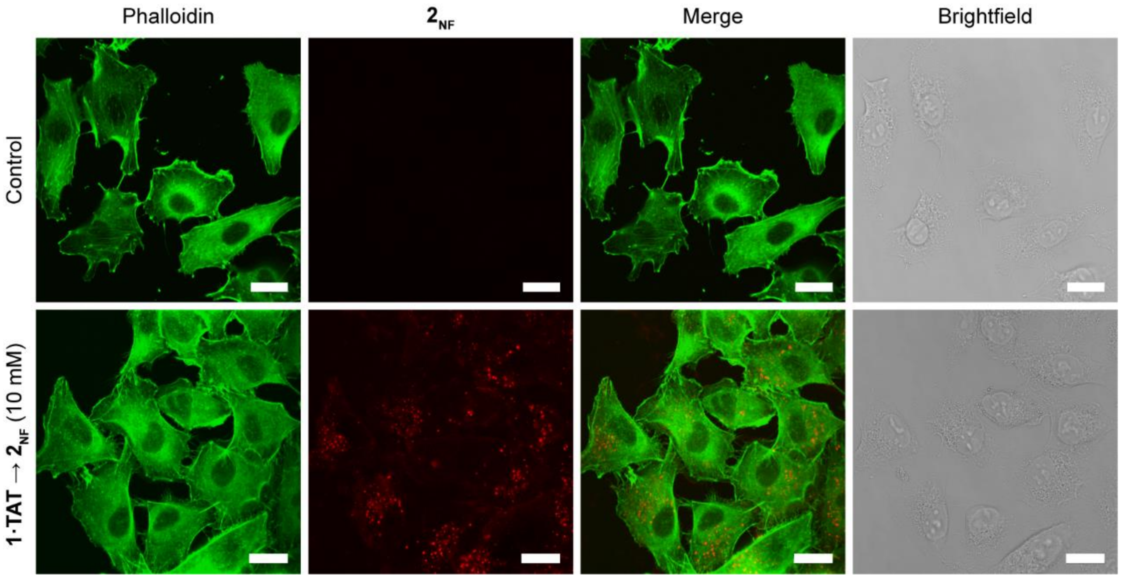

Figure S54. Confocal laser scanning micrographs of A549 cells treated for 4 h with **1·TAT** (10 μM) and Phalloidin-iFluor™ 405 dye. Scale bars, 20 μm.

7 Metabolic inhibitions by the 1·TAT→2_{NF}

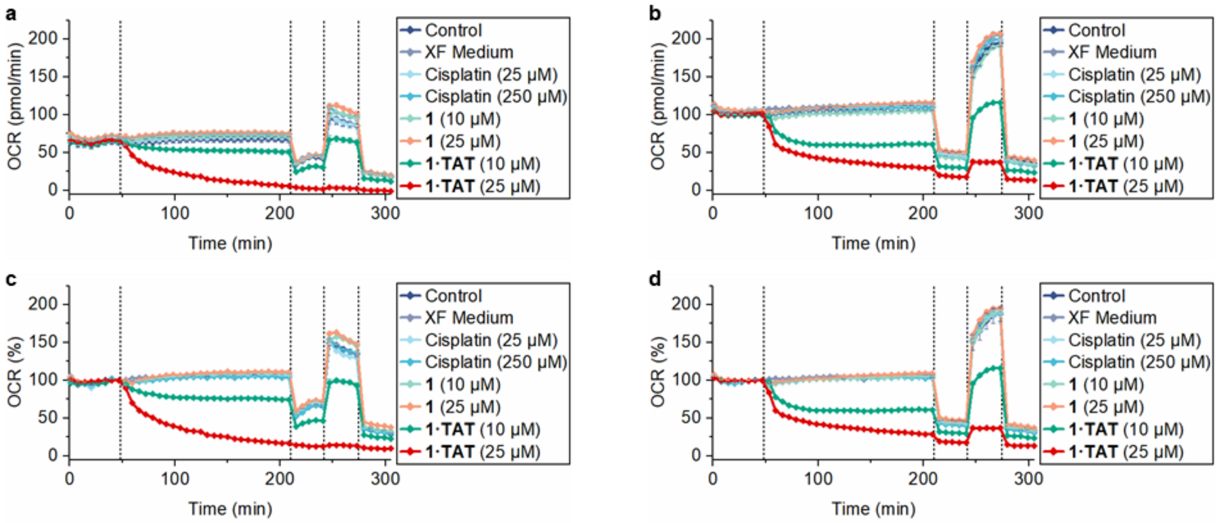

Figure S55. Results of the Mito Stress Assay for A549 and MDA-MB-231 cells. Graphs are shown for the raw data and after setting the last rate measurement before sample addition to 100% baseline. Dashed vertical lines indicate the time points of compound addition. The first time point indicates the addition of sample, followed by oligomycin, FCCP and lastly Rotenone and Antimycin A as a combination. **a** and **c** show the data before and after baseline correction respectively, for A549 cells. **b** and **d** show the data before and after baseline correction respectively, for MDA-MB-231 cells.

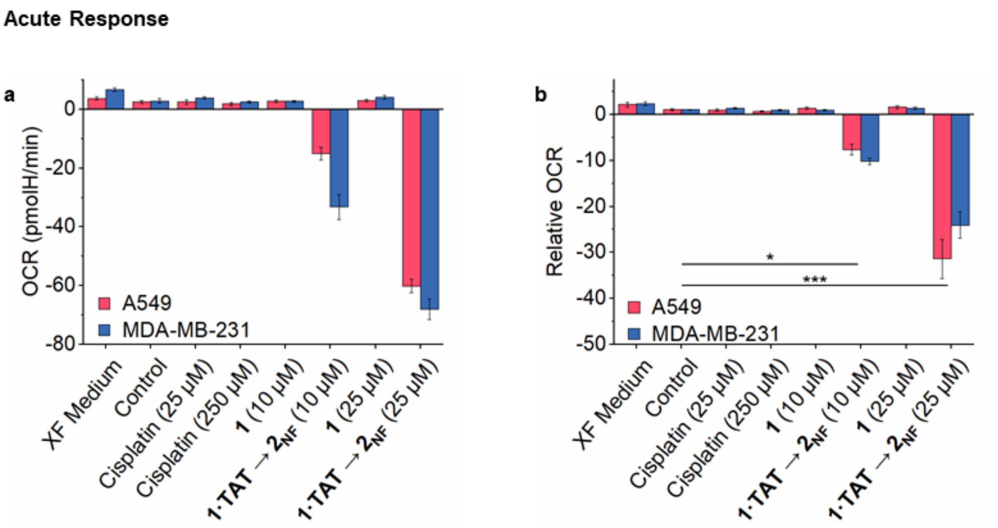

Figure S56. Acute Response of A549 and MDA-MB-231 cells during the Mito Stress Assay upon treatment with **1, 1·TAT** as well as XF Medium and Cisplatin controls. Data are shown for the raw data (**a**) and for a baseline set to 100% before the sample addition (**b**). In the latter case, each cell line was normalized to the DMSO control. (**b**) n≥6.

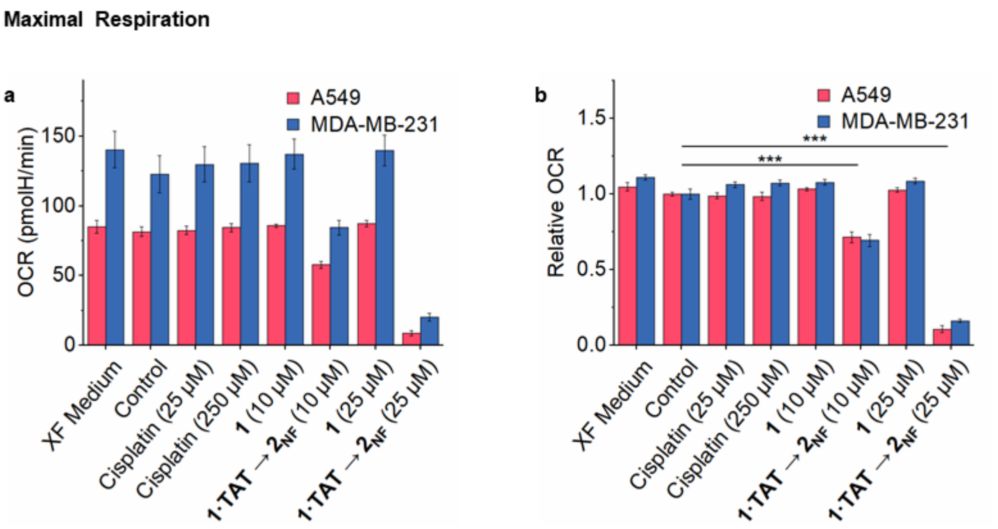

Figure S57. Maximal Respiratory Capacity of A549 and MDA-MB-231 cells during the Mito Stress Assay upon treatment with **1, 1·TAT** as well as XF Medium and Cisplatin controls. Data are shown for the raw data (**a**) and for a baseline set to 100% before the sample addition (**b**). In the latter case, each cell line was normalized to the DMSO control. (**b**) n≥6.

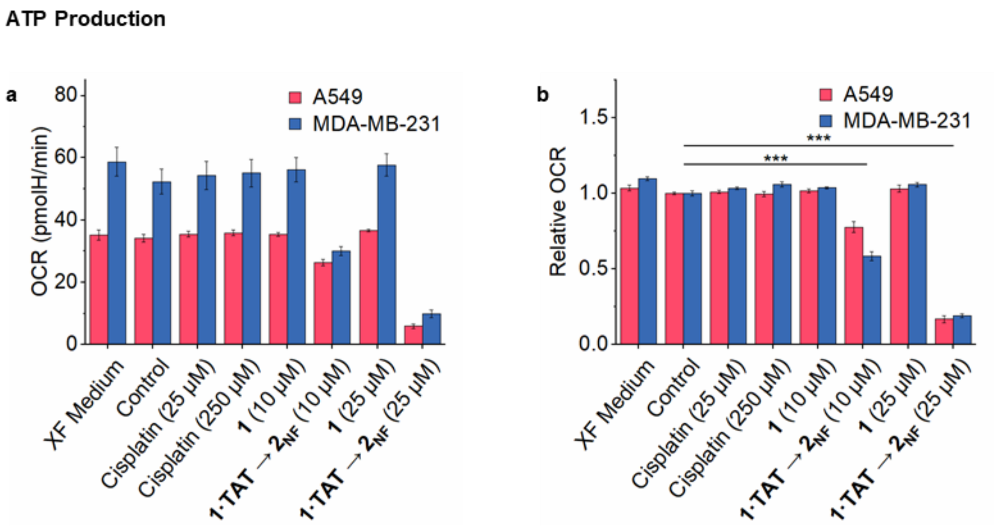

Figure S58. ATP Production of A549 and MDA-MB-231 cells during the Mito Stress Assay upon treatment with **1, 1·TAT** as well as XF Medium and Cisplatin controls. Data are shown for the raw data (**a**) and for a baseline set to 100% before the sample addition (**b**). In the latter case, each cell line was normalized to the DMSO control. (**b**) n≥6.

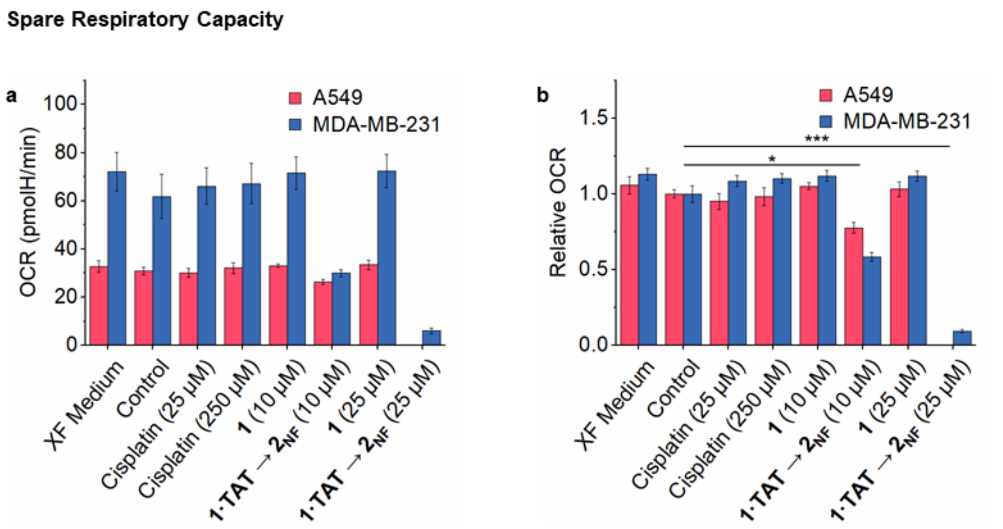

Figure S59. Spare Respiratory Capacity of A549 and MDA-MB-231 cells during the Mito Stress Assay upon treatment with **1, 1·TAT** as well as XF Medium and Cisplatin controls. Data are shown for the raw data (**a**) and for a baseline set to 100% before the sample addition (**b**). In the latter case, each cell line was normalized to the DMSO control. (**b**) n≥6.

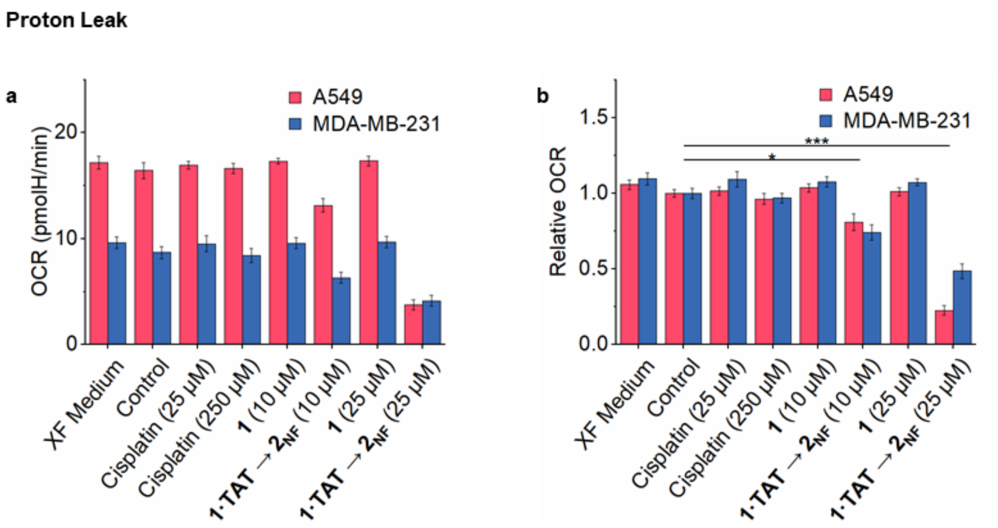

Figure S60. Proton Leak of A549 and MDA-MB-231 cells during the Mito Stress Assay upon treatment with **1, 1·TAT** as well as XF Medium and Cisplatin controls. Data are shown for the raw data (**a**) and for a baseline set to 100% before the sample addition (**b**). In the latter case, each cell line was normalized to the DMSO control. (**b**) n≥6.

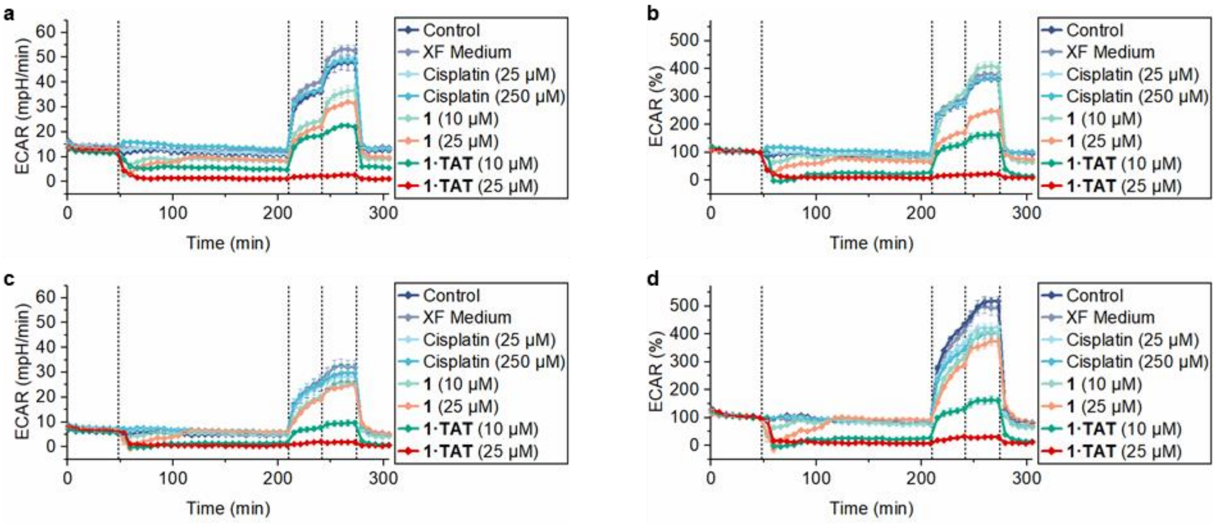

Figure S61. Results of the Glycolysis Stress Assay for A549 and MDA-MB-231 cells. Graphs are shown for the raw data and after setting the last rate measurement before sample addition to 100% baseline. Dashed vertical lines indicate the time points of compound addition. The first time point indicates the addition of the sample, followed by glucose, oligomycin and 2-deoxyglucose respectively. **a** and **c** show the data before and after baseline correction respectively, for A549 cells. **b** and **d** show the data before and after baseline correction respectively, for MDA-MB-231 cells.

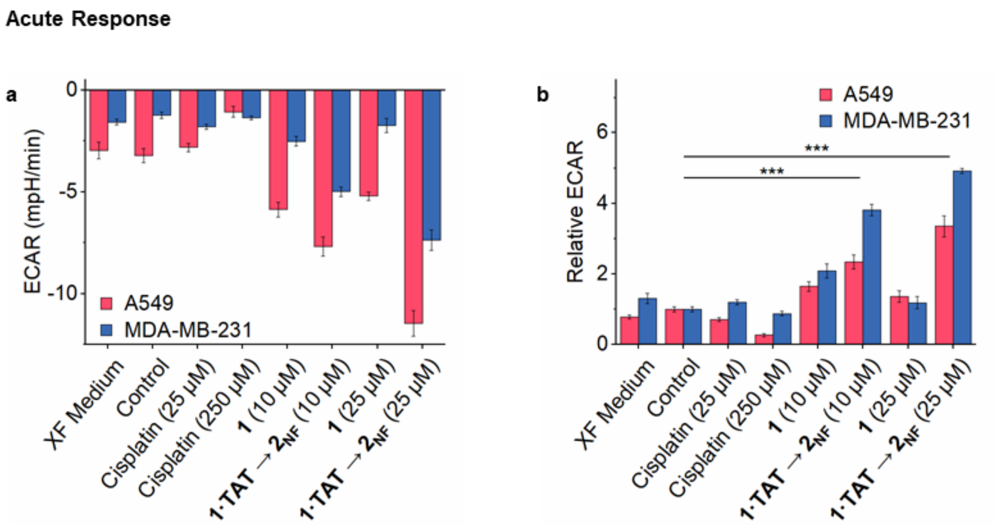

Figure S62. Acute Response of A549 and MDA-MB-231 cells during the Glycolysis Stress Assay upon treatment with **1, 1·TAT** as well as XF Medium and Cisplatin controls. Data are shown for the raw data (**a**) and for a baseline set to 100% before the sample addition (**b**). In the latter case, each cell line was normalized to the DMSO control. (**b**) n≥6.

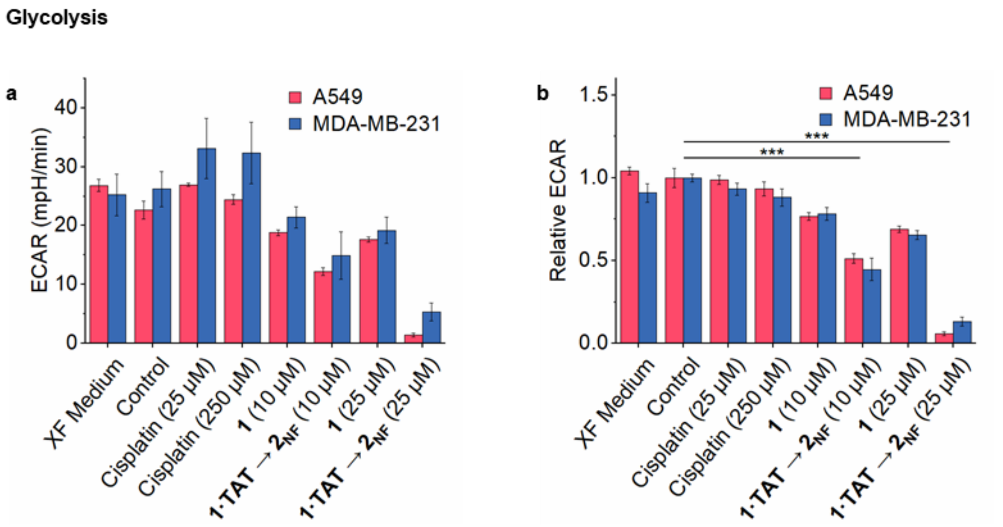

Figure S63. Glycolysis of A549 and MDA-MB-231 cells during the Glycolysis Stress Assay upon treatment with **1, 1·TAT** as well as XF Medium and Cisplatin controls. Data are shown for the raw data (**a**) and for a baseline set to 100% before the sample addition (**b**). In the latter case, each cell line was normalized to the DMSO control. (**b**) n≥6.

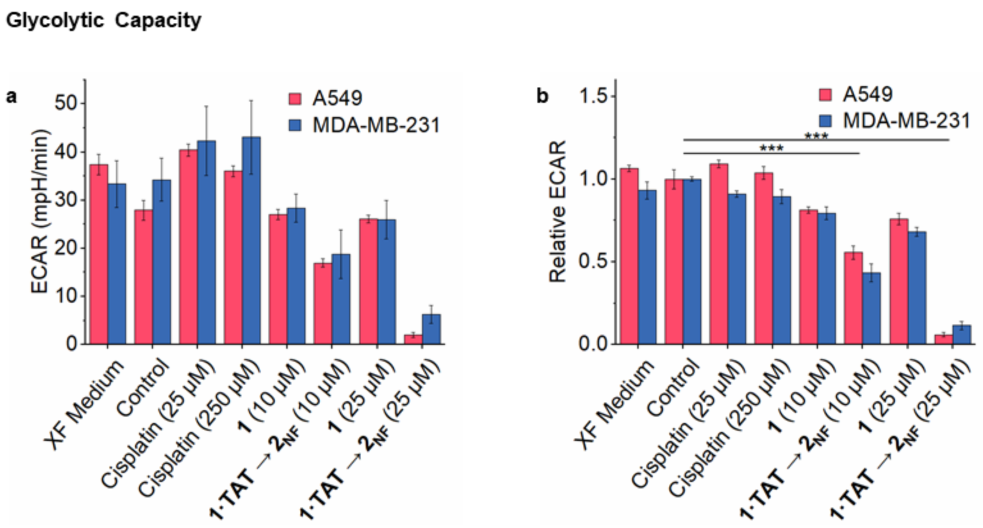

Figure S64. Glycolytic Capacity of A549 and MDA-MB-231 cells during the Glycolysis Stress Assay upon treatment with **1, 1·TAT** as well as XF Medium and Cisplatin controls. Data are shown for the raw data (**a**) and for a baseline set to 100% before the sample addition (**b**). In the latter case, each cell line was normalized to the DMSO control. (**b**) n≥5.

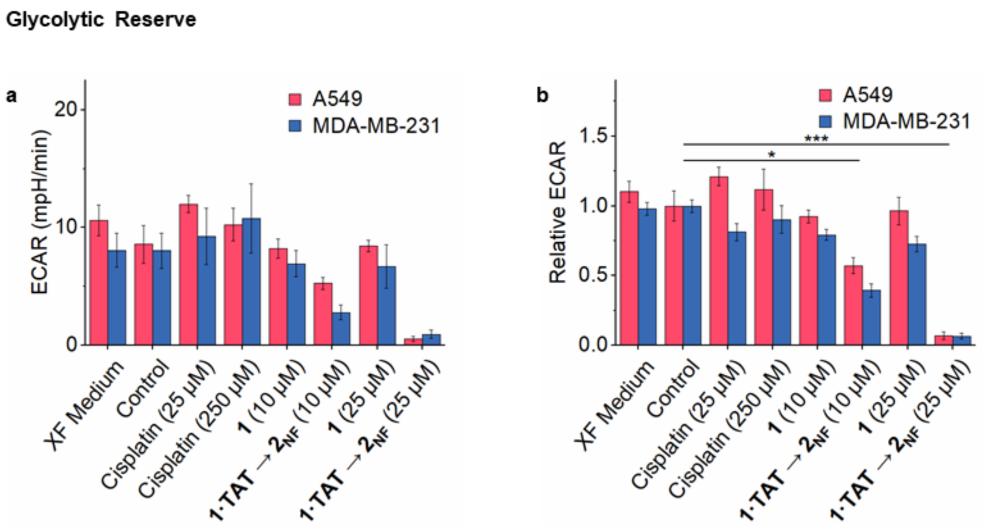

Figure S65. Glycolytic Reserve of A549 and MDA-MB-231 cells during the Glycolysis Stress Assay upon treatment with **1, 1·TAT** as well as XF Medium and Cisplatin controls. Data are shown for the raw data (**a**) and for a baseline set to 100% before the sample addition (**b**). In the latter case, each cell line was normalized to the DMSO control. (**b**) n≥5.

8 Cytotoxicity of 1·TAT→2_{NF}

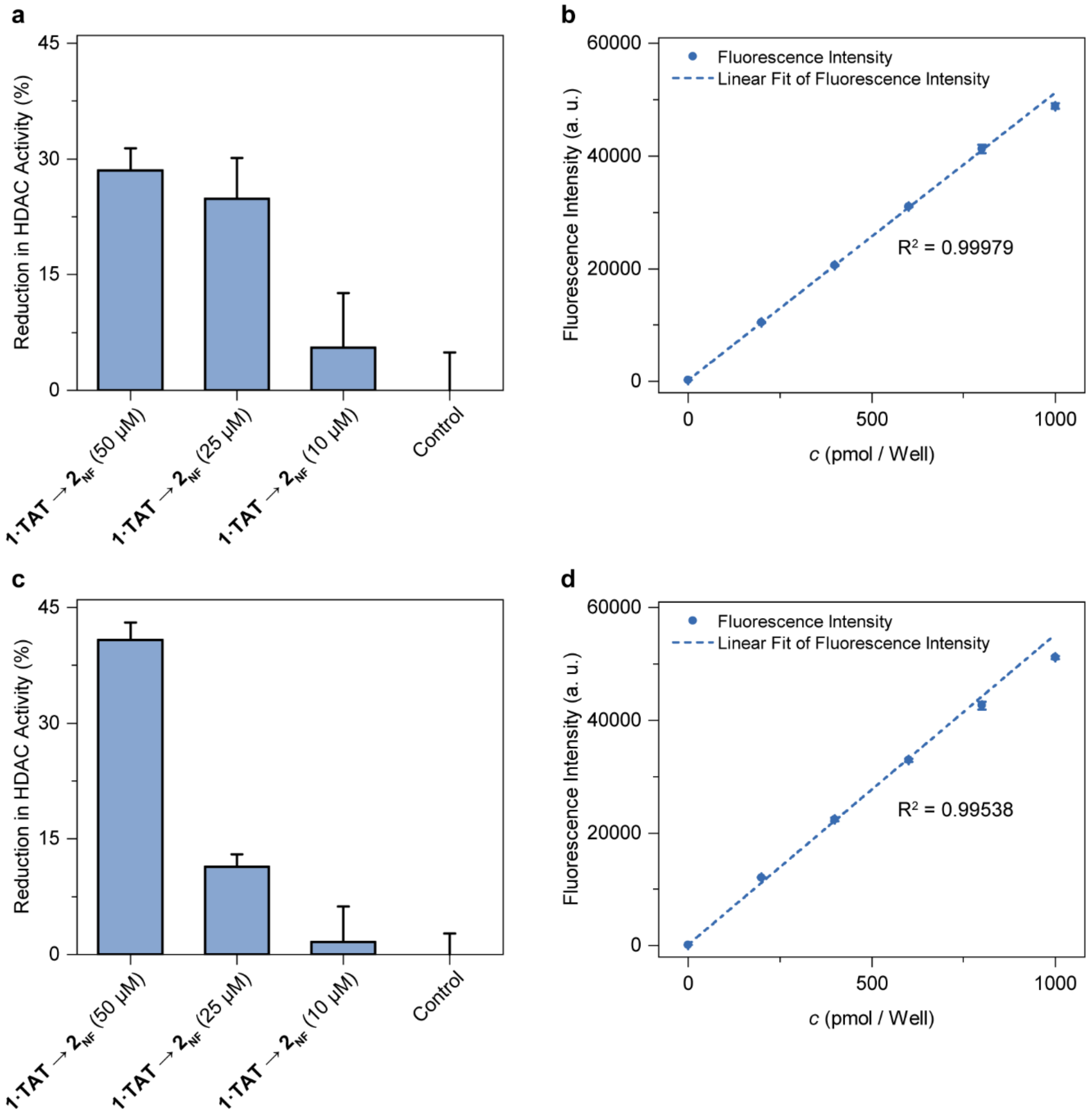

Figure S66. Examination of cellular histone deacetylase (HDAC) activity after incubation with **1·TAT** for 4 h**. a**, Reaction in HDAC activity by **1·TAT** at various concentrations against A549 cells. **b**, Calibration curve for the assay in **a**. **c**, Reaction in HDAC activity by **1·TAT** at various concentrations against MDA-MD-231 cells. **d**, Calibration curve for the assay in **c**.

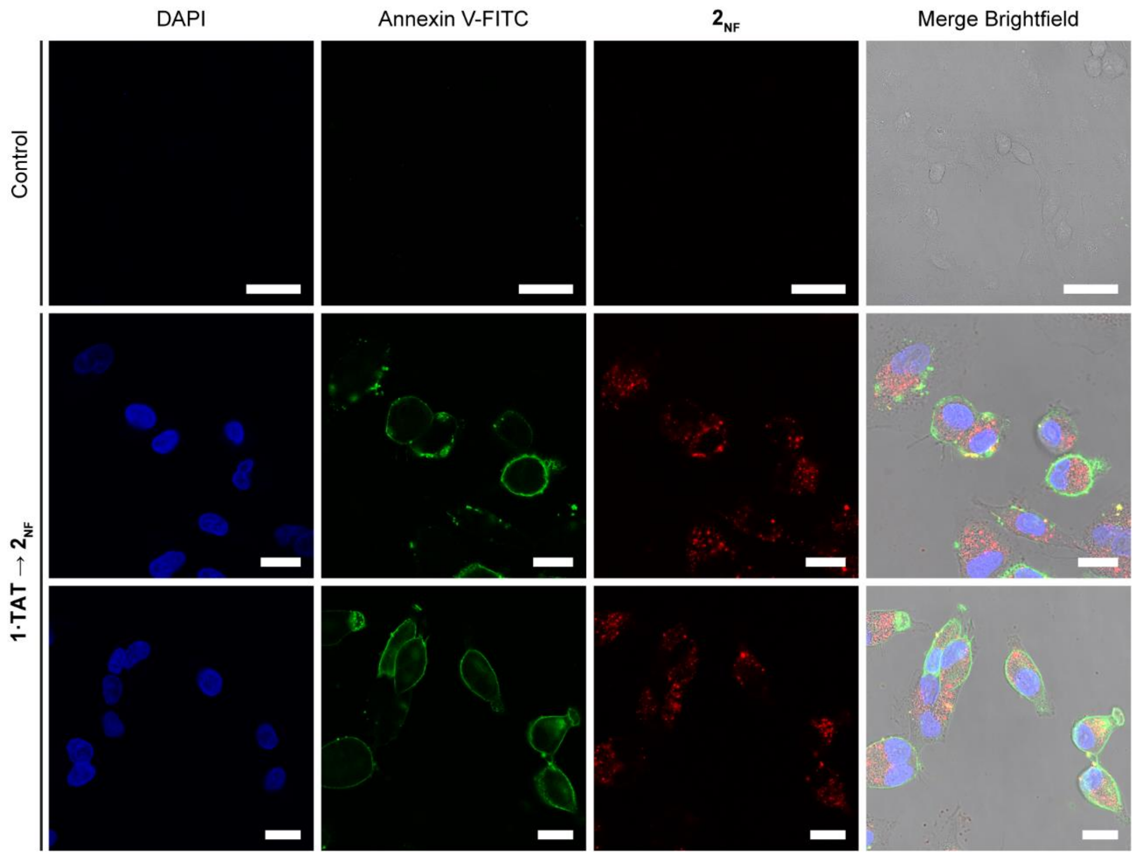

Figure S67. Examination of the apoptotic nature of cell death by using Annexin V assay. Cells were incubated 4 h with **1·TAT** (25 μM) (red) and treated with Annexin V (green) afterwards. Nuclei were stained using DAPI (blue). Annexin V binds to the cell membrane upon an inversion of phosphatidylserine motifs towards the extracellular space, indicating apoptosis. Each panel represent results from replicate of the experiment. Scale bars, 50 μm.

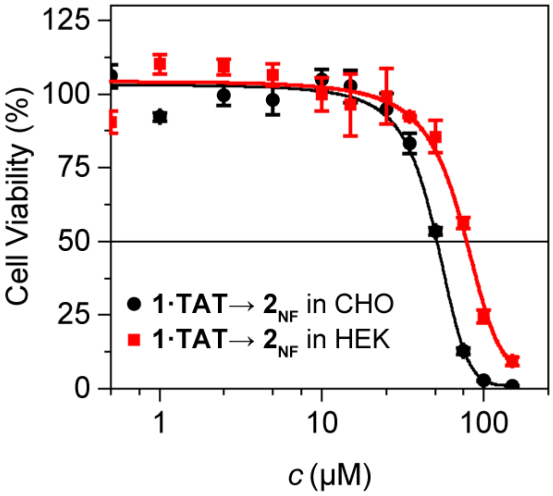

Figure S68. Cell viability assay of CHO and MDA-MB-231 cells treated with **1·TAT** and cisplatin for 4 h. The IC_{50} values for **1·TAT→2$_{NF}$** are determined to be 49.8 μM and 72.3 μM against CHO and HEK cells, respectively.

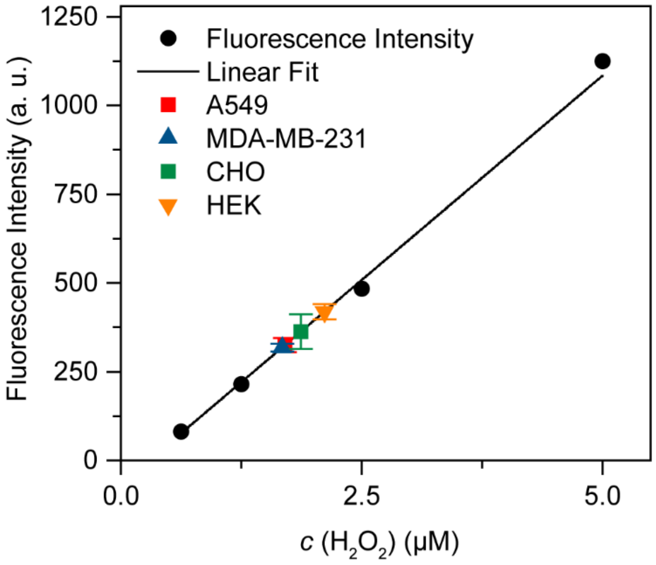

Figure S69. Standard curve for determination of the intracellular H_2O_2 concentration in A549, MDA-MB-231, CHO, and HEK cell lines. The intracellular H_2O_2 concentration is determined to be 1.70 μM in A549 cells, 1.67 μM in MDA cells, 1.87 μM in CHO cells, and 2.11 μM in HEK cells.

9 References

1. Pieszka, M.; Han, S.; Volkmann, C.; Graf, R.; Lieberwirth, I.; Landfester, K.; Ng, D. Y. W.; Weil, T. Controlled Supramolecular Assembly Inside Living Cells by Sequential Multistaged Chemical Reactions. *J. Am. Chem. Soc.* **2020**, *142*, 15780-15789.

2. Merrifield, R. B. Solid Phase Peptide Synthesis. I. The Synthesis of a Tetrapeptide. *J. Am. Chem. Soc.* **1963**, *85*, 2149-2154.
